# Aminobenzothiazole derivatives stabilize the thermolabile p53 cancer mutant Y220C and show anticancer activity in p53-Y220C cell lines

**DOI:** 10.1016/j.ejmech.2018.04.035

**Published:** 2018-05-25

**Authors:** Matthias G.J. Baud, Matthias R. Bauer, Lorena Verduci, Felix A. Dingler, Ketan J. Patel, Deeptee Horil Roy, Andreas C. Joerger, Alan R. Fersht

**Affiliations:** a Medical Research Council, Laboratory of Molecular Biology, Francis Crick Avenue, Cambridge, CB2 0QH, United Kingdom; b Chemistry, Faculty of Natural and Environmental Sciences, University of Southampton, Southampton, SO17 1BJ, UK; c German Cancer Consortium (DKTK), German Cancer Center (DKFZ), 69120 Heidelberg, Institute of Pharmaceutical Chemistry, Johann Wolfgang Goethe University, Max-von-Laue-Str. 9, Frankfurt am Main, 60438, Germany

**Keywords:** Mutant p53, Structure-based drug discovery, Anticancer therapy

## Abstract

Many cancers have the tumor suppressor p53 inactivated by mutation, making reactivation of mutant p53 with small molecules a promising strategy for the development of novel anticancer therapeutics. The oncogenic p53 mutation Y220C, which accounts for approximately 100,000 cancer cases per year, creates an extended surface crevice in the DNA-binding domain, which destabilizes p53 and causes denaturation and aggregation. Here, we describe the structure-guided design of a novel class of small-molecule Y220C stabilizers and the challenging synthetic routes developed in the process. The synthesized chemical probe **MB710**, an aminobenzothiazole derivative, binds tightly to the Y220C pocket and stabilizes p53-Y220C *in vitro*. **MB725**, an ethylamide analogue of **MB710**, induced selective viability reduction in several p53-Y220C cancer cell lines while being well tolerated in control cell lines. Reduction of viability correlated with increased and selective transcription of p53 target genes such as *BTG2*, *p21*, *PUMA*, *FAS*, *TNF*, and TNFRSF10B, which promote apoptosis and cell cycle arrest, suggesting compound-mediated transcriptional activation of the Y220C mutant. Our data provide a framework for the development of a class of potent, non-toxic compounds for reactivating the Y220C mutant in anticancer therapy.

## Highlights

• Proof-of-concept molecules stabilize the oncogenic protein p53-Y220C *in vitro*.
• Molecule MB725 shows selective anticancer activity in p53-Y220C cancer cells.
• Anticancer activity correlates with enhancement of p53 signaling by MB725.

## Graphical abstract

**Figure:**
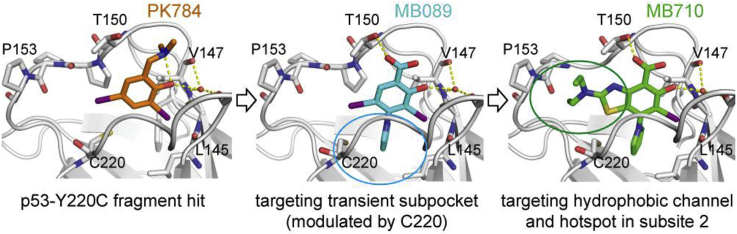

## 1 Introduction

The tumor suppressor protein p53 plays a pivotal role in several critical cellular processes, including cell-cycle regulation, DNA repair and apoptosis. It exerts its tumor suppressor function through complex and intricate regulatory processes mediated by its association with a wide range of cellular effectors [[1], [2], [3]]. Virtually all tumors display impaired or abrogated p53 signaling, making the p53 pathway a prime target for anticancer drug development [[4], [5], [6], [7], [8], [9]]. Such impairment can result from several factors, including mutations in the *TP53* gene [10], elevated levels of its negative regulators (e.g., MDM2 or MDM4) [[11], [12], [13], [14]], and epigenetic events [15,16]. p53 is inactivated directly by mutation in about 50% of all cancers, with the majority of point mutations occurring in its DNA-binding domain (DBD) [17,18], which affects its DNA binding and/or thermodynamic stability. About one third of these mutants are simply unstable and undergo rapid denaturation under physiological conditions [[18], [19], [20], [21], [22]]. Importantly, many of these destabilized p53 mutants display transcriptional activity at sub-physiological temperatures [23,24], suggesting that their function could be restored by binding of small molecules that stabilize the structure [[25], [26], [27], [28]].

**Fig. 1:**
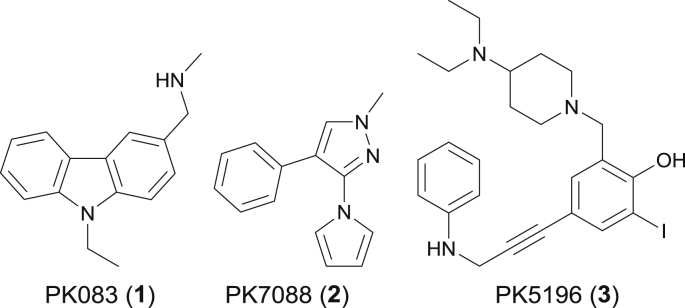
Structures of representative small molecules targeting the p53 cancer mutant Y220C.

The oncogenic Y220C mutant provides a particularly suitable test case for the development of small-molecule stabilizers. It is the ninth most frequent p53 missense mutant found in cancer and is associated with approximately 100,000 new cancer cases per year worldwide [21,22,29]. Mutation of Tyr220 to Cys creates a narrow, hydrophobic pocket on the surface of the p53 DBD that reduces its thermal stability by approximately 4 kcal/mol [20,26]. While wild-type p53 is moderately stable, melting at 44 °C [19,30,31], the Y220C mutant rapidly unfolds under physiological conditions, which effectively abrogates p53 signaling and drives tumorigenesis [21]. Importantly, the mutation-induced crevice is distant from the p53 surfaces involved in DNA recognition or protein-protein interactions, allowing for the development of stabilizing small molecules without interfering with binding of its natural substrates. Using *in silico* methods, fragment-based screening and structure-guided design, we have developed a series of small molecules that bind to the Y220C pocket, including the *N*-ethylcarbazole PK083 (**1**) [25,32], pyrazole-based compound PK7088 (**2**) [27] and iodophenol derivative PK5196 (**3**) [28] (Fig. 1).

**Fig. 2:**
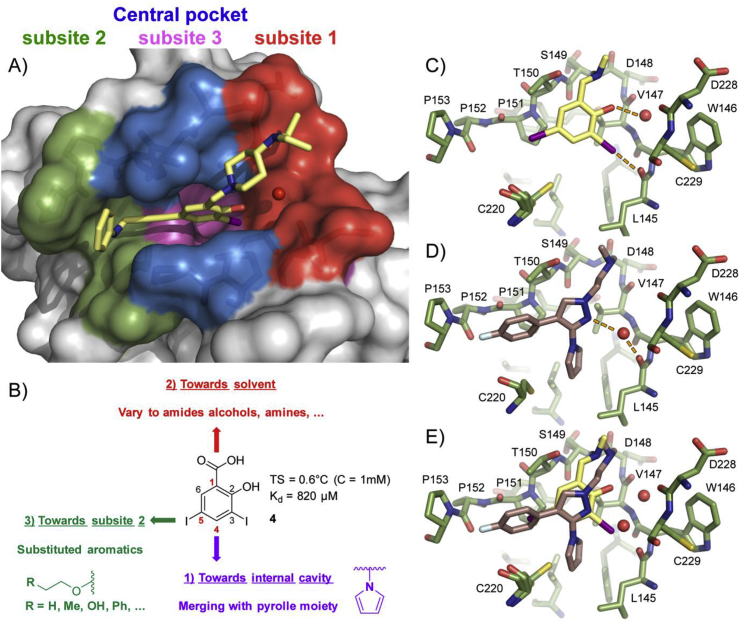
Strategy for the structure-based design of improved Y220C mutant stabilizers. **A)** Crystal structure of **3** (light yellow sticks) bound to Y220C (PDB: 4AGQ) [28]. The different subsites referred to throughout the text are highlighted. A structural water molecule interacting with **3** is shown as a red sphere. **B)** Structure of diiodosalicylic acid **4**. The 3 positions selected for derivatization are shown. **C)** Crystal structure of a benzylamine derivative of **4** (light yellow sticks) bound to Y220C (green sticks, PDB: 4AGL) [28]. The key halogen bond to Leu145 and the hydrogen bond between the phenol moiety and a structural water molecule are shown (dashed orange lines). **D)** Crystal structure of a derivative of **2** (pink sticks) bound to Y220C (green sticks, PDB: 3ZME) [27]. The hydrogen bond of the pyrazole moiety to a structural water molecule is shown (dashed orange lines). **E)** Overlay of both structures suggests a possible merging approach for the introduction of a pyrrole at C4 and introduction of an aromatic ring at C5.

The *N*-ethylcarbazole derivative PK083 (**1**) binds to the Y220C pocket with a dissociation constant (*K*_d_) of 140 μM, increases the thermostability of the mutant protein and slows down its aggregation *in vitro* [25]. The pyrrole-substituted pyrazole derivative PK7088 (**2**) binds with a similar affinity and displays promising cellular activity in cancer cell lines carrying the Y220C mutation, e.g., induction of caspases and upregulation of p53 target genes *PUMA* and *NOXA* [27]. However, relatively high concentrations of the compound (up to 200 μM) are required to observe these effects, and the possibility of off-target effects contributing to the observed response cannot be ruled out completely. A biophysical screen of a halogen-enriched fragment library identified the 2-iodophenol moiety as a potent scaffold to target the Y220C pocket. Binding of **3** and other iodophenol derivatives is driven by a strong halogen bond between the iodine atom and the carbonyl oxygen of Leu145 [33]. Targeting additional subsites of the binding pocket led to the development of PK5196 (**3)**, which displays a *K*_d_ of 10 μM and raises the *T*_m_ of the protein by almost 4 °C under saturating conditions (Fig. 1, Fig. 2A) [28]. Although compounds **1**–**3** and derivatives stabilize Y220C, their modest affinities as well as issues of stability or toxicity (e.g., flatness or relatively unstable acetylene groups) have hampered biological studies and their potential use as drug candidates. Improving the affinity of future lead compounds, in addition to tuning of their physico-chemical properties, will therefore be crucial for fully exploiting the therapeutic potential of small-molecule stabilizers of Y220C in cancer cells.

Here, we report the design, synthesis, biophysical and biological evaluation of a new class of compounds with improved properties. We revisited **3** and the iodophenol lead series because they have the highest affinity so far [28]. Optimization of scaffold substitution for targeting different subsites of the binding pocket (Fig. 2A) may offer a way to improve the affinity and physico-chemical properties of **3**. Further, there is a transiently open sub-pocket, subsite 3, which is modulated by the conformation of Cys220 (Fig. 2A) [34], and we hypothesized that targeting subsite 3 might increase affinity of the iodophenol series. Finally, we present preliminary data on the biological activity of our new ligands in cancer cells. We show that one particular aminobenzothiazole derivative selectively reduced viability of Y220C-mutated cell lines, NUGC3, BXPC-3 and HUH-7, which correlated with selective upregulation of proapoptotic and cell cycle arrest p53 target genes *BTG2*, *p21*, *PUMA*, *FAS*, *TNF*, and *TNFRSF10B* in NUGC3.

## 2 Results and discussion

### 2.1 Library design strategy

We based our design strategy on 2-hydroxy-3,5-diiodobenzoic acid (**4**), which we discovered in a fragment screen (Fig. 2B) [28]. The aromatic ring of **4** is flanked by Val147, Pro151, Pro222 and Pro223 and engages in extensive hydrophobic contacts and CH-π interactions. The iodine atom at C3 forms a halogen bond with the carbonyl oxygen of Leu145, and the hydroxyl group at C2 hydrogen bonds with a structural water molecule bridging the backbones of Val147 and Asp228. The carboxylate at C1 is solvent exposed and forms a hydrogen bond with Thr150. Hydrophobic contacts between the iodine atom at C5 and the hydrophobic channel towards subsite 2 add to the affinity. With a *K*_d_ of 820 μM, **4** displays a high ligand efficiency (LE = 0.35). It is water soluble and offers a variety of vectors potentially exploitable for growing the fragment into different subsites. Our design strategies focused on three main aspects: first, substitution at C4 would allow for new favorable interactions within the hydrophobic subsite 3 pocket (Fig. 2A, pink); second, the carboxylate moiety at C1 represents an ideal handle for probing potential interactions with neighboring solvent-exposed residues (subsite 1, red); and, third, that substitution at C5 should allow growing within the narrow hydrophobic channel leading to subsite 2 and engage in additional interactions.

An alignment of Y220C crystal structures of several iodophenol derivatives (PDB: 4AGL, 4AGM, 4AGN, 4AGO, and 4AGP) [28] with that of bound pyrrole-substituted pyrazole derivative PK7088 (**2**, PDB: 3ZME) [27] revealed a possible merging of the iodophenol scaffold and the pyrrole moiety at C4 (Fig. 2C–E). In addition, the carboxylic acid at C1 should give access to a variety of other functional groups at this position, including esters, alcohols, amides, hydroxamic acids and amines. The same alignment (Fig. 2C–E) highlighted a possible merging of the 4-fluorophenyl ring of **2** with the central iodinated scaffold of **4**, therefore suggesting an aromatic ring at C5 as a possible substituent to grow **4** into subsite 2. Alternatively, a hydrophobic, flexible linker could provide an alternative way of accessing this subsite. While a flexible linker might hamper potency through unfavorable entropic contributions compared with a rigid acetylene moiety, a chemically stable linker is crucial for future applications *in vivo*. We devised a library of compounds based on an oxyether linker and varying by the nature of the R group (Fig. 2B). We hypothesized that an oxyether linker should be chemically stable in a cellular environment, and should provide some degree of conformational restriction to the ligand side chain, therefore reducing entropic penalty of binding to a certain extent. Initially, an oxygen atom was selected based on synthetic tractability of the library (see chemistry section). R groups were chosen so as to probe the shape, hydrophobics and polar interactions within subsite 2, and modelled into the x-ray structure of **9** [34] using Maestro [35], giving attention to potential clashes with the binding pocket, low energy conformations of the ligand, and specific molecular interactions with diverse residues within the Y220C pocket. Visual inspection of available iodophenol bound structures as well as docking studies with Glide [[35], [36], [37], [38]] (Fig. S1) suggested an -OEt side chain should be accommodated within the hydrophobic channel and, therefore, represent a good starting point for investigation. Further growing would create additional interactions within subsite 2. For example, an alcohol or trifluoromethyl group could engage the backbone carbonyls of Pro151, Pro152 and Cys220, while a phenyl group could engage in extensive hydrophobic interactions within the narrow channel connecting the central cavity and subsite 2.

### 2.2 Chemistry

**Scheme 1:**
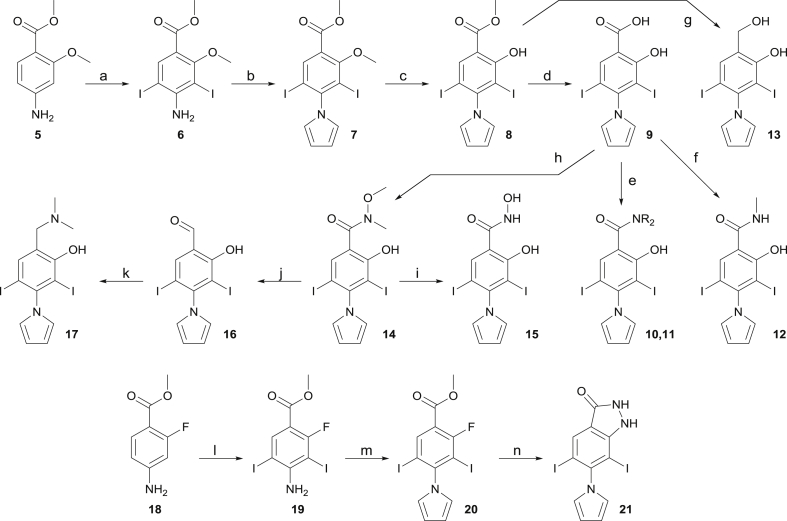
Synthesis of target analogues through functionalization at C1 and C4 for exploration of subsite 1 and subsite 3.^a^ ^a^Conditions: (a) NIS, AcOH, rt, quant.; (b) 2,5-dimethoxytetrahydrofuran, AcOH, 80 °C, quant.; (c) BBr_3_, CH_2_Cl_2_, 0 °C, 89%; (d) NaOH, THF/H_2_O, rt, quant.; (e) EDCI, NHS, CHCl_3_, amine, rt, 14–26%; (f) methylamine, H_2_O/MeOH, 84%; (g) NaBH_4_, CH_2_Cl_2_/MeOH, rt, 53%; (h) EDCI, HOBt, *N*,*O*-dimethylhydroxylamine hydrochloride, DIPEA, CH_2_Cl_2_, rt, 54%; (i) hydroxylamine, THF/H_2_O, rt, 85%; (j) DIBAL-H, THF, −78 °C to rt, 54%; (k) dimethylamine, AcOH, NaB(OAc)_3_H, CH_2_Cl_2_, rt, 67%; (l) NIS, AcOH, rt, quant.; (m) 2,5-dimethoxytetrahydrofuran, AcOH, 80 °C, quant.; (n) hydrazine monohydrate, Et_3_N, CH_2_Cl_2_/EtOH, 75 °C, 51%. Refer to Table 1 for R groups.

**Scheme 2:**
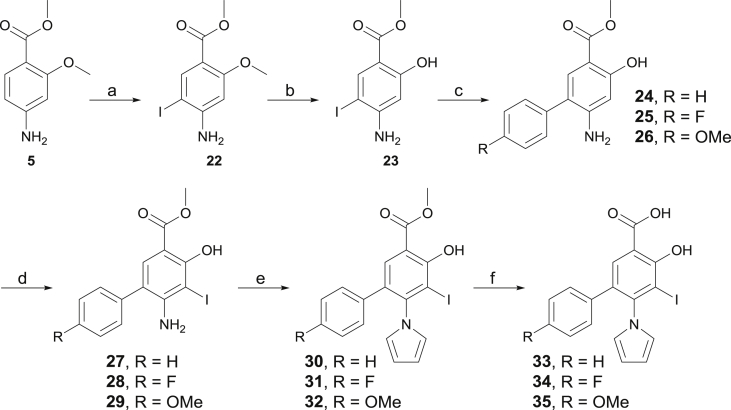
Synthesis of biaryl analogues **33**–**35** by cross-coupling at C5 for exploration of subsite 2.^a^ ^a^Conditions: (a) NIS, MeCN, 0 °C to rt, quant.; (b) BBr_3_, CH_2_Cl_2_, 0 °C, 59%; (c) ArB(OH)_2_, Pd(PPh_3_)_4_, Cs_2_CO_3_, dioxane/H_2_O, 80 °C, 49–80%; (d) NIS, MeCN, 0 °C, 60–90%; (e) 2,5-dimethoxytetrahydrofuran, AcOH, 80 °C, 76–96% (f) NaOH, THF/H_2_O, rt, quant.

**Scheme 3:**
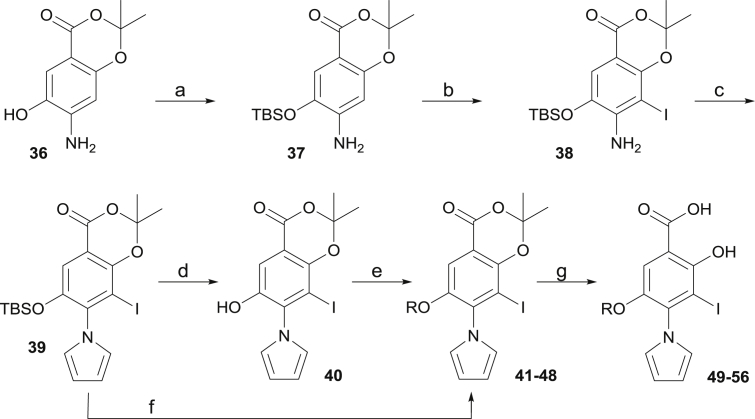
Synthesis of analogues **49–56** bearing an oxyether linker for exploration of subsite 2.^a^ ^a^Conditions: (a) TBSCl, imidazole, CH_2_Cl_2_, 0 °C to rt, 88%; (b) NIS, MeCN, 0 °C to rt, quant.; (c) 2,5-dimethoxytetrahydrofuran, AcOH, 80 °C, quant.; (d) TBAF, DMF, rt, quant.; (e) ROH, PPh_3_, DEAD, THF, rt, 47–95%; (f) RI, KF, DMF, rt, 70–90%; (g) KOH, H_2_O/THF, rt, 71–98%.

The synthesis of our compound library is shown in Scheme 1, Scheme 2, Scheme 3. Methyl 4-amino-2-methoxybenzoate **5** was converted to diiodinated derivative **6** in quantitative yield. Further treatment with 2,5-dimethoxytetrahydrofuran allowed for the introduction of the pyrrole moiety towards **7** in quantitative yield. Methoxy deprotection using BBr_3_ afforded phenol derivative **8** in 89% yield, which was used as a precursor for further derivatization. **8** could be converted in quantitative yield to the corresponding carboxylic acid **9** originating from the merging strategy (Fig. 2C–E). Acid **9** could be converted to amide derivatives **10**–**11** in low yield with EDCI/NHS. Direct amidation of ester **8** with methylamine towards *N*-methylamide derivative **12** proceeded in 84% yield. Reduction of ester **8** afforded the corresponding benzyl alcohol derivative **13** in moderate yield using NaBH_4_ in a CH_2_Cl_2_/MeOH mixture. Acid **9** could be converted to the corresponding Weinreb amide **14** in moderate yield. The latter could be converted to hydroxamic acid derivative **15**, or reduced to aldehyde **16**. Reductive amination of the latter afforded dimethylamino derivative **17**. Double iodination of methyl 4-amino-2-fluorobenzote **18** afforded intermediate **19**, and conversion to pyrrole analogue **20** proceeded quantitatively. Treatment with hydrazine delivered cyclic derivative **21** in moderate yield. **21** retains a hydrogen bond acceptor at C1 and hydrogen bond donor at C2, hence mimicking the ortho-hydroxy carboxylate motif of other analogues. It was envisaged that a cyclic structure would allow restricting bond rotation at C1 and C2, while engaging in the same interactions with the receptor.

The introduction of aromatic substituents at C5 is described in Scheme 2. Selective monoiodination of methyl 4-amino-2-methoxybenzoate **5** afforded compound **22** in quantitative yield. Further methoxy deprotection afforded aminophenol **23** in moderate yield. Suzuki cross-coupling between **23** and the appropriate boronic acid afforded biaryls **24**–**26** in moderate (R = OMe, 49%) to high yield (R = H, 80%). Further iodination with NIS afforded **27**–**29**, and introduction of the pyrrole afforded **30**–**32** in good to excellent yield. Final basic hydrolysis afforded products **33**–**35** quantitatively.

The introduction of oxyether side chains at C5 to explore subsite 2 is described in Scheme 3. Aminophenol **36** was prepared as previously reported [39]. TBS protection of the latter afforded compound **37** in 88% yield. Further iodination afforded compound **38** quantitatively. Introduction of the pyrrole proceeded in quantitative yield and afforded precursor **39**. Pleasingly, the TBS group remained stable in these conditions, and deprotection could not be observed, in line with previous reports [40]. **39** could be prepared on a 6.5 g scale, demonstrating the robustness and scalability of this synthetic route. Silyl deprotection using TBAF in DMF afforded phenol **40** quantitatively. Mitsunobu reaction of the latter with the appropriate alcohol afforded functionalized oxyether derivatives **41**–**48**. In some cases (methyl, ethyl), treatment of **39** with the appropriate alkyl iodide and potassium fluoride in DMF afforded the *O*-alkylated product in high yield in a single step. However, the yields dramatically decreased with the length and steric demand imposed by larger substituents (e.g. iBu, data not shown). Final hydrolysis afforded products **49**–**56**.

### 2.3 Biophysical studies, structural characterization and further optimization

The binding of our library of analogues to Y220C was subsequently evaluated biophysically. Thermal stabilization of Y220C was assessed by DSF at 250 μM ligand concentration, and *K*_d_s of selected analogues with the highest thermal shifts (Table 1, Table S1, Fig. S5) were determined by ^1^H-^15^N heteronuclear single quantum coherence (HSQC) NMR or isothermal titration calorimetry (ITC) (Table 1, Table S1). There is generally a good correlation between these two parameters when considering p53-Y220C as a target, and usually the more potent the ligand the higher the stabilization (Fig. S6).

**Table 1:**
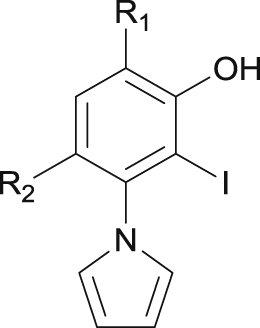
Thermal stabilization and binding affinities of Y220C-mutant binding compounds as assessed by DSF and ITC/NMR.

Cpd	R_1_	R_2_	ΔT_m_ (°C)^a^ [ligand] = 250 μM	*K*_d_ (μM)
**4**			ND	820^b^
**8**	CO_2_Me	I	−0.1^c^	ND
**9**	CO_2_H	I	1.8^d^	21^d^
**10**		I	1.3	ND^b^
**11**		I	1.2	30
**12**	C(O)NHMe	I	0.9	70
**13**	CH_2_OH	I	1.3	20^e^
**15**	C(O)NHOH	I	0.8	139
**17**	CH_2_NMe_2_	I	0.9	60
**21**	C(O)NHNH	I	1.5	50
**33**	COOH	Ph	1.0	306
**34**	COOH	4-F-Ph	0.8	113
**35**	COOH	4-OMe-Ph	1.0	122
**49**	COOH	OEt	1.3	63
**50**	COOH	OPr	1.7	22
**51**	COOH		1.8	33
**52**	COOH	O-*n*Bu	1.4	30^e^
**53**	COOH		1.4	108
**54**	COOH		0.6	726
**55**	COOH		1.0	116
**56**	COOH		1.4	30
**60**	COOH		2.2	14

a Measured by DSF using 8 μM protein and 10 × SYPRO Orange with ΔT_m_ values calculated as the average of quadruplicate measurements.

b Data taken from Ref. [28].

c Poor solubility.

d Data taken from Ref. [34].

e Determined by HSQC-NMR with *K*_d_ values calculated as the average of at least three fits of peaks that are shifted by the compound as described previously [28].

**Fig. 3:**
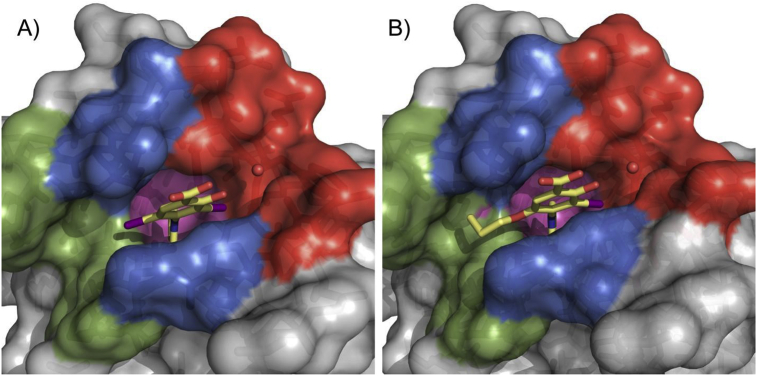
Co**-**crystal structure of Y220C (surface representation) with A) **9**; B) **50**. Different subsites are colored as in Fig. 2.

Substitution at C4, merging with a pyrrole moiety: Introducing a pyrrole moiety at C5 had a major effect on affinity. Pyrrole substituted analogue **9** induces a thermal shift of 1.8 °C and displays a *K*_d_ of 21 μM (Table 1) and a high ligand efficiency (LE = 0.38) [34], making it 40-fold more potent than parent fragment **4**, thereby validating our initial merging strategy (Fig. 1C–E). The crystal structure of Y220C with bound **9** (PDB: 5AOJ; Fig. 3A) unambiguously confirmed the expected binding mode, and revealed a high positional overlap of the pyrrole moiety and the central iodophenol scaffold with bound **2** and **3**, respectively (Fig. 2C–E) [34]. Detailed analysis of this structure shows extensive hydrophobic contacts between the pyrrole moiety and side chains of Phe109, Leu145, Val147, Pro151, Val157, Cys220, and Thr230. CH-π interaction between the Cys220 side chain methylene and the pyrrole system, in addition to n_S_ → π*_pyrrole_ might contribute to the potency gain. Importantly, the pyrrole moiety induced the side chain of Cys220 to adopt an alternative (“flipped”) conformation, which is not observed in the apo crystal structure but in several other ligand complexes [34,41]. The steric hindrance imposed by the two iodine atoms at C3 and C5 induces conformational restriction to the pyrrole ring and is likely to reduce unfavorable entropy. This allows the two ring systems to twist (dihedral angle) by about 80°, thereby placing the pyrrole ring in an ideal binding conformation. The inductive effect of the pyrrole at C4 might also modulate the strength of the halogen bond and contribute to the binding enthalpy [33]. The solvent-exposed carboxylate functionality at C1 forms a hydrogen bond with the side chain of Thr150.

Substitution at C1, towards the solvent: All substitutions at C1 (Table 1) lowered the thermal shifts. Substituting the carboxylic functionality by amides (**10**–**12**) induced lower thermal shifts and potency. Of note, secondary amides **10** and **12** were substantially less soluble than carboxylic acid **9**. The exception was benzylic alcohol derivative **13**, which had a similar potency as the parent acid **9** (thermal shift of 1.3 °C and *K*_d_ of 20 μM). Hydroxamic acid **15** and dimethylamino derivative **17** had significantly lower potencies and thermal shifts, with respective *K*_d_s of 139 μM and 60 μM. The crystal structures of Y220C with bound **9** and **13** (Fig. 3A, Fig. S7, Table S2) show a similar hydrogen bonding with Thr150 likely contributing to their improved potency. In contrast, hydrogen bonding with Thr150 was not observed in the co-crystal structure of bound amide derivative **11** (Fig. S7, Table S2), probably resulting from steric constraints, imposing a dihedral angle θ ≈ 70° between the tertiary amide and the phenyl ring. Restricted analogue **21**, containing a dihydroindazolone motif, was also less potent (thermal shift of 1.5 °C and *K*_d_ of 50 μM) than **9**. Accordingly, a carboxylic acid was selected as the substituent of choice at C1 for further derivatization due to the increased potency and solubility it conveys to the scaffold. We have previously shown that carefully chosen aliphatic diamines at C1 can increase affinity: in particular, the diamine side chain of **3** (*K*_d_ = 10 μM, Fig. 1, Fig. 2A) and related derivatives increases the affinity ca. 10-fold relative to their carboxylate analogues [28]. However, 2-hydroxybenzylamine and 2-hydroxybenzyl alcohol derivatives are usually reported as pan-assay interference compounds (PAINs) due to their moderate stability and are usually discarded during the drug discovery process [42]. While higher potency might be achievable with such polyamines, the chemical robustness of the salicylate motif of **9** and the high solubility it confers to our lead series was considered advantageous for further derivatization and structural studies.

Substitution at C5, aromatics and flexible linkers towards subsite 2: Substituting the iodine of **9** at C5 with a phenyl, an electron-poor 4-fluorophenyl or an electron-rich 4-methoxyphenyl resulted in less potent compounds in each case, with thermal shifts of **33**–**35** in the range of 0.8–1.0 °C (Table 1), translating into *K*_d_s > 100 μM. The reason for this is unclear, but is likely to result from steric clashes arising from the dihedral angle between the two phenyl rings (θ ≈ 45°), as revealed by the crystal structure of bound **34** (Fig. S7, Table S2). Despite its reduced potency, the binding mode of **34** was very similar to that of **9** and consistent with our initial merging strategy (Fig. 2C–E). Simple ethyl substituted analogue **49** retained binding despite the flexibility of the side chain. **49** induced a thermal shift of 1.3 °C and displayed a *K*_d_ of 63 μM, therefore only 3 times less potent than the parent iodine (1.8 °C, 21 μM). Interestingly, the one carbon longer propyl analogue **50** induced higher thermal shift of 1.7 °C and displayed a *K*_d_ of 22 μM, 3 times more potent than **49** and similarly potent as the parent diiodinated analogue **9**. Allyl derivative **51** displayed a very similar profile to that of propyl derivative **50**. Trifluoromethylated analogue **53** and terminal alcohol **50** were initially designed to probe potential multipolar interactions with backbone carbonyls of proximal Pro152, Pro153 and Cys220. Both **53** and **54** displayed decreased potency compared with the propyl analogue **50**, suggesting hydrophobic alkyl substituents to be optimal for potency gain within subsite 2. Intriguingly, all attempts to grow further by increasing the chain length resulted in decreased affinity, with *K*_d_s > 100 μM in each case and low thermal shifts, as illustrated by *n*-butyl **52**, phenylethyl **55** and branched isobutyl **56**. The reason for this observation is unclear, as visual inspection of the binding site suggests that such substituents should be accommodated within subsite 2. We obtained high resolution crystal structures of **50** (Pr), **52** (*n*-Bu) and **55** (phenylethyl) bound to the Y220C pocket (Fig. 3B, Fig. S7, Table S2). In each case, the binding mode of the central iodophenol scaffold was nearly identical to that of parent compound **9**. The propyl chain occupies the narrow, hydrophobic channel formed by Pro151, Pro152, Pro153 and Pro222. The terminal methyl group of **50** makes hydrophobic contacts with a narrow cleft formed by Pro151, Pro152, Pro153 and Thr155. The side chains of **52** and **55** occupy very similar positions, but extend further in the binding pocket and make additional hydrophobic contacts with Pro153 and Pro222. In particular, the positioning of the phenyl group of **52** between Pro153 and Pro222 is consistent with the binding mode of a range of previously reported aromatic, fragment-size molecules binding at this subsite and engaging in CH-π interactions with these residues [34,41]. We recently reported on the structural plasticity of the p53-Y220C binding pocket [34]. This study highlighted the importance of water networks and loop flexibility on the dynamics of the binding pocket. In particular, an extensive analysis of Y220C apo and fragment bound crystal structures in combination with molecular dynamics simulations revealed significant movements of the loop containing residues 220–230 flanking subsite 2. This in turn is likely to influence the structural dynamics of subsite 2, in particular, the distance between Pro153 and Pro222, which might explain the reduced potency of **52**, **55** and **56** aimed at targeting these residues.

**Fig. 4:**
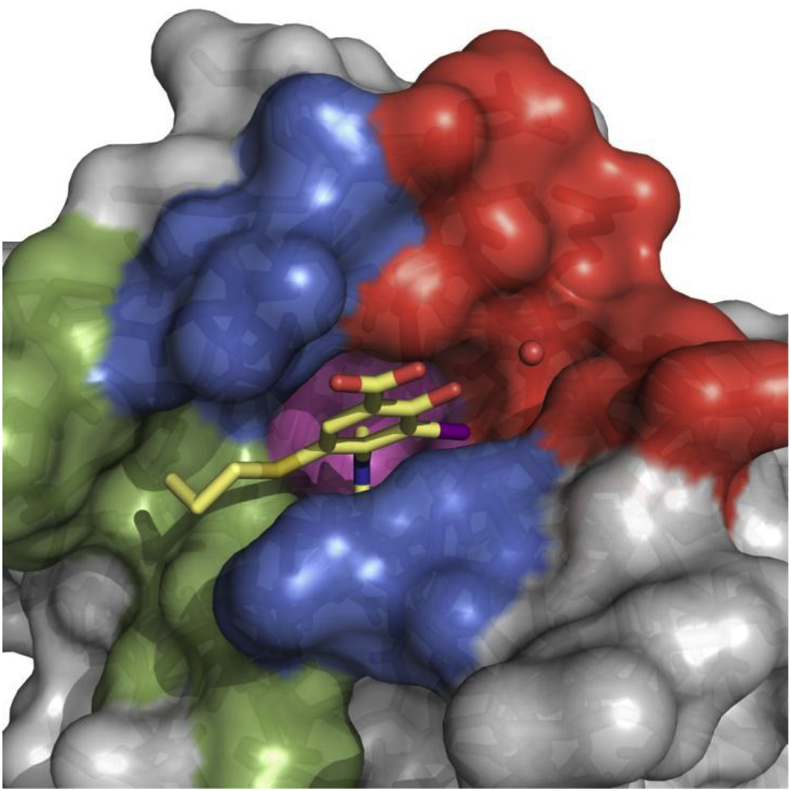
Crystal structure of Y220C (surface representation) in complex with **60**. Different subsites are highlighted in the same colors as in Fig. 2.

**Scheme 4:**
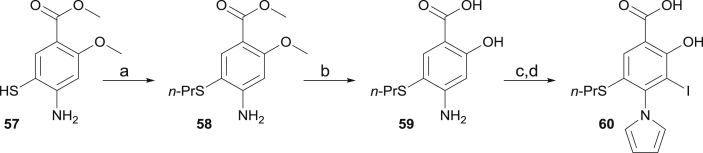
Synthesis of thioether-functionalized derivative **60**.^a^ ^a^Conditions: (a) *n*-PrI, Cs_2_CO_3_, DMF, 0 °C to rt, 41%; (b) BBr_3_, CH_2_Cl_2_, 0 °C, 77%; (c) NIS, MeCN, 0 °C; (d) 2,5-dimethoxytetrahydrofuran, AcOH, 80 °C, 30% over 2 steps.

Further optimization: We further hypothesized that replacing the oxygen atom of the oxyether linker by a sulphur atom could represent an additional source of affinity through enhanced shape complementarity and hydrophobic contacts. This is supported by the high potency of parent compound **9** bearing an iodine atom at this position that engages in extensive hydrophobic contacts within the channel. The synthesis of the target is described in Scheme 4. *S*-alkylation of aminothiol **57** afforded *S*-propyl intermediate **58**. Further treatment with 1.1 equivalent of BBr_3_ led to the deprotection of both the methoxy group and the methylester, and afforded **59**. Further iodination and pyrrole formation afforded the target compound **60**. Gratifyingly, **60** displayed an improved thermal shift of 2.2 °C, which translated into a *K*_d_ of 14 μM (Table 1). While the affinity gain was admittedly modest (<2 fold), both assays provide evidence that sulphur is the optimal heteroatom at this position for potency, due to its hydrophobic character and relatively large apolar surface area (compared to O) but also due to its valence (compared to I) that affords an additional vector to further explore subsite 2. Indeed, replacing sulphur by nitrogen (S to NH) resulted in an inactive compound (data not shown). We determined the crystal structure of Y220C with bound **55** (Fig. 4, Table S2), confirming the positioning of the sulphur atom within the narrow channel. The propyl chain showed a nearly identical binding mode as that of **50**.

**Scheme 5:**
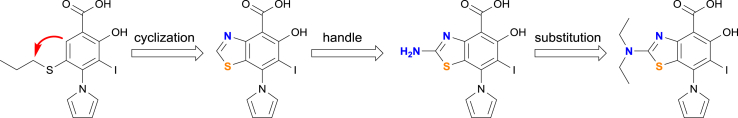
Design strategy towards substituted aminobenzothiazole derivatives.

We subsequently devised a cyclization strategy based on a fused heterocycle at C5 and C6 (Scheme 5). We hypothesized that 1) introduction of a 5-membered sulphur containing heterocycle such as a thiazole at C5-C6 would allow enhancing hydrophobic contacts with neighboring residues Pro151 and Pro222; 2) such a strategy should be advantageous to reduce unfavorable entropic terms through preventing rotation around the two C-S bonds; and 3) an exocyclic amino group would represent a good handle for further derivatization through *N*-alkylation; and finally 4) upon visual inspection of the binding site, we envisaged that an ethyl group would be ideal to achieve shape complementarity within subsite 2, therefore mimicking the terminal methyl group of non-cyclized analogues **50** and **60** (Fig. 3B–C).

**Scheme 6:**
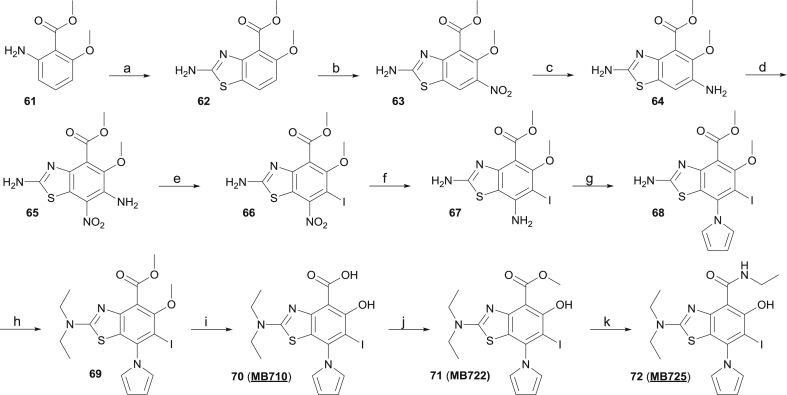
Synthesis of substituted aminobenzothiazole derivative **70**–**72**.^a^ ^a^Conditions: (a) KSCN, Br_2_, AcOH, 10 °C, 43%; (b) HNO_3_,0 °C, 91%; (c) SnCl_2_·2H_2_O, CH_2_Cl_2_/AcOEt, 0 °C to rt, 58%; (d) KNO_3_, TFA, rt, 80%; (e) NaNO_2_, aq. HCl, 0 °C, then KI, rt, 80%; (f) Fe, AcOH, 50 °C, 92%; (g) 2,5-dimethoxytetrahydrofuran, AcOH, 120 °C, 19%; (h) EtI, K_2_CO_3_, DMF, 60 °C, 48%; (i) BBr_3_, CH_2_Cl_2_, 0 °C to rt, 79%; (j) DCC, DMAP, MeOH, rt, 92%; (k) 70% aq. EtNH_2_, rt, 95%.

The synthetic route towards the target aminobenzothiazole derivative is shown in Scheme 6. Treatment of aniline derivative **61** with potassium thiocyanate and bromine in acetic acid afforded aminobenzothiazole **62** in moderate yield. Further nitration could be achieved in fuming nitric acid [43] at 0 °C, delivering intermediate **63**. The latter was reduced with SnCl_2_·2H_2_O and afforded anilino derivative **64**. Further nitration towards **65** was achieved using KNO_3_ in TFA. Attempts to nitrate in conditions used in step (b) led to degradation. Diazotation and treatment with potassium iodide towards iodinated intermediate **66** proceeded in high yield. Further nitro reduction was achieved using iron in acetic acid, affording **67**. Introduction of the pyrrole could be achieved in low yield through reduction of the nitro group and treatment of the intermediate aniline derivative with 2,5-dimethoxytetrahydrofuran. We attributed the low yield for the conversion of **67** to **68** to the high steric demand around the aniline nitrogen, in addition to the deactivating effect of the other aromatic, electron-withdrawing substituents. Of note, the amino group of the thiazole ring was not reactive in these conditions, preventing further selectivity issues. Alkylation of **68** with ethyl iodide in DMF afforded **69** in moderate yield. The ester and methoxy groups were subsequently and simultaneously deprotected using boron tribromide, revealing the corresponding salicylic acid unit of **70**. We envisaged that simple ester and amide derivatives of **70** would be of interest for cell-based assays, as masking its carboxylic acid moiety might influence cell permeability of this series and provide additional cellular structure-activity relationship. Selective methoxy deprotection proved unsuccessful using BBr_3_ or BCl_3_, although double deprotection could be achieved in good yield by using more than two equivalents of reagent. Mild esterification of acid **70** with DCC/DMAP in MeOH provided the methylester derivative **71** in high yield. The latter could be converted in high yield to the corresponding ethylamide **72** by treatment with aqueous ethylamine in MeOH.

**Fig. 5:**
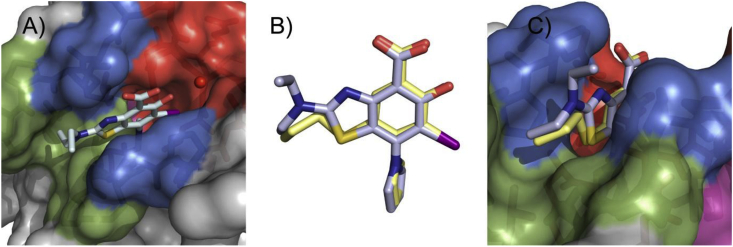
Crystal structure of **MB710** bound to Y220C. A) Top view of the binding pocket with bound **MB710** shown as a light blue stick model. The conserved structural water molecule interacting with **MB710** is shown as a red sphere. B) Superimposed structures of bound **MB710** (light blue sticks) and **60** (light yellow sticks) shows the conservation of the ligand binding mode. C) The terminal methyl group of **MB710** (light yellow) occupies a nearly identical position to that of **60** (light blue), validating the cyclization strategy.

Pleasingly, new chemical probe **MB710** (**70**) stabilized the Y220C mutant by 2.0 °C at 250 μM in DSF measurements and bound to the mutant protein with a *K*_d_ of 4 μM (ITC, Fig. S8), therefore showing a clear improvement over compounds **9** and **50**. Methylester **MB722** (**71**) and amide derivative **MB725 (72)** displayed reduced solubility and could not be accurately titrated at concentrations required for biophysical assessment. We further determined a high-resolution crystal structure of Y220C in complex with **MB710** (Fig. 5). The 5-Hydroxy-6-iodobenzothiazole scaffold of **MB710** binds in the pocket in a virtually identical manner to that of thioether **60**. Interestingly, both compounds interact with the narrow hydrophobic hotspot formed by Pro151, Pro152, Pro153 and Thr155 in subsite 2 via their terminal methyl groups. The observed binding-affinity increase of **MB710** may be explained by its more favorable entropic term due to higher conformational restriction and additional hydrophobic contacts with Pro151, Pro222, and Thr150. Overall, new chemical probe **MB710** displays a 200-fold increase in affinity compared to starting fragment **4**.

### 2.4 Biological assessment

**Fig. 6:**
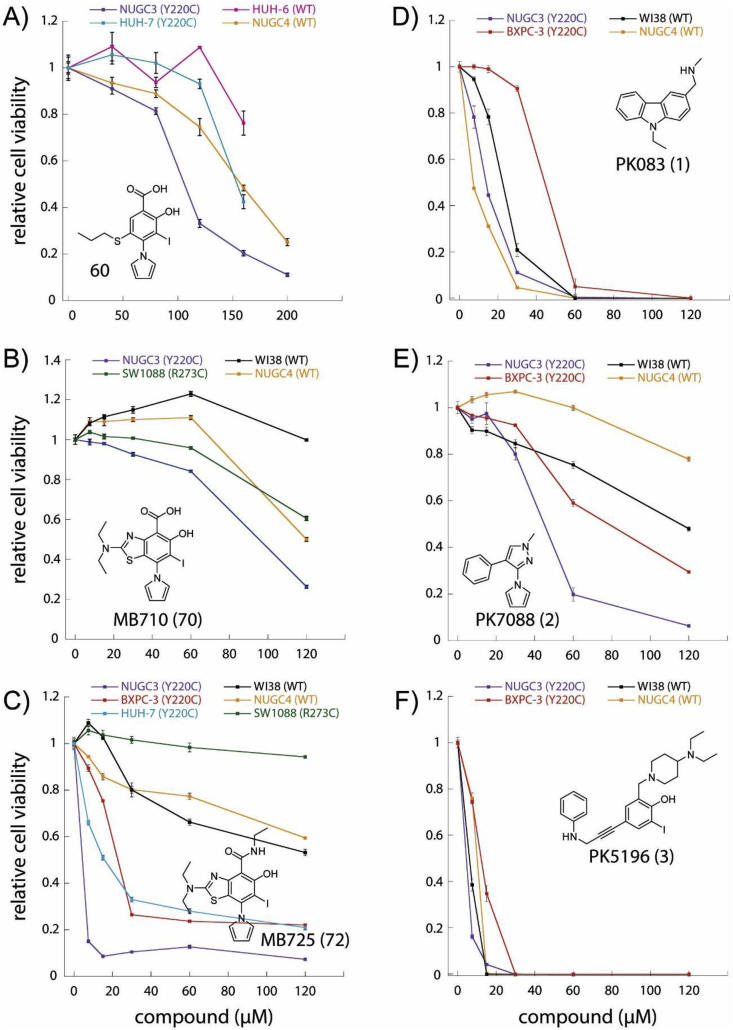
Effects of Y220C binders on cancer cell viability. Relative cell viability (Y-axis) of representative cell lines after 72 h treatment with increasing concentrations (X-axis, μM) of **60** (A), **MB710** (B), **MB725** (C), and previously reported lead compounds PK083 (D), PK7088 (E) and PK5196 (F). The cell line employed in each experiment and their respective p53 status are shown. Aminobenzothiazole **MB725** shows strong and selective viability reduction in the p53-Y220C cancer cell lines BXPC-3, HUH-7, and NUGC3 at concentrations below 40 μM, while maintaining relatively low toxicity in the same concentration range in the p53-R273C mutant cell line SW1088, and the p53 WT cell lines WI38 (normal fibroblast cell line) and NUGC4. Cell viability was measured in quadruplicate and normalized against the values of blank (viability = 1) and no cell (viability = 0) controls. Data are shown as mean ± SEM.

We performed initial cell viability experiments with representative cancer cell lines (Table S3 for cell lines description) containing p53 WT (NUGC4, HUH-6), mutant p53 (NUGC3, HUH-7, SW1088, BXPC-3), and a non-cancerous fibroblast cell line (WI38) following 72 h treatment with **60**, **MB710**, **MB725**, and previously reported model compounds **1**–**3** (Fig. 6, Table 2). We expected our compounds to show a strong cell viability reduction in cancer cell lines containing the Y220C mutation (NUGC3, HUH-7, BXPC-3), while showing relatively low or no effect on cell lines lacking this mutation (all others). We also envisaged that masking the carboxylate of **MB710** as a simple ethylamide **MB725** might influence cell permeability and uptake of the scaffold. Interestingly, **60** and **MB710** showed relatively low toxicity against all cell lines tested at concentrations up to 60 μM, while showing initial selective viability reduction at higher concentrations (Fig. 6A–B). In particular, NUGC3 was the most sensitive cell line. Strikingly, **MB725** induced strong and selective cell viability reduction in Y220C cell lines NUGC3 (cell viability = 10%), HUH-7 (cell viability = 30%), and BXPC-3 (cell viability = 30%) at concentrations below 40 μM, while showing very low toxicity in other cell lines (cell viability > 80%). An almost maximum effect was achieved at concentrations below 10 μM in NUGC3 cells (Fig. 6C). In contrast, **1**–**3** did not show notable selectivity among the different cell lines (Fig. 6D–E). In particular, **3** displayed high and unspecific toxicity against all cell lines.

**Table 2:** IC_50_ values (μM) for cancer cell lines treated with **60**, PK083 (**1**), MB710 (**70**), PK7088 (**2**), MB725 (**72**), and PK5196 (**3**) for 72 h. Y220C cell lines are underlined.

compound	NUGC3	NUGC4	WI38	BXPC3	SW1088	HUH7	HUH6
**60**	102	159	ND	ND	ND	150^a^	>150
**1**	14	9	22	43	ND	ND	ND
**2**	43	>120	120^a^	57	ND	ND	ND
**3**	6	9	7	11	ND	ND	ND
**70**	90	120^a^	>120	ND	>120	ND	ND
**72**	10	>120	>120	18	>120	10	ND

a Estimate assuming complete cell death at higher concentrations.

**Fig. 7:**
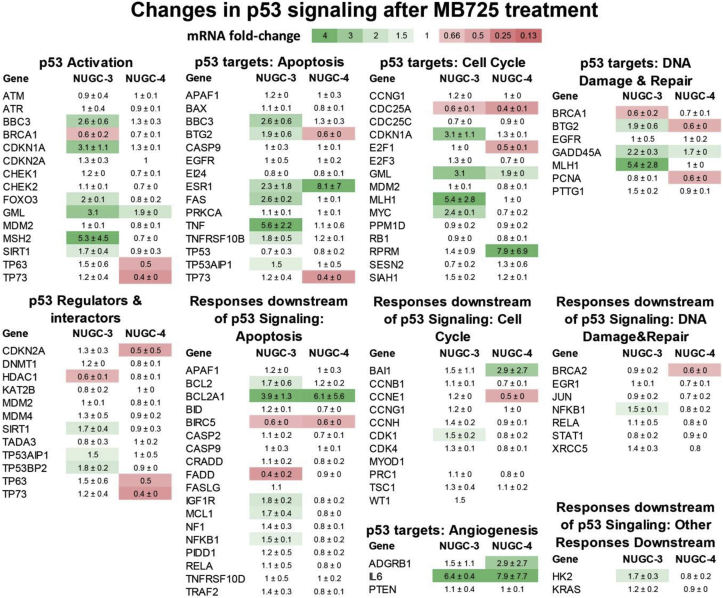
Heatmap of mRNA fold changes in p53 signaling after treatment with 60 μM **MB725** for 18 h in comparison to control. The qPCR array comprised 84 genes related to p53-mediated signal transduction, classified into subgroups for p53 activation and regulation, p53-mediated apoptosis, cell cycle arrest, DNA damage repair, and respective downstream responses [47]. Changes in mRNA levels were calculated using the ΔΔCt method. A value of 1 indicates no change in relative transcript levels between control and **MB725** treated samples (values between 0.66 and 1.5 are shown in white). Increased mRNA levels are shown in green, starting from 1.5 (light green) to 4 (dark green), and decreased mRNA levels are shown in red, ranging from 0.66 (light red) to 0 (dark red). For each gene the average fold-change of two measurements (independent biological replicates) with standard deviation is shown. For *GML*, *TP63*, *TP53AIP1*, *FASLG*, *MYOD1*, *GML*, *WT1*, and *XRCC5* only one or no ΔΔCt values could be obtained. Especially p53-target genes that are involved in apoptotic signaling (e.g., *PUMA* (*BBC3*), *FAS*, *TNF*, *FOXO3*, *BTG2*) and cell cycle modulation (*p21* (*CDKN1A*), *MYC*, *MLH1*) were selectively upregulated in NUGC3 (p53-Y220C) cells after **MB725** treatment, suggesting Y220C-dependent induction of apoptosis and cell cycle arrest in this cell line.

To check whether the compound-induced cell viability reduction in p53-Y220C cell lines is mediated by increased transcriptional activity of p53, we determined relative changes in mRNA levels for 84 genes related to p53-mediated signal transduction via real-time PCR (Fig. 7). After treatment with **MB725** (60 μM) for 18 h, several proapoptotic p53 target genes including *PUMA* (*BBC3*), *BTG2*, *FAS*, *TNF* as well as *p21* (*CDKN1A*), which promotes cell cycle arrest, were selectively upregulated in NUGC3 cells (p53-Y220C). In contrast, **MB725** induced only one proapototic p53 target gene, estrogen receptor 1 (ESR1), in NUGC4 (p53 WT) cells, which is in good agreement with low cell viability reduction in this cell line (Fig. 6). Apart from changes in cell cycle arrest and apoptosis signaling, we observed mild activation of the DNA damage response gene GADD45A and upregulation of angiogenesis promoting Interleukin-6 (IL6) in both cell lines, as well as upregulation of the glycolytic enzyme hexokinase 2 (HK2) and the DNA mismatch repair protein MLH1 in NUGC3 cells.

Several well-known p53 target genes, such as *PUMA* (*BBC3*), *p21* (*CDKN1A*), *BTG2*, *FAS*, and *TNFRSF10B* that are known to be transcribed by p53 in more than 6 independent genome-wide studies [44], and additional p53 target genes, such as the proapoptotic transcription factor *FOXO3*, the proinflammatory and apoptosis promoting tumor necrosis factor (*TNF*), the tumor protein p53 regulated apoptosis inducing protein (*TP53AIP*), and the DNA mismatch repair protein MLH1, were selectively upregulated in NUGC3 cells upon treatment with our compound. These results are in line with the proposed stabilization and transcriptional activation of p53-Y220C by **MB725** in NUGC3 cells. Transcript levels of other well-known p53 target genes, like *MDM2*, *BAX*, and *PPM1D*, did not increase after compound treatment. However, transcription of p53 target genes depends not only on p53 stability and DNA-binding capability, but is also modulated by post-translational modifications and cellular binding partners as well as the p53 response elements themselves, leading to transcription and repression of specific subsets of p53 target genes [45].

**Fig. 8:**
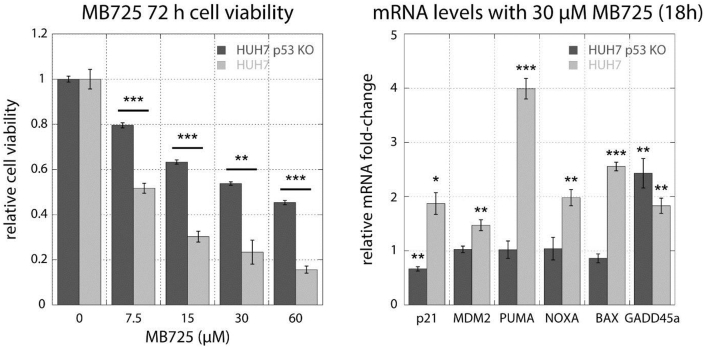
Assessment of p53-Y220C dependent effect of **MB725**. Treatment of HUH-7 (p53-Y220C) and HUH-7 p53-Y220C KO cell lines with **MB725** for 72 h underscores the enhanced cytotoxicity of **MB725** in the presence of p53-Y220C (left panel). Additionally, p53-target genes were more potently upregulated in the p53-Y220C containing HUH-7 cell line than in the HUH-7 p53-Y220C KO cell line (right panel). These results demonstrate that the anticancer activity of **MB725** depends at least partially on p53-Y220C. Cell viability was measured in quadruplicate and normalized against the values of blank (viability = 1) and no cell (viability = 0) controls (left panel). Data are shown as mean ± SEM (Unpaired *t*-test to test for significance in HUH7 and HUH7 p53 KO viability reduction; *p < 0.05; **p < 0.01; ***p < 0.001). Relative mRNA fold-change was measured in triplicate and normalized against untreated sample according to the ΔΔCt method. Data are shown as mean ± SEM (Unpaired *t*-test to test for significance in compound mediated mRNA level changes; *p < 0.05; **p < 0.01; ***p < 0.001).

We also observed Y220C-independent effects of **MB725**: Some p53 target genes were upregulated (*GADD45A*, *GML*, *ESR1*) or repressed (*BIRC5*, *CDC25A*) in both NUGC3 and NUGC4 cells. There were also increased transcript levels in both cell lines for interleukin-6 (*IL6*), which promotes anti-tumor adaptive immunity but also supports cancer cell proliferation, survival, and angiogenesis [46], and the apoptosis suppressing BCL2 Related Protein A1 (*BCL2A1*), as well as repression of p63, p73, and *E2F1* levels in NUGC4. To confirm that **MB725** mediated anticancer effects are dependent on p53-Y220C, we additionally tested the compound in HUH-7 and an in-house CRISPR generated isogenic p53-Y220C knock-out (KO) HUH-7 cell line (Fig. 8). This new cell line contains a frameshift mutation at codon 124 on one allele and deletion of amino acids 125–223 on the other allele, leading to functional inactivation of the p53 DNA-binding domain (aa 92–292). **MB725** decreased cell viability by ca. 30–40% more potently in HUH-7 than in the isogenic HUH-7 p53-Y220C KO (Fig. 8, upper panel). Following a similar pattern, **MB710** also showed stronger viability reduction in HUH-7 cells than in HUH-7 p53-Y220C KO cells, although at higher compound concentration (Fig. S9). Consistently, **MB725** treatment increased mRNA levels of a range of p53 target genes, notably *PUMA* (*BBC3*), *p21*, *MDM2*, NOXA and *BAX*, more efficiently in HUH-7 than in HUH-7 p53-Y220C KO (Fig. 8, lower panel). Taken together, our data show that **MB725** selectively reduces cell viability in p53-Y220C cell lines and upregulates transcription of specific p53 target genes associated with apoptosis and cell cycle arrest in a p53-Y220C dependent manner. These results are in line with the proposed chaperone-mediated restoration of p53-Y220C transcriptional activity.

## 3 Conclusions

We have developed a novel class of small molecules aimed at stabilizing the folded form of the p53 cancer mutant Y220C and reactivating its tumor suppressor function in cancer. Synthetic optimization led to the development of the highly substituted aminobenzothiazole derivative **MB710** that exhibited a 200-fold affinity increase compared with the starting fragment hit **4**. We determined the binding mode of **MB710** and related compounds by X-ray crystallography, highlighting several key structural features mediating its potency, such as (1) the pyrrole moiety targeting the transiently open subsite 3, (2) the benzothiazole scaffold that connects the central cavity and subsite 2 via a conformationally restricted, aromatic sulphur heterocycle, and (3) the *N*-ethyl group that targets a hydrophobic hotspot in subsite 2 formed by Pro151, Pro152, Pro153 and Thr155. Importantly, we showed that **MB725**, an ethylamide derivative of **MB710**, induced selective viability reduction in several cancer cell lines containing the oncogenic p53-Y220C mutation but was well tolerated in other cell lines. In the gastric cancer cell line NUGC3, this viability reduction correlated with increased transcription of several genes involved in apoptotic and cell cycle arrest signaling. **MB725** increased p53 target genes *BTG2*, *p21*, *PUMA*, *FAS*, *TNF*, *TNFRSF10B* in NUGC3, but not in p53 WT NUGC4 cells. Furthermore, **MB725** showed enhanced viability reduction and upregulated p53 target gene transcription in HUH-7 cells while showing significantly reduced effect in a CRISPR generated isogenic p53-Y220C KO cell line, suggesting compound-mediated transcriptional activation of the Y220C mutant. Taken together, our chemical probe **MB725** displays selective targeting of Y220C cancer cells, and the correlation between *in vitro* thermal stabilization and selective viability reduction in Y220C cell lines represents an important milestone towards first-in-class anticancer drugs that rescue p53-Y220C function. Off-target effect and specificity are important considerations at all stages of the drug discovery process. While still at an early stage, we paid particular attention to avoiding reactive functionalities (e.g. alkylators, redox) and other structural features usually found in pan assay interference (PAIN) compounds to minimize toxicity and specificity issues. Baell and co-workers recently provided a good overview of known and suspected PAINs [42], although the definition of PAINs is still the subject of intense debates [48]. Overall, the data presented here provide a blueprint for the development of potent, non-toxic compounds that reactivate the p53 Y220C mutant in tumor cells.

## 4 Materials and methods

### 4.1 Protein expression and purification

Stabilized p53-Y220C DBD (residues 94–312) was expressed and purified as previously described [32,49,50]. Briefly, the N-terminal fusion protein (6xHis/lipoyl domain/TEV protease cleavage site) was overexpressed using *E. coli* C41 cells in 2xTY medium at 20 °C for 16 h and purified using standard Ni-affinity chromatography protocols. After overnight digestion with TEV protease, the p53-Y220C DBD was further purified using a Heparin column. Finally, gel filtration chromatography was performed using a Superdex 75 16/60 preparative gel filtration column (GE Healthcare) in a 25 mM KPi (pH 7.2), 150 mM NaCl, and 1 mM TCEP buffer. Molecular weight and protein purity (>95%) were confirmed via SDS gel electrophoresis and ESI–MS.

### 4.2 Docking

The available crystal structure of **9** was prepared using the Protein Preparation Wizard [51] from Schrodinger, and the corresponding grids were generated with Glide [[35], [36], [37], [38]]. Ligands were prepared (Ligprep [52]) and docked (Glide) in the grid. No constraint was applied to the system. Docking poses were subjected to one round of Prime [53] minimization, then analyzed visually with Maestro [35] and Pymol (www.pymol.org).

### 4.3 Differential scanning fluorimetry

DSF was performed as described [32]. Briefly, DSF measurements were performed using 8 μM protein (stabilized p53-Y220C DBD) and 10 × SYPRO orange (Life Technologies) in a 25 mM KPi (pH 7.2), 150 mM NaCl, and 1 mM TCEP assay buffer at a final DMSO concentration of 5% (v/v). Δ*T*_m_ values were calculated as Δ*T*_m_ = *T*_m_ (protein + compound) − *T*_m_ (protein). All samples were measured in triplicate.

### 4.4 Isothermal titration calorimetry (ITC)

Isothermal Titration Calorimetry (ITC). ITC experiments were conducted as described [28]. The cell unit contained 50 μM protein in a 25 mM KPi (pH 7.2), 150 mM NaCl, 1 mM TCEP, and 5% (v/v) DMSO assay buffer. The syringe contained 2–5 mM compound in the same buffer. For **MB710** a reverse titration was performed in the same assay buffer using 190 μM protein for the syringe and 15 μM compound for the cell.

### 4.5 HSQC-NMR

^1^H/^15^N-HSQC spectra of uniformly ^15^N-labeled T-p53C-Y220C (75 μM) and compounds were recorded and analyzed as described [28]. Spectra were acquired at 293 K on a Bruker Avance-800 spectrometer using a 5-mm inverse cryogenic probe. Compound stock solutions were mixed with protein immediately before the NMR measurement. Spectra analysis was performed using Sparky 3.11430 and Bruker Topspin 2.0 software.

### 4.6 Structure determination of Y220C-ligand complexes

Crystals of a stabilized variant of the p53 mutant Y220C were grown at 18 °C using the sitting drop vapor diffusion technique as described [26]. They were soaked for 3–4 h in a 30–40 mM solution of compound in cryo buffer (19% polyethylene glycol 4000, 20% glycerol, 10 mM sodium phosphate, pH 7.2, 100 mM Hepes, pH 7.2, 150 mM NaCl) and flash frozen in liquid nitrogen. In case of insufficient compound solubility, saturated solutions were used for soaking. X-ray data sets were collected at 100 K at beamlines I02, I03, and I04 of the Diamond Light Source, Oxford, UK. The data sets were indexed using XDS [54] and scaled using the program SCALA [55] within the CCP4 software suite [56]. After an initial rigid-body refinement in PHENIX [57] with PDB entry 2J1X as a staring model, the structures of the Y220C-ligand complexes were refined using iterative cycles of manual model building in COOT [58] and refinement with PHENIX. In the Y220C-**MB710** complex, there was only partial occupancy of the ligand. Therefore, alternate states of the binding pocket were refined, i.e., pocket with bound **MB710** and apo-structure with its characteristic water network (modelled in chain A only). In the structure with **11** and **13**, there was significant additional electron density extending from the iodine atom that is not involved in halogen bonding into subsite 2, combined with negative difference density at the iodine, suggesting partial breakage of the carbon-iodine bond as a result of radiation damage during data collection. Similar observations had been made upon determining the structure of the parent compound **9**. X-ray data collection and refinement statistics are given in Table S2. Structural figures were prepared using PyMOL (www.pymol.org).

### 4.7 Cell culture and cell viability assays

WI-38 and BXPC-3 (p53-Y220C) cell lines were purchased from ATCC and HUH-7 (p53-Y220C+/+), HUH-6 (wild-type p53+/+). NUGC3 (p53-Y220C+/+), and NUGC4 (wild-type p53) cells were obtained from the Japan Health Science Research Resources Bank. The p53-Y220C knock-out (KO) HUH-7 cell line was generated from the original HUH-7 cell line using CRISPR gene editing [59]. The HUH-7 p53 KO cell line contains on one allele a frameshift mutation at codon 124 and on the other allele the DNA sequence encoding amino acids 125–223 is deleted, leading to functional inactivation of the p53 DNA-binding domain (aa 92–292). A detailed description of the method and the HUH-7 p53 KO cell line will be presented elsewhere (Bauer, Jones et al., 2018, manuscript in preparation). All cell lines were cultured as previously described [9]. Cell viability was measured in quadruplicate (technical replicates) using the CellTiter-Fluor or CellTiter-Glo 2.0 cell viability assay kits (Promega, USA) as previously described [9]. Cell viability results for each compound were confirmed in at least 2 independent biological replicates.

### 4.8 p53 signaling qPCR array

Cells were treated in 6-well plates for 18 h with a DMSO content of 0.5% for both compound and control samples. Total RNA was extracted and purified using RNeasy Mini Kit (Qiagen) according to the manufacturer's instructions. Synthesis of cDNA was performed with a RT^2^ First Strand Kit (Qiagen) using 800 ng RNA per reaction. After pipetting RT [2] SYBR Green ROX FAST Mastermix (Qiagen) and cDNA (22.5× dilution of cDNA synthesis reaction as final concentration) into a Rotor-disc ring containing the RT^2^ Profiler PCR Array Human p53 Signaling Pathway (Qiagen), real-time PCR was performed according to the manufacturer's instructions using the Rotor-Gene 6000 (Corbett Life Science) PCR cycler. mRNA fold-changes were quantified with the ΔΔCt method using β2-Microglobulin (B2M) as housekeeping gene. For each cell line, two independent biological replicates were measured with the qPCR array.

### 4.9 Real-time PCR

Real-time PCR experiments were performed as described [9]. Briefly, cells were treated in 6-well plates for 18 h with a DMSO content of 0.5% for both compound and control samples. The ΔΔCt method was used to quantify relative mRNA levels. Each sample was measured in triplicate.

## Accession codes

PDB ID codes for the X-ray structures of Y220C in complex with **11**, **13**, **34**, **50**–**52**, **55**, **60**, **70** have been deposited in the Protein Data Bank (PDB) under accession codes 5O1A, 5O1B, 5O1C, 5O1D, 5O1E, 5O1F, 5O1G, 5O1H, and 5O1I, respectively.

## Author contributions

The manuscript was written with contributions from all authors. All authors have given approval to the final version of the manuscript.

## Conflicts of interest

There are no conflicts to declare.

## Abbreviations

DBD, DNA-binding domain; DSF, differential scanning fluorimetry; HSQC, heteronuclear single quantum coherence; ITC, isothermal titration calorimetry; *K*_d_, dissociation constant; LE, ligand efficiency; NMR, nuclear magnetic resonance; PCR, polymerase chain reaction; PDB, protein data bank; quant., quantitative; T_m_, melting temperature; WT, wild-type.

## Appendix A Supplementary data

Material and methods, supplementary figures and tables, synthetic procedures and characterization, SMILES representations of compounds **1**–**72**. This material is available free of charge via the Internet at http://pubs.acs.org.
