# Supplementary material for: Aminobenzothiazole derivatives stabilize the thermolabile p53 cancer mutant Y220C and show anticancer activity in p53-Y220C cell lines
Source: Eur J Med Chem. 2018 May 25;152:101–14. doi: 10.1016/j.ejmech.2018.04.035 (PMC5986712; doi:10.1016/j.ejmech.2018.04.035)
Supplement: Supplementary data 2 [file mmc2.docx]

**Supplementary Information**

Aminobenzothiazole Derivatives Stabilize the Thermolabile p53 Cancer Mutant Y220C and Show Anticancer Activity in p53-Y220C Cell Lines

Matthias G. J. Baud,*^a,b,§^ Matthias R. Bauer,^a,§^ Lorena Verduci,^a^ Felix A. Dingler,^a^ Ketan J. Patel,^a^ Deeptee Horil Roy,^a^ Andreas C. Joerger,^a,c^ and Alan R. Fersht*^a^

*Correspondence to: [m.baud@soton.ac.uk](mailto:m.baud@soton.ac.uk), [alan@mrc-lmb.cam.ac.uk](mailto:alan@mrc-lmb.cam.ac.uk)

^a^Medical Research Council, Laboratory of Molecular Biology, Francis Crick Avenue, Cambridge CB2 0QH, United Kingdom

^b^Chemistry, Faculty of Natural and Environmental Sciences, University of Southampton, Southampton SO17 1BJ, UK.

^c^German Cancer Consortium (DKTK), German Cancer Center (DKFZ), 69120 Heidelberg, and Institute of Pharmaceutical Chemistry, Johann Wolfgang Goethe University, Max-von-Laue-Str. 9, 60438 Frankfurt am Main, Germany.

§These authors contributed equally

Table of content:

Synthetic procedures

Figures S1-S9

Tables S1-S3

**Synthetic procedures**

All reagents and solvents were obtained from commercial sources, and used as supplied unless otherwise indicated. Reactions requiring anhydrous conditions were conducted in heated glassware (heat gun), under an inert atmosphere (argon), and using anhydrous solvents. All reactions were monitored by analytical thin-layer chromatography (TLC) using indicated solvent systems on E. Merck silica gel 60 F254 plates (0.25 mm). TLC plates were visualized using UV light (254nm) and/or by staining in potassium permanganate followed by heating. Solvents were removed by rotary evaporator below 40˚C and the compounds further dried using high vacuum pumps.

Liquid chromatography–mass spectrometry (LCMS) analyses were performed with an Agilent HPLC 1100 series connected to a Bruker Daltonics MicroTOF and a diode array detector. Purity of all compounds was evaluated on a Waters X--‐bridge C18 column (50 mm x 2.1 mm, 3.5 mm particle size), with a mobile phase of water/acetonitrile+0.1% TFA, using a linear gradient from 80:20 to 5:95 over 3.5 min and then held for 1.5 min, at a flow rate of 0.5 mL/min. Purity of compounds was >95% as measured by peak area ratio.

^1^H and ^13^C NMR were recorded on a Bruker Advance 400 spectrophotometer at 400 MHz and 100 MHz respectively. Chemical shifts (δ H) are quoted in ppm (parts per million) and referenced to residual solvent signals: ^1^H δ = 7.26 (CDCl_3_), 2.50 (*d^6^*-DMSO), 3.31 (CD_3_OD), ^13^C δ = 77.0 (CDCl_3_), 39.43 (*d^6^*-DMSO), 49.05 (CD_3_OD). Coupling constants (*J*) are given in Hz. High resolution mass spectra (ESI) were recorded on a Waters LCT Premier Mass Spectrometer.

**Subsite 1 SAR**

**Methyl 4-amino-3,5-diiodo-2-methoxybenzoate** **(6)**

To a solution of methyl 4-amino-2-methoxybenzoate **5** (2.00 g, 11.0 mmol, 1 eq.) in MeCN (70 mL) was added *N*-iodosuccinimide (5.22 g, 23.2 mmol, 2.1 eq.) in 2 portions over 5 minutes. The resulting mixture was stirred at rt under argon for 1h, then at 55°C. *N*-iodosuccinimide (1.0 eq.) was added every 5h until completion of the reaction. The reaction mixture was then partitioned between CHCl_3_ and sat. aq. NaHCO_3_. A small amount of sat. aq. Na_2_S_2_O_3_ was added to quench potential traces of iodine. The aqueous phase was extracted 3 times. The combined organic phases were dried (MgSO_4_) and concentrated under reduced pressure. The product (2.10 g, 44%) was obtained after purification by flash column chromatography (hexane/AcOEt 8:2). R_f_ 0.4 (hexane/AcOEt, 8:2); ^1^H NMR (400 MHz, CDCl_3_) δ 3.85 (s, 3H), 3.88 (s, 3H), 5.15 (br. s., 2H), 8.27 (s, 1H); ^13^C NMR (100 MHz, CDCl_3_) δ 52.1, 61.9, 74.0, 81.5, 114.7, 142.4, 151.2, 161.6, 163.7; HRMS (ESI+) m/z calcd for C_9_H_10_I_2_NO_3_ [M + H]^+^ 433.8745, found: 433.8746.

**Methyl 3,5-diiodo-2-methoxy-4-(1H-pyrrol-1-yl)benzoate** **(7)**

To a solution of aniline **6** (1.00 g, 2.31 mmol, 1.0 eq.) in AcOH (10 mL) was added 2,5-dimethoxytetrahydrofuran (330 µL, 2.54 mmol, 1.1 eq.) at rt, and the resulting mixture was stirred at 80°C for 4h. The volatiles were removed *in vacuo*. The residue was partitioned between CHCl_3_ and sat. aq. NaHCO_3_, and the aqueous phase was extracted twice. The combined organic phases were dried (MgSO_4_) and concentrated under reduced pressure. The product (1.12 g, quant.) was obtained as a light yellow solid after purification by flash column chromatography (hexane/AcOEt 8:2). R_f_ 0.6 (hexane/AcOEt, 8:2); ^1^H NMR (400 MHz, CDCl_3_) δ 3.93 (s, 3H), 3.96 (s, 3H), 6.39 (m, 2H), 6.60 (m, 2H), 8.31 (s, 1H); ^13^C NMR (100 MHz, CDCl3) δ 52.9, 62.5, 91.0, 100.0, 109.9, 120.4, 126.5, 141.3, 150.4, 160.6, 163.6; HRMS (ESI+) m/z calcd for C_13_H_12_I_2_NO_3_ [M + H]^+^ 483.8901, found: 483.8912.

**Methyl 2-hydroxy-3,5-diiodo-4-(1H-pyrrol-1-yl)benzoate** **(8)**

To a 0°C solution of **7** (900 mg, 1.86 mmol, 1.0 eq.) in anhydrous CH_2_Cl_2_ (9 mL) under argon was added BBr_3_ (0.5 M in CH_2_Cl_2_, 4.46 mL, 2.23 mmol, 1.2 eq.) dropwise over 10 minutes. The resulting solution was stirred at 0°C for 1h, then quenched with a small amount of MeOH, and partitioned between CH_2_Cl_2_ and sat. aq. NaHCO_3_. The aqueous phase was extracted twice. The combined organic layers were dried (MgSO_4_) and concentrated under reduced pressure. The product (780 mg, 89%) was obtained as a light yellow solid after purification by flash column chromatography (hexane/CH_2_Cl_2_ 1:1). R_f_ 0.25 (hexane/CH_2_Cl_2_, 6:4); ^1^H NMR (400 MHz, DMSO-*d_6_*) δ 3.96 (s, 3H), 6.25 (m, 2H), 6.68 (m, 2H), 8.25 (s, 1H), 11.44 (s, 1H); ^13^C NMR (100 MHz, DMSO-*d_6_*) δ 53.4, 85.6, 92.8, 109.4, 114.4, 120.4, 138.8, 151.2, 160.0, 167.3; HRMS (ESI+) m/z calcd for C_12_H_10_I_2_NO_3_ [M + H]^+^ 469.8745, found: 469.8743.

**6-(hydroxymethyl)-2,4-diiodo-3-(1H-pyrrol-1-yl)phenol** **(13)**

To a solution of ester **8** (350 mg, 0.746 mmol, 1.0 eq.) in a 2.5:1 CH_2_Cl_2_/MeOH mixture (9.8 mL) and under argon, was added NaBH_4_ (56 mg, 1.49 mmol, 2 eq.). The reaction was followed by LCMS and 2.0 eq. NaBH_4_ were added every hour until the reaction was complete. Following completion, thge reaction mixture was partitioned between CH_2_Cl_2_ and H_2_O. The pH of the aqueous phase was adjusted to ca. 2-3 by addition of 1N HCl, followed by extraction with CH_2_Cl_2_ (3X). The combined organic phases were dried (MgSO_4_) and concentrated under reduced pressure. The product (173 mg, 53%) was obtained as a white solid after purification by flash column chromatography (100% CH_2_Cl_2_). R_f_ 0.75 (CH_2_Cl_2_/AcOEt, 7:3); ^1^H NMR (400 MHz, DMSO-*d_6_*) δ 4.56 (s, 2H), 5.55 (br. s., 1H), 6.20 (m, 2H), 6.60 (m, 2H), 7.77 (s, 1H), 9.63 (br. s., 1H); ^13^C NMR (100 MHz, DMSO-*d_6_*) δ 58.7, 85.9, 92.7, 108.9, 120.9, 121.2, 131.9, 136.2, 144.5, 154.6; HRMS (ESI+) m/z calcd for C_11_H_10_I_2_NO_2_ [M + H]^+^ 441.8795, found: 441.8788.

**2-hydroxy-3,5-diiodo-*N*-methyl-4-(1H-pyrrol-1-yl)benzamide** **(12)**

To a suspension of ester **8** (50 mg, 0.107 mmol, 1.0 eq.) in MeOH (1 mL) was added methylamine (40% aq., 1mL) at rt. The resulting mixture was stirred at rt for 3h, then concentrated to dryness. The product (42 mg, 84%) was obtained as a white solid after purification by flash column chromatography (CH_2_Cl_2_/MeOH 98:2). R_f_ 0.6 (CH_2_Cl_2_/MeOH, 98:2); ^1^H NMR (400 MHz, DMSO-*d_6_*) δ 2.85 (d, *J* = 4.4 Hz, 3H), 6.24 (m, 2H), 6.67 (m, 2H), 8.42 (s, 1H), 9.27 (br. m, 1H), 14.4 (s, 1H); ^13^C NMR (100 MHz, DMSO-*d_6_*) δ 26.3, 84.3, 92.6, 109.2, 115.6, 120.5, 136.1, 149.6, 161.3, 168.0; HRMS (ESI+) m/z calcd for C_12_H_11_I_2_N_2_O_2_ [M + H]^+^ 468.8904, found: 468.8911.

**2-hydroxy-3,5-diiodo-4-(1H-pyrrol-1-yl)benzoic acid** **(9)**

To a solution of ester **8** (200 mg, 0.426 mmol, 1.0 eq.) in THF (2 mL) was added 4N aq. NaOH (2 mL). The resulting mixture was stirred vigorously at rt and the reaction was followed by LCMS. After completion of the reaction, the mixture was partitioned between CHCl_3_ and H_2_O. The pH of the aqueous phase was adjusted to ca. 1-2. The aqueous phase was extracted 4 times with CHCl_3_. The combined organic phases were dried (MgSO_4_) and concentrated under reduced pressure. The product (175 mg, 90%) was obtained as a brown solid after purification by flash column chromatography (CH_2_Cl_2_/MeOH 8:2). R_f_ 0.5 (CH_2_Cl_2_/MeOH, 8:2); ^1^H NMR (400 MHz, DMSO-*d_6_*) δ 6.22 (m, 2H), 6.65 (m, 2H), 8.21 (s, 1H); ^13^C NMR (100 MHz, DMSO-*d_6_*) δ 83.0, 92.2, 109.2, 116.7, 120.5, 139.1, 149.9, 162.6, 169.5; HRMS (ESI+) m/z calcd for C_11_H_8_I_2_NO_3_ [M + H]^+^ 455.8588, found: 455.8575.

**2-hydroxy-3,5-diiodo-*N*-methoxy-*N*-methyl-4-(1H-pyrrol-1-yl)benzamide** **(14)**

To a solution of acid **9** (1.70 g, 3.73 mmol, 1.0 eq.) in anhydrous DMF (17 mL) were successively added HOBt.H_2_O (733 mg, 4.85 mmol, 1.3 eq.) and EDCI (787 mg, 4.10 mmol, 1.1 eq.). After stirring for 10 minutes at rt, *N*,*O*-dimethylhydroxylamine hydrochloride (400 mg, 4.10 mmol, 1.1 eq.) and diisopropylethylamine (287 µL, 1.65 mmol, 1.5 eq.) were added. After stirring at rt for 6h, the reaction mixture was diluted with brine and extracted with CHCl_3_. The organic layer was dried (MgSO_4_) and concentrated under reduced pressure. The product (1.00 g, 54%) was obtained as a white solid after purification by flash column chromatography (100% CH_2_Cl_2_). R_f_ 0.4 (100% CH_2_Cl_2_); ^1^H NMR (400 MHz, CDCl_3_) δ 3.45 (s, 3H), 3.73 (s, 3H), 6.40 (m, 2H), 6.62 (m, 2H), 8.56 (s, 1H), 12.4 (s, 1H); ^13^C NMR (100 MHz, CDCl_3_) δ 34.0, 62.7, 83.2, 91.5, 109.7, 115.6, 120.4, 139.3, 149.9, 161.2, 166.9; HRMS (ESI+) m/z calcd for C_13_H_13_I_2_N_2_O_3_ [M + H]^+^ 498.9010, found: 498.9019.

**N,2-dihydroxy-3,5-diiodo-4-(1H-pyrrol-1-yl)benzamide (15)**

To a solution of **14** (100 mg, 0.201 mmol, 1.0 eq.) in THF (2 mL) was added hydroxylamine (2.88M in H_2_O, 697 µL, 10 eq.). The resulting mixture was stirred at rt for 1.5h, then partitioned between AcOEt and 1N HCl. The aqueous phase was extracted 4 times. The combined organic layers were dried (MgSO_4_) and concentrated under reduced pressure. The product (80 mg, 85%) was obtained after purification by flash column chromatography (CH_2_Cl_2_/MeOH/AcOH 90:10:0.5). R_f_ 0.35 (CH_2_Cl_2_/MeOH, 9:1); ^1^H NMR (400 MHz, DMSO-*d_6_*) δ 6.24 (m, 2H), 6.65 (m, 2H), 8.27 (s, 1H), 9.70 (br. s, 1H), 12.10 (br. S, 1H), 13.91 (br. S, 1H); ^13^C NMR (100 MHz, DMSO-*d_6_*) δ 83.9, 93.0, 109.2, 114.5, 120.5, 135.4, 149.3, 161.0, 164.3; HRMS (ESI+) m/z calcd for C_11_H_9_I_2_N_2_O_3_ [M + H]^+^ 470.8697, found: 470.8713.

**2-hydroxy-3,5-diiodo-4-(1H-pyrrol-1-yl)benzaldehyde** **(16)**

To a -78°C solution of **14** (200 mg, 0.402 mmol, 1.0 eq.) in anhydrous THF (5 mL) and under argon was added DIBAL-H (1M in heptane, 442 µL, 0.442 mmol, 1.1 eq.) dropwise. After 5 minutes at -78°C, an additional 1.1 eq. DIBAL-H was added dropwise, and the reaction was left at -78°C for 1h. The temperature was then allowed to reach rt gradually over a few hours. The reaction was quenched with H_2_O. The reaction mixture was partitioned between CH_2_Cl_2_ and 1N HCl. The aqueous phase was extracted twice. The combined organic layers were dried (MgSO_4_) and concentrated under reduced pressure. The product (96 mg, 54%) was obtained as a light yellow solid after purification by flash column chromatography (hexane/AcOEt 8:2). R_f_ 0.3 (hexane/AcOEt, 8:2); ^1^H NMR (400 MHz, CDCl_3_) δ 6.16 (m, 2H), 6.36 (m, 2H), 7.82 (s, 1H), 9.56 (s, 1H), 11.70 (s, 1H); ^13^C NMR (100 MHz, CDCl_3_) δ 84.5, 91.5, 109.3, 110.2, 120.2, 143.3, 152.5, 161.6, 194.3; HRMS (ESI+) m/z calcd for C_11_H_8_I_2_NO_2_ [M + H]^+^ 439.8639, found: 439.8611.

**6-((dimethylamino)methyl)-2,4-diiodo-3-(1H-pyrrol-1-yl)phenol** **(17)**

To a solution of aldehyde **16** (60 mg, 0.137 mmol, 1.0 eq.) in CH_2_Cl_2_ (1.3 mL) were successively added AcOH (7.8 µL, 0.137 mmol, 1 eq.), dimethylamine (2M in THF, 205 µL, 0.410 mmol, 3 eq.) and NaB(OAc)_3_ (58 mg, 0.274 mmol, 2 eq.). The resulting mixture was stirred at rt for 24h, then partitioned between CHCl_3_ and sat. aq. NaHCO_3_. The aqueous phase was extracted 4 times. The combined organic layers were dried (MgSO_4_) and concentrated under reduced pressure. The product (43 mg, 67%) was obtained as a white solid after purification by flash column chromatography (CH_2_Cl_2_/MeOH 98:2). R_f_ 0.5 (CH_2_Cl_2_/MeOH, 96:4); ^1^H NMR (400 MHz, CDCl_3_) δ 2.40 (s, 6H), 3.68 (s, 2H), 6.37 (m, 2H), 6.61 (m, 2H), 7.44 (s, 1H), 10.05 (br. s, 1H); ^13^C NMR (100 MHz, CDCl_3_) δ 44.2, 61.5, 83.1, 89.9, 109.2, 120.8, 123.6, 137.4, 145.8, 159.2; HRMS (ESI+) m/z calcd for C_13_H_15_I_2_N_2_O [M + H]^+^ 468.9268, found: 468.9260.

***N*-(2-(dimethylamino)ethyl)-2-hydroxy-3,5-diiodo-4-(1H-pyrrol-1-yl)benzamide** **(10)**

To a solution of acid **9** (200 mg, 0.440 mmol, 1.0 eq.) in anhydrous CHCl_3_ (3 mL) was successively added HOBt.H_2_O (86 mg, 0.571 mmol, 1.3 eq.) and EDCI (93 mg, 0.484 mmol, 1.1 eq.) at rt. After stirring for 5 minutes at rt, *N*1,*N*1-dimethylethane-1,2-diamine (58 µL, 0.528 mmol, 1.2 eq.) and Et_3_N (74 µL, 0.528 mmol, 1.2 eq.) were added. The reaction mixture was stirred overnight, then partitioned between CHCl_3_ and 1N NaOH. The aqueous phase was extracted 4 times with CHCl_3_/*i*-PrOH mixture (ca. 4:1). The combined organic layers were dried (MgSO_4_) and concentrated under reduced pressure. The product (60 mg, 26%) was obtained as a white solid after purification by flash column chromatography (CH_2_Cl_2_/MeOH 75:25). R_f_ 0.25 (CH_2_Cl_2_/MeOH, 75:25); ^1^H NMR (400 MHz, DMSO-*d_6_*) δ 2.68 (s, 6H), 3.04 (t, *J* = 6.0 Hz, 2H), 3.57 (*app.* q., J = 6.0 Hz, 2H), 6.14 (m, 2H), 6.54 (m, 2H), 8.12 (s, 1H), 11.75 (br. t, 1H); ^13^C NMR (100 MHz, DMSO-*d_6_*) δ 34.7, 43.4, 57.4, 71.2, 100.9, 108.4, 118.3, 120.5, 138.1, 147.4, 167.0, 168.0; HRMS (ESI+) m/z calcd for C_15_H_18_I_2_N_3_O_2_ [M + H]^+^ 525.9483, found: 525.9501.

**(2-hydroxy-3,5-diiodo-4-(1H-pyrrol-1-yl)phenyl)(4-methylpiperazin-1-yl)methanone** **(11)**

To a solution of acid **9** (400 mg, 0.879 mmol, 1.0 eq.) in anhydrous CHCl_3_ (6 mL) was successively added *N*-hydroxysuccinimide (122 mg, 1.05 mmol, 1.2 eq.) and EDCI (186 mg, 0.967 mmol, 1.1 eq.) at rt. After stirring for 4h at rt, 1-methylpiperazine (244 µL, 2.20 mmol, 2.5 eq.) was added. The reaction mixture was stirred for 2 days, then concentrated to dryness. The product (97 mg, 21%) was obtained as a white solid after purification by flash column chromatography (CH_2_Cl_2_/MeOH/aq. NH_3_ 75:25:0.5). R_f_ 0.4 (CH_2_Cl_2_/MeOH, 9:1); ^1^H NMR (400 MHz, DMSO-*d_6_*) δ 2.25 (s, 3H), 2.41 (br. m, 4H), 3.50 (m, 4H), 6.21 (m, 2H), 6.64 (m, 2H), 7.61 (s, 1H); ^13^C NMR (100 MHz, DMSO-*d_6_*) δ 45.3, 54.0, 55.0, 85.2, 94.6, 109.0, 120.6, 126.2, 136.5, 146.9, 154.4, 154.4; HRMS (ESI+) m/z calcd for C_16_H_18_I_2_N_3_O_2_ [M + H]^+^ 537.9483, found: 537.9455.

**Methyl 4-amino-2-fluoro-3,5-diiodobenzoate (19)**

To a solution of methyl 4-amino-2-fluorobenzoate **18** (1.5 g, 8.87 mmol, 1.0 eq.) in AcOH (45 mL) was added *N*-iodosuccinimide (4.39 g, 19.5 mmol, 2.2 eq.). The resulting mixture was stirred overnight at rt, then concentrated under reduced pressure. The residue was dissolved in AcOEt. The organic phase was washed with sat. aq. NaHCO_3_ (2x) and brine (1x), dried (MgSO_4_) and concentrated. The product (3.70 g, quant.) was used for the next step without further purification. ^1^H NMR (400 MHz, CDCl_3_) δ 3.88 (s, 3H), 5.22 (br. s, 2H), 8.26 (d, *J* = 7.8 Hz, 1H); ^13^C NMR (100 MHz, CDCl_3_) δ 52.2, 69.9 (d, *J* = 32 Hz), 73.3 (d, *J* = 3 Hz), 109.4 (d, *J* = 14 Hz), 142.0 (d, *J* = 2 Hz), 151.4 (d, *J* = 6 Hz), 161.9 (d, *J* = 257 Hz), 162.8 (d, *J* = 6 Hz); HRMS (ESI+) m/z calcd for C_8_H_7_FI_2_NO_2_ [M + H]^+^ 421.8545, found: 421.8553.

**Methyl 2-fluoro-3,5-diiodo-4-(1H-pyrrol-1-yl)benzoate (20)**

To a solution of aniline **19** (2.00 g, 4.75 mmol, 1.0 eq.) in AcOH (30 mL) was added 2,5-dimethoxytetrahydrofuran (739 µL, 5.70 mmol, 1.2 eq.) at rt, and the resulting mixture was stirred at 80°C for 5h. The volatiles were removed *in vacuo*. The residue was partitioned between CHCl_3_ and sat. aq. NaHCO_3_, and the aqueous phase was extracted twice. The combined organic phases were dried (MgSO_4_) and concentrated under reduced pressure. The product (2.34 g, quant.) was used for the next step without further purification. R_f_ 0.7 (100% CH_2_Cl_2_); ^1^H NMR (400 MHz, CDCl_3_) δ 3.98 (s, 3H), 6.41 (m, 2H), 6.60 (m, 2H), 8.44 (d, *J* = 7.4 Hz, 1H); ^13^C NMR (100 MHz, CDCl_3_) δ 53.1, 89.3 (d, *J* = 28 Hz), 90.7 (d, *J* = 4 Hz), 110.3, 120.2, 120.3, 141.7, 151 (d, *J* = 3 Hz), 161.1 (d, *J* = 261 Hz), 162.1 (d, *J* = 5 Hz); HRMS (ESI+) m/z calcd for C_12_H_9_FI_2_NO_2_ [M + H]^+^ 471.8701, found: 471.8711.

**5,7-diiodo-6-(1H-pyrrol-1-yl)-1,2-dihydro-3H-indazol-3-one (21)**

To a solution of ester **20** (500 mg, 1.06 mmol, 1.0 eq.) in a 1:1 CH_2_Cl_2_/EtOH mixture (8 mL) was added hydrazine monohydrate (88 µL, 1.80 mmol, 1.7 eq.)and Et_3_N (325 µL, 2.33 mmol, 2.2 eq.). The resulting mixture was heated at 75°C in a sealed vessel for 24h, then concentrated to dryness. The product (246 mg, 51%) was obtained as an off-white solid after purification by flash column chromatography (CH_2_Cl_2_/MeOH 95:5). R_f_ 0.2 (CH_2_Cl_2_:MeOH, 95:5); ^1^H NMR (400 MHz, DMSO-*d_6_*) δ 6.24 (m, 2H), 6.69 (m, 2H), 8.16 (s, 1H), 10.91 (br. s, 1H), 12.02 (br. s, 1H); ^13^C NMR (100 MHz, DMSO-*d_6_*) δ 79.2, 88.9, 109.0, 116.9, 123.5, 135.3, 148.8, 159.5, 166.2; HRMS (ESI+) m/z calcd for C_11_H_8_I_2_N_3_O [M + H]^+^ 451.8751, found: 451.8749.

**Subsite 2 SAR**

**Methyl 4-amino-5-iodo-2-methoxybenzoate** **(22)**

To a 0°C solution of methyl 4-amino-2-methoxybenzoate **5** (3.0 g, 16.6 mmol, 1.0 eq.) in MeCN (100 mL) was added *N*-iodosuccinimide (3.91 g, 17.4 mmol, 1.05 eq.). The resulting mixture was then stirred at rt for 2h. The mixture was then concentrated and partitioned between AcOEt and sat. aq. NaHCO_3_. The organic phase was washed with sat. aq. NaHCO_3_ (2x) and brine (1x), dried (MgSO_4_) and concentrated under reduced pressure. The product (5.05 g, quant.) was used for the next step without further purification. ^1^H NMR (400 MHz, CDCl_3_) δ 3.81 (s, 3H), 3.82 (s, 3H), 4.52 (br. s, 2H), 6.27 (s, 1H), 8.16 (s, 1H); ^13^C NMR (100 MHz, CDCl_3_) δ 51.6, 55.9, 71.5, 97.1, 111.1, 142.8, 151.6, 161.7, 164.8; HRMS (ESI+) m/z calcd for C_9_H_11_INO_3_ [M + H]^+^ 307.9778, found: 307.9794.

**Methyl 4-amino-2-hydroxy-5-iodobenzoate** **(23)**

To a 0°C solution of **22** (3.0 g, 9.77 mmol, 1.0 eq.) in anhydrous CH_2_Cl_2_ (90 mL) was added BBr_3_ (0.5M in CH_2_Cl_2_, 21.5 mL, 10.7 mmol, 1.1 eq.) dropwise. After stirring at 0°C for 3h, the reaction was quenched with sat. aq. NaHCO_3_, and the aqueous phase was extracted twice with CH_2_Cl_2_. The combined organic layers were dried (MgSO_4_) and concentrated under reduced pressure. The product (1.68 g, 59%) was obtained as a white solid after purification by flash column chromatography (100% CH_2_Cl_2_). R_f_ 0.4 (100% CH_2_Cl_2_); ^1^H NMR (400 MHz, CDCl_3_) δ 3.88 (s, 3H), 4.54 (br. s, 2H), 6.26 (s, 1H), 8.07 (s, 1H), 10.7 (s, 1H); ^13^C NMR (100 MHz, CDCl_3_) δ 51.9, 71.6, 100.3, 105.4, 140.6, 152.6, 163.1, 169.2; HRMS (ESI+) m/z calcd for C_8_H_9_INO_3_ [M + H]^+^ 293.9622, found: 293.9619.

**General procedure 1: Suzuki coupling of 23 with arylboronic acids**

A mixture of iodo derivative **23** (1.0 eq.), arylboronic acid (1.1 eq.), Pd(PPh_3_)_4_ (0.05 eq.), Cs_2_CO_3_ (3 eq.) in a degassed dioxane/H_2_O mixture (82:18, 9.9 mL/mmol) was stirred at 80°C for 5h under an argon atmosphere. Most dioxane was then removed under reduced pressure. The residue was partitioned between CHCl_3_ and H_2_O. The pH of the aqueous phase was adjusted to ca. 7-8, followed by extraction with CHCl_3_ (2x). The combined organic layers were dried (MgSO_4_) and concentrated under reduced pressure. The product was obtained after purification by flash column chromatography.

**Methyl 6-amino-4-hydroxy-[1,1'-biphenyl]-3-carboxylate** **(24)**

**24** (265 mg, 80%) was prepared according to general procedure 1 and was obtained after purification by flash column chromatography (hexane/CH_2_Cl_2_ 3:7). R_f_ 0.5 (100% CH_2_Cl_2_); ^1^H NMR (400 MHz, CDCl_3_) δ 3.87 (s, 3H), 4.23 (br. s, 2H), 6.25 (s, 1H), 7.31-7.48 (m, 5H), 7.59 (s, 1H), 10.9 (s, 1H); ^13^C NMR (100 MHz, CDCl_3_) δ 51.7, 100.9, 103.2, 119.9, 127.4, 129.0, 129.2, 132.2, 138.1, 150.6, 162.7, 170.5; HRMS (ESI+) m/z calcd for C_14_H_14_NO_3_ [M + H]^+^ 244.0978, found: 244.0961.

**Methyl 6-amino-4'-fluoro-4-hydroxy-[1,1'-biphenyl]-3-carboxylate** **(25)**

**25** (252 mg, 80%) was prepared according to general procedure 1 and was obtained after purification by flash column chromatography (hexane/CH_2_Cl_2_ 2:8). R_f_ 0.55 (100% CH_2_Cl_2_); ^1^H NMR (400 MHz, CDCl_3_) δ 3.87 (s, 3H), 4.17 (br. s, 2H), 6.25 (s, 1H), 7.12 (m, 2H), 7.35 (m, 2H), 7.55 (s, 1H), 10.9 (s, 1H); ^13^C NMR (100 MHz, CDCl_3_) δ 51.7, 101.0, 103.3, 115.9 (d, *J* = 22 Hz), 118.9, 130.9 (d, *J* = 8 Hz), 132.2, 133.9 (d, *J* = 3 Hz), 150.6, 162.2 (d, J = 247 Hz), 162.7, 170.4; HRMS (ESI+) m/z calcd for C_14_H_13_FNO_3_ [M + H]^+^ 262.0874, found: 262.0886.

**Methyl 6-amino-4-hydroxy-4'-methoxy-[1,1'-biphenyl]-3-carboxylate** **(26)**

**26** (184 mg, 49%) was prepared according to general procedure 1 and was obtained after purification by flash column chromatography (100% CH_2_Cl_2_). R_f_ 0.25 (100% CH_2_Cl_2_); ^1^H NMR (400 MHz, CDCl_3_) δ 3.84 (s, 3H), 3.86 (s, 3H), 4.21 (br. s, 2H), 6.23 (s, 1H), 6.97 (m,2H), 7.30 (m, 2H), 7.56 (s, 1H), 10.9 (s, 1H); ^13^C NMR (100 MHz, CDCl_3_) δ 51.6, 55.3, 100.7, 103.0, 114.3, 119.6, 130.2, 130.3, 132.1, 150.8, 158.9, 162.5, 170.5; HRMS (ESI+) m/z calcd for C_15_H_16_NO_4_ [M + H]^+^ 274.1074, found: 274.1061.

**General procedure 2: iodination of 24-26**

To a 0°C solution of the appropriate coupled product (1.0 eq.) in MeCN (7.3 mL/mmol) was added *N*-iodosuccinimide (1.0 eq.). After stirring for 2h at 0°C, the mixture was concentrated to dryness. The residue was partitioned between CH_2_Cl_2_ and sat. aq. NaHCO_3_, and the aqueous phase was extracted twice. The combined organic layers were dried (MgSO_4_) and concentrated under reduced pressure. The product was obtained after purification by flash column chromatography.

**Methyl 6-amino-4-hydroxy-5-iodo-[1,1'-biphenyl]-3-carboxylate** **(27)**

**27** (272 mg, 90%) was prepared according to general procedure 2 and was obtained after purification by flash column chromatography (hexane/CH_2_Cl_2_ 2:8). R_f_ 0.8 (100% CH_2_Cl_2_); ^1^H NMR (400 MHz, CDCl_3_) δ 3.91 (s, 3H), 4.90 (br. s, 2H), 7.39 (m, 3H), 7.47 (m, 2H), 7.60 (s, 1H), 12.0 (s, 1H); ^13^C NMR (100 MHz, CDCl_3_) δ 52.1, 73.0, 102.6, 119.3, 127.8, 129.1, 129.3, 131.4, 138.2, 150.6, 161.4, 170.2; HRMS (ESI+) m/z calcd for C_14_H_13_INO_3_ [M + H]^+^ 369.9935, found: 369.9927.

**Methyl 6-amino-4'-fluoro-4-hydroxy-5-iodo-[1,1'-biphenyl]-3-carboxylate** **(28)**

**28** (248 mg, 83%) was prepared according to general procedure 2 and was obtained after purification by flash column chromatography (hexane/CH_2_Cl_2_ 2:8). R_f_ 0.7 (100% CH_2_Cl_2_); ^1^H NMR (400 MHz, CDCl_3_) δ 3.89 (s, 3H), 4.82 (br. s, 2H), 7.14 (m, 2H), 7.34 (m, 2H), 7.54 (s, 1H), 12.0 (s, 1H); ^13^C NMR (100 MHz, CDCl_3_) δ 52.1, 73.1, 102.6, 116.1 (d, *J* = 22 Hz), 118.2, 131.1 (d, *J* = 8 Hz), 131.5, 134.1 (d, *J* = 4 Hz), 150.6, 161.5, 162.4 (d, *J* = 247 Hz), 170.1; HRMS (ESI+) m/z calcd for C_14_H_12_FINO_3_ [M + H]^+^ 387.9840, found: 387.9827.

**Methyl 6-amino-4-hydroxy-5-iodo-4'-methoxy-[1,1'-biphenyl]-3-carboxylate** **(29)**

**29** (131 mg, 60%) was prepared according to general procedure 2 and was obtained after purification by flash column chromatography (hexane/CH_2_Cl_2_ 4:6). R_f_ 0.7 (hexane/CH_2_Cl_2_, 4:6); ^1^H NMR (400 MHz, CDCl_3_) δ 3.85 (s, 3H), 3.88 (s, 3H), 4.86 (br. s, 2H), 6.98 (m, 2H), 7.28 (m, 2H), 7.54 (s, 1H), 11.9 (s, 1H); ^13^C NMR (100 MHz, CDCl_3_) δ 52.1, 55.4, 72.9, 102.4, 105.7, 114.5, 119.0, 130.3, 131.3, 150.9, 159.2, 161.3, 170.2; HRMS (ESI+) m/z calcd for C_15_H_15_INO_4_ [M + H]^+^ 400.0040, found: 400.0067.

**General procedure 3: synthesis of pyrrole derivatives 30-32**

To a solution of the appropriate iodoaniline derivative (1.0 eq.) in AcOH (6.2 mL/mmol) was added 2,5-dimethoxytetrahydrofuran (1.1 eq.). The resulting mixture was stirred at 90°C for 5h, then concentrated under reduced pressure. The residue was partitioned between CH_2_Cl_2_ and sat. aq. NaHCO_3_, and the aqueous phase was extracted twice. The combined organic layers were dried (MgSO_4_) and concentrated under reduced pressure. The product was obtained after purification by flash column chromatography.

**Methyl 4-hydroxy-5-iodo-6-(1H-pyrrol-1-yl)-[1,1'-biphenyl]-3-carboxylate** **(30)**

**30** (262 mg, 96%) was prepared according to general procedure 3 and was obtained after purification by flash column chromatography (hexane/CH_2_Cl_2_ 2:8). R_f_ 0.65 (hexane/CH_2_Cl_2_, 2:8); ^1^H NMR (400 MHz, CDCl_3_) δ 4.02 (s, 3H), 6.17 (m, 2H), 6.53 (m, 2H), 7.01 (m, 2H), 7.23 (m, 3H), 7.97 (s, 1H), 11.85 (s, 1H); ^13^C NMR (100 MHz, CDCl_3_) δ 53.0, 91.0, 109.3, 111.9, 122.0, 127.4, 127.7, 128.1, 131.9, 132.8, 137.5, 147.8, 160.7, 169.6; HRMS (ESI+) m/z calcd for C_18_H_15_INO_3_ [M + H]^+^ 420.0091, found: 420.0101.

**Methyl 4'-fluoro-4-hydroxy-5-iodo-6-(1H-pyrrol-1-yl)-[1,1'-biphenyl]-3-carboxylate** **(31)**

**31** (207 mg, 92%) was prepared according to general procedure 3 and was obtained after purification by flash column chromatography (hexane/CH_2_Cl_2_ 2:8). R_f_ 0.7 (hexane/CH_2_Cl_2_, 2:8); ^1^H NMR (400 MHz, CDCl_3_) δ 4.03 (s, 3H), 6.19 (m, 2H), 6.53 (m, 2H), 6.92 (m, 2H), 6.98 (m, 2H), 7.94 (s, 1H), 11.9 (s, 1H); ^13^C NMR (100 MHz, CDCl_3_) δ 53.1, 91.2, 109.5, 112.0, 115.1 (d, *J* = 22 Hz), 121.8, 129.4 (d, *J* = 8 Hz), 131.7 (2C), 133.4 (d, *J* = 3Hz), 147.8, 160.8, 162.2 (d, *J* = 247 Hz), 169.5; HRMS (ESI+) m/z calcd for C_18_H_14_FINO_3_ [M + H]^+^ 437.9997, found: 437.9993.

**Methyl 4-hydroxy-5-iodo-4'-methoxy-6-(1H-pyrrol-1-yl)-[1,1'-biphenyl]-3-carboxylate** **(32)**

**32** (86 mg, 76%) was prepared according to general procedure 3 and was obtained after purification by flash column chromatography (hexane/CH_2_Cl_2_ 4:6). R_f_ 0.6 (hexane/CH_2_Cl_2_, 2:8); ^1^H NMR (400 MHz, CDCl_3_) δ 3.77 (s, 3H), 4.01 (s, 3H), 6.19 (m, 2H), 6.54 (m, 2H), 6.75 (m, 2H), 6.92 (m, 2H), 7.94 (s, 1H), 11.8 (s, 1H); ^13^C NMR (100 MHz, CDCl_3_) δ 53.0, 55.2, 91.1, 109.3, 112.0, 113.6, 121.9, 128.9, 129.8, 131.8, 132.5, 147.7, 159.0, 160.4, 169.7; HRMS (ESI+) m/z calcd for C_19_H_17_INO_4_ [M + H]^+^ 450.0197, found: 450.0203.

**General procedure 4: synthesis of 33-35**

To a solution of the appropriate ester (1.0 eq.) in THF (8.4 mL/mmol) was added NaOH (1N NaOH, 8.4 mL/mmol). The resulting mixture was stirred at rt until complete consumption of the starting material. The reaction mixture was then diluted with CHCl_3_ and H_2_O. The pH of the aqueous phase was adjusted to ca. 2-3, followed by extraction with CHCl_3_ (4x). The combined organic layers were dried (MgSO_4_) and concentrated under reduced pressure. The product was obtained after purification by flash column chromatography.

**4-hydroxy-5-iodo-6-(1H-pyrrol-1-yl)-[1,1'-biphenyl]-3-carboxylic acid** **(33)**

**33** (97 mg, quant.) was prepared according to general procedure 4 and was obtained after purification by flash column chromatography (gradient 100% CH_2_Cl_2_ to CH_2_Cl_2_/MeOH 8:2). R_f_ 0.5 (CH_2_Cl_2_/MeOH, 8:2); ^1^H NMR (400 MHz, DMSO-*d_6_*) δ 6.00 (m, 2H), 6.56 (m, 2H), 7.00 (m, 2H), 7.15 (m, 3H), 7.78 (s, 1H); ^13^C NMR (100 MHz, DMSO-*d_6_*) δ 92.3, 108.4, 117.6, 122.2, 126.5, 127.6, 127.8, 128.8, 131.5, 138.7, 144.4, 163.5, 170.0; HRMS (ESI+) m/z calcd for C_17_H_13_INO_3_ [M + H]^+^ 405.9935, found: 405.9925.

**4'-fluoro-4-hydroxy-5-iodo-6-(1H-pyrrol-1-yl)-[1,1'-biphenyl]-3-carboxylic acid** **(34)**

**34** (97 mg, quant.) was prepared according to general procedure 4 and was obtained after purification by flash column chromatography (gradient 100% CH_2_Cl_2_ to CH_2_Cl_2_/MeOH 8:2). R_f_ 0.5 (CH_2_Cl_2_/MeOH, 8:2); ^1^H NMR (400 MHz, DMSO-*d_6_*) δ 6.03 (m, 2H), 6.49 (m, 2H), 6.94-7.09 (m, 4H), 7.77 (s, 1H); ^13^C NMR (100 MHz, DMSO-*d_6_*) δ 92.3, 108.7, 114.7 (d, *J* = 21 Hz), 115.2, 122.1, 129.3, 129.7 (d, *J* = 8 Hz), 131.4, 134.5 (d, *J* = 3 Hz), 145.8, 161.2 (d, *J* = 244 Hz), 162.3, 170.5; HRMS (ESI+) m/z calcd for C_17_H_10_FINNa_2_O_3_ [M + 2Na - H]^+^ 467.9479, found: 467.9419.

**4-hydroxy-5-iodo-4'-methoxy-6-(1H-pyrrol-1-yl)-[1,1'-biphenyl]-3-carboxylic acid** **(35)**

**35** (63 mg, quant.) was prepared according to general procedure 4 and was obtained after purification by flash column chromatography (gradient 100% CH_2_Cl_2_ to CH_2_Cl_2_/MeOH 8:2). R_f_ 0.4 (CH_2_Cl_2_/MeOH, 8:2); ^1^H NMR (400 MHz, DMSO-*d_6_*) δ 3.69 (s, 3H), 6.01 (m, 2H), 6.54 (m, 2H), 6.71 (m, 2H), 6.89 (m, 2H), 7.68 (s, 1H); ^13^C NMR (100 MHz, DMSO-*d_6_*) δ 54.9, 92.5, 108.2, 113.2, 118.7, 122.1, 127.5, 128.7, 131.2, 131.3, 143.6, 157.8, 164.2, 169.7; HRMS (ESI+) m/z calcd for C_18_H_13_INNa_2_O_4_ [M + 2Na - H]^+^ 479.9679, found: 479.9642.

**Oxyether linker**

**7-amino-6-((tert-butyldimethylsilyl)oxy)-2,2-dimethyl-4H-benzo[d][1,3]dioxin-4-one (37)**

To an ice cold solution of aminophenol derivative **36** (2.10 g, 10.0 mmol, 1 eq.) in anhydrous CH_2_Cl_2_ (45 mL) and under argon were successively added Tert-butyldimethylsilyl chloride (1.82 g, 12.0 mmol, 1.2 eq.) and imidazole (817 mg, 12.0 mmol, 1.2 eq.). The resulting mixture was stirred at 0°C for 15 minutes, then at rt until complete conversion (ca. 2h).The reaction mixture was then diluted with CH_2_Cl_2_, washed with water (1x) and brine (2x). The organic phase was dried (MgSO_4_) and concentrated under reduced pressure. The product (2.86 g, 88%) was obtained as a white solid after purification by flash column chromatography (hexane/AcOEt 7:3). R_f_ 0.45 (hexane/AcOEt, 7:3); ^1^H NMR (400 MHz, CDCl_3_) δ 0.25 (s, 6H), 1.00 (s, 9H), 1.67 (s, 6H), 4.40 (br. s, 2H), 6.16 (s, 1H), 7.19 (s, 1H); ^13^C NMR (100 MHz, CDCl_3_) δ -4.4, 18.2, 25.7, 25.8, 100.6, 101.8, 105.7, 116.5, 137.8, 146.9, 152.5, 161.4; HRMS (ESI+) m/z calcd for C_16_H_26_NO_4_Si [M + H]^+^ 324.1626, found: 324.1638.

**7-amino-6-((tert-butyldimethylsilyl)oxy)-8-iodo-2,2-dimethyl-4H-benzo[d][1,3]dioxin-4-one (38)**

To an ice cold solution of intermediate **37** (1.80 g, 5.56 mmol, 1 eq.) in MeCN (60 mL) was added *N*-iodosuccinimide (2.50 g, 11.1 mmol, 2.0 eq.) in 3 portions over 5 minutes. The reaction mixture was then stirred at rt overnight. The mixture was then diluted with sat. aq. NaHCO_3_ and aq. Na_2_S_2_O_3_, and extracted twice with CHCl_3_. The combined organic layers were dried (MgSO_4_) and concentrated. The product (2.45 g, 98%) was obtained as a white solid after purification by flash column chromatography (hexane/AcOEt, 8:2). R_f_ 0.45 (hexane/AcOEt, 8:2); ^1^H NMR (400 MHz, CDCl_3_) δ 0.27 (s, 6H), 1.01 (s, 9H), 1.73 (s, 6H), 4.94 (br. s, 2H), 7.20 (s, 1H); ^13^C NMR (100 MHz, CDCl_3_) δ -4.4, 18.2, 25.7 (2C), 70.2, 101.6, 106.4, 115.6, 136.8, 147.7, 151.8, 160.8; HRMS (ESI+) m/z calcd for C_16_H_25_INO_4_Si [M + H]^+^ 450.0593, found: 450.0594.

**6-((tert-butyldimethylsilyl)oxy)-8-iodo-2,2-dimethyl-7-(1H-pyrrol-1-yl)-4H-benzo[d][1,3]dioxin-4-one (39)**

A solution of aniline **38** (1.30 g, 2.89 mmol, 1 eq.) and 2,5-dimethoxytetrahydrofuran (375 µL, 2.89 mmol, 1.0 eq.) in AcOH (14 mL) was stirred at 80°C until the conversion was complete (ca. 7h) then concentrated under reduced pressure. The product (1.40 g, 97%) was obtained as a white solid after purification by flash column chromatography (hexane/AcOEt, 1:1). R_f_ 0.4 (hexane/AcOEt, 9:1); ^1^H NMR (400 MHz, CDCl_3_) δ 0.06 (s, 6H), 0.79 (s, 9H), 1.79 (s, 6H), 6.32 (m, 2H), 6.66 (m, 2H), 7.49 (s, 1H); ^13^C NMR (100 MHz, CDCl_3_) δ -4.9, 17.8, 25.2, 25.9, 90.0, 107.4, 109.3, 113.1, 119.3, 121.5, 143.1, 147.6, 150.4, 159.9; HRMS (ESI+) m/z calcd for C_20_H_27_INO_4_Si [M + H]^+^ 500.0749, found: 500.0750.

**6-hydroxy-8-iodo-2,2-dimethyl-7-(1H-pyrrol-1-yl)-4H-benzo[d][1,3]dioxin-4-one (40)**

To a solution of protected intermediate **39** (1.50 g, 3.00 mmol, 1 eq.) in anhydrous DMF and under argon was added TBAF (1M/THF, 3.3 mL, 1.1 eq.). The reaction mixture was stirred at rt. Following full conversion, the reaction mixture was diluted with AcOEt and washed 3 times with 1:1 H_2_O/brine. The organic layer was then dried (MgSO_4_) and concentrated under reduced pressure. The product (1.10 g, 96%) was obtained as a light yellow solid after purification by flash column chromatography (hexane/AcOEt, 7:3). R_f_ 0.15 (hexane/AcOEt, 8:2); ^1^H NMR (400 MHz, CDCl_3_) δ 1.79 (s, 6H), 5.08 (br. s, 2H), 6.49 (m, 2H), 6.71 (m, 2H), 7.64 (s, 1H); ^13^C NMR (100 MHz, CDCl_3_) δ 25.9, 88.8, 107.5, 111.6, 114.6, 116.2, 121.1, 137.6, 148.1, 149.8, 159.7; HRMS (ESI+) m/z calcd for C_14_H_13_INO_4_ [M + H]^+^ 385.9884, found: 385.9881.

**General procedure 5:** To a solution of protected intermediate **39** (1 eq.) in anhydrous DMF (5mL/mmol) under argon were successively added potassium fluoride (2 eq.) and the appropriate alkyl iodide (1.5 eq.). The resulting mixture was stirred at rt until full conversion of the starting material, as assessed by LCMS and TLC. The mixture was then diluted with water and extracted twice with CH_2_Cl_2_. The combined organic layers were dried (MgSO_4_) and concentrated. The product was obtained by purification by flash column chromatography. KF could be substituted to TBAF if necessary.

**General procedure 6:** To a solution of phenol intermediate **40** (1 eq.), triphenylphosphine (1 eq.) and the appropriate alcohol derivative in anhydrous THF (6.5 mL/mmol) and under argon was added dropwise over 5 minutes a second solution of diethylazodicarboxylate (1.0 eq.) in anhydrous THF (6.5 mL/mmol). The resulting mixture was stirred at rt until the conversion was complete. The reaction mixture was then concentrated to dryness and the product was purified by flash column chromatography.

**6-ethoxy-8-iodo-2,2-dimethyl-7-(1H-pyrrol-1-yl)-4H-benzo[d][1,3]dioxin-4-one (41)**

**41** (299 mg, 90%) was prepared according to general procedure 5 and was obtained as a white solid after purification by flash column chromatography (hexane/AcOEt, 8:2). Iodoethane was used as the electrophile. R_f_ 0.4 (hexane/AcOEt, 8:2); ^1^H NMR (400 MHz, CDCl_3_) δ 1.26 (t, *J* = 7 Hz, 3H), 1.80 (s, 6H), 3.99 (q, *J* = 7 Hz, 2H), 6.35 (m, 2H), 6.69 (m, 2H), 7.54 (s, 1H); ^13^C NMR (100 MHz, CDCl_3_) δ 14.4, 25.8, 65.6, 90.3, 107.4, 109.2, 112.9 (2C), 121.6, 140.9, 150.2, 150.8, 160.1; HRMS (ESI+) m/z calcd for C_16_H_17_INO_4_ [M + H]^+^ 414.0197, found: 414.0196.

**8-iodo-2,2-dimethyl-6-propoxy-7-(1H-pyrrol-1-yl)-4H-benzo[d][1,3]dioxin-4-one (42)**

**42** (324 mg, 95%) was prepared according to general procedure 5 and was obtained as a white solid after purification by flash column chromatography (hexane/AcOEt, 85:15). 1-iodopropane was used as the electrophile. R_f_ 0.5 (hexane/AcOEt, 8:2); ^1^H NMR (400 MHz, CDCl_3_) δ 0.87 (t, *J* = 7 Hz, 3H), 1.65 (m, 2H), 1.80 (s, 6H), 3.88 (t, *J* = 7 Hz, 2H), 6.35 (m, 2H), 6.68 (m, 2H), 7.53 (s, 1H); ^13^C NMR (100 MHz, CDCl_3_) δ 10.2, 22.2, 25.8, 71.3, 90.2, 107.4, 109.1, 112.6, 112.9, 121.6, 140.8, 150.1, 151.1, 160.1;

**6-(allyloxy)-8-iodo-2,2-dimethyl-7-(1H-pyrrol-1-yl)-4H-benzo[d][1,3]dioxin-4-one (43)**

**43** (177 mg, 80%) was prepared according to general procedure 5 and was obtained as a white solid after purification by flash column chromatography (hexane/AcOEt, 8:2). R_f_ 0.35 (hexane/AcOEt, 8:2); ^1^H NMR (400 MHz, CDCl_3_) δ 1.80 (s, 6H), 4.48 (m, 1H), 5.16-5.26 (m, 1H), 5.78-5.91 (m, 1H), 6.37 (m, 2H), 6.70 (m, 2H), 7.55 (s, 1H); ^13^C NMR (100 MHz, CDCl_3_) δ 25.8, 70.0, 90.4, 107.5, 109.3, 112.9, 113.3, 117.8, 121.6, 131.7, 141.0, 150.4, 160.0; HRMS (ESI+) m/z calcd for C_17_H_17_INO_4_ [M + H]^+^ 426.0197, found: 426.0193.

**6-butoxy-8-iodo-2,2-dimethyl-7-(1H-pyrrol-1-yl)-4H-benzo[d][1,3]dioxin-4-one (44)**

**44** (148 mg, 65%) was prepared according to general procedure 6 and was obtained as a white solid after purification by flash column chromatography (hexane/AcOEt, 85:15). R_f_ 0.5 (hexane/AcOEt, 8:2); ^1^H NMR (400 MHz, CDCl_3_) δ 0.88 (t, *J* = 7.4 Hz, 3H), 1.26-1.37 (m, 2H), 1.58-1.68 (m, 2H), 1.82 (s, 6H), 3.93 (t, *J* = 6.5 Hz, 2H), 6.36 (m, 2H), 6.69 (m, 2H), 7.55 (s, 1H); ^13^C NMR (100 MHz, CDCl_3_) δ 13.6, 18.9, 25.8, 30.8, 69.6, 90.2, 107.5, 109.2, 112.6, 112.9, 121.6, 140.9, 150.1, 151.1, 160.2; HRMS (ESI+) m/z calcd for C_18_H_21_INO_4_ [M + H]^+^ 442.0510, found: 442.0510.

**8-iodo-2,2-dimethyl-7-(1H-pyrrol-1-yl)-6-(3,3,3-trifluoropropoxy)-4H-benzo[d][1,3]dioxin-4-one (45)**

**45** (115 mg, 46%) was prepared according to general procedure 6 and was obtained as a white solid after purification by flash column chromatography (hexane/AcOEt, 8:2). R_f_ 0.5 (CH_2_Cl_2_, 3:7); ^1^H NMR (400 MHz, CDCl_3_) δ 1.81 (s, 6H), 2.45 (m, 2H), 4.08 (t, *J* = 6.4 Hz, 2H), 6.35 (m, 2H), 6.67 (m, 2H), 7.56 (s, 1H); ^13^C NMR (100 MHz, CDCl_3_) δ 25.8, 33.8 (q, *J* = 29 Hz), 63.0 (q, *J* = 3 Hz), 90.7, 107.7, 109.5, 112.9, 113.2, 121.6, 125.5 (q, *J* = 277 Hz), 141.2, 150.0, 151.1, 159.9; HRMS (ESI+) m/z calcd for C_17_H_16_F_3_INO_4_ [M + H]^+^ 482.0071, found: 482.0074.

**6-(2-hydroxyethoxy)-8-iodo-2,2-dimethyl-7-(1H-pyrrol-1-yl)-4H-benzo[d][1,3]dioxin-4-one (46)**

**46** (256 mg, 74%) was prepared according to general procedure 5 and was obtained as a white solid after purification by flash column chromatography (hexane/AcOEt, 6:4). 2-iodoethan-1-ol was used as the electrophile. R_f_ 0.3 (hexane/AcOEt, 1:1); ^1^H NMR (400 MHz, CDCl_3_) δ 1.74 (br. s, 1H), 1.81 (s, 6H), 3.72 (br. m, 2H), 4.00 (t, *J* = 4 Hz, 2H), 6.38 (m, 2H), 6.71 (m, 2H), 7.58 (s, 1H); ^13^C NMR (100 MHz, CDCl_3_) δ 25.8, 60.9, 72.0, 90.1, 107.6, 109.7, 113.2, 114.1, 121.7, 141.4, 150.7, 150.9, 159.9; HRMS (ESI+) m/z calcd for C_16_H_17_INO_5_ [M + H]^+^ 430.0146, found: 430.0139.

**8-iodo-2,2-dimethyl-6-phenethoxy-7-(1H-pyrrol-1-yl)-4H-benzo[d][1,3]dioxin-4-one (47)**

**47** (380 mg, 85%) was prepared according to general procedure 6 and was obtained as a white solid after purification by flash column chromatography (hexane/CH_2_Cl_2_, 3:7). R_f_ 0.35 (hexane/CH_2_Cl_2_, 3:7); ^1^H NMR (400 MHz, CDCl_3_) δ 1.73 (s, 6H), 2.86 (t, *J* = 6.5 Hz, 2H), 4.04 (t, *J* = 6.5 Hz, 2H), 6.33 (m, 2H), 6.57 (m, 2H), 6.98 (m, 2H), 7.10-7.22 (m, 3H), 7.44 (s, 1H); ^13^C NMR (100 MHz, CDCl_3_) δ 25.8, 35.4, 70.4, 90.4, 107.5, 109.2, 112.2, 112.9, 121.6, 126.4, 128.3, 129.2, 137.7, 140.7, 150.2, 150.8, 160.1; HRMS (ESI+) m/z calcd for C_22_H_21_INO_4_ [M + H]^+^ 490.0510, found: 490.0492.

**8-iodo-6-isobutoxy-2,2-dimethyl-7-(1H-pyrrol-1-yl)-4H-benzo[d][1,3]dioxin-4-one (48)**

**48** (166 mg, 47%) was prepared according to general procedure 6 and was obtained as a white solid after purification by flash column chromatography (hexane/AcOEt, 8:2). 1-iodo-2-methylpropane was used as the electrophile. R_f_ 0.6 (hexane/AcOEt, 8:2); ^1^H NMR (400 MHz, CDCl_3_) δ 0.85 (d, *J* = 7 Hz, 6H), 1.80 (s, 6H), 1.92 (m, 1H), 3.67 (d, *J* = 7Hz, 2H); 6.34 (m, 2H), 6.67 (m, 2H), 7.51 (s, 1H); ^13^C NMR (100 MHz, CDCl_3_) δ 18.8, 25.8, 28.0, 76.0, 90.2, 107.4, 109.1, 112.3, 112.9, 121.6, 140.8, 150.0, 151.2, 160.2; HRMS (ESI+) m/z calcd for C_18_H_21_INO_4_ [M + H]^+^ 442.0510, found: 442.0499.

**Deprotection of intermediates 41-48, General procedure 7:** To a solution of the appropriate protected intermediate in THF (15 mL/mmol) was added 2M KOH (15 mL/mmol) at rt. The resulting solution was stirred at rt until the conversion was complete, as assessed by TLC and/or LCMS. Work up and purification 1: The solution was diluted with water and acidified until pH < 4 with concentrated HCl, and extracted 3 times with 4:1 CHCl_3_/iPrOH. The combined organic layers were dried (MgSO_4_) and concentrated, and the product was purified by flash column chromatography. Work up and purification 2: After completion of the reaction, the reaction was neutralised and THF was removed under reduced pressure. The resulting solution was diluted with MeCN and the product was purified on reverse phase HPLC.

**5-ethoxy-2-hydroxy-3-iodo-4-(1H-pyrrol-1-yl)benzoic acid (49)**

**49** (85 mg, 94%) was prepared according to general procedure 7 and was obtained as a white solid after purification by flash column chromatography (CH_2_Cl_2_/MeOH, 75:25). R_f_ 0.3 (CH_2_Cl_2_/MeOH, 8:2); ^1^H NMR (400 MHz, DMSO-*d_6_*) δ 1.06 (t, *J* = 7.0 Hz, 3H), 3.78 (q, *J* = 7.0 Hz, 2H), 6.14 (m, 2H), 6.64 (m, 2H), 7.48 (s, 1H); ^13^C NMR (100 MHz, DMSO-*d_6_*) δ 14.7, 65.7, 90.8, 108.0, 116.4, 118.8, 121.9, 136.8, 145.4, 158.0, 169.9; HRMS (ESI+) m/z calcd for C_13_H_13_INO_4_ [M + H]^+^ 373.9884, found: 373.9862.

**2-hydroxy-3-iodo-5-propoxy-4-(1H-pyrrol-1-yl)benzoic acid (50)**

**50** (86 mg, 95%) was prepared according to general procedure 7 and was obtained as a white solid after purification by flash column chromatography (CH_2_Cl_2_/MeOH, 8:2). R_f_ 0.45 (CH_2_Cl_2_/MeOH, 8:2); ^1^H NMR (400 MHz, DMSO-*d_6_*) δ 0.76 (t, J = 7.3 Hz, 3H), 1.48 (m, 2H), 3.74 (t, *J* = 6.3 Hz, 2H), 6.17 (m, 2H), 6.67 (m, 2H), 7.48 (s, 1H); ^13^C NMR (100 MHz, DMSO-*d_6_*) δ 10.2, 22.0, 71.2, 91.3, 108.2, 115.3, 115.8, 121.8, 138.1, 146.5, 156.8, 170.3; HRMS (ESI+) m/z calcd for C_14_H_13_INNa_2_O_4_ [M + 2Na - H]^+^ 431.9679, found: 431.9666.

**5-(allyloxy)-2-hydroxy-3-iodo-4-(1H-pyrrol-1-yl)benzoic acid (51)**

**51** (66 mg, 91%) was prepared according to general procedure 7 and was obtained as a white solid after purification by flash column chromatography (CH_2_Cl_2_/MeOH, 8:2). R_f_ 0.4 (CH_2_Cl_2_/MeOH, 8:2); ^1^H NMR (400 MHz, DMSO-*d_6_*) δ 4.31 (m, 2H), 5.04-5.17 (m, 2H), 5.73-5.88 (m, 1H), 6.16 (m, 2H), 6.66 (m, 2H), 7.49 (s, 1H); ^13^C NMR (100 MHz, DMSO-*d_6_*) δ 69.9, 90.9, 108.1, 116.0, 116.5, 118.6, 121.9, 133.6, 136.6, 145.2, 157.9, 169.8; HRMS (ESI+) m/z calcd for C_14_H_11_INNa_2_O_4_ [M + 2Na - H]^+^ 429.9523, found: 429.9523.

**5-butoxy-2-hydroxy-3-iodo-4-(1H-pyrrol-1-yl)benzoic acid (52)**

**52** (62 mg, 85%) was prepared according to general procedure 7 and was obtained as a white solid after purification by flash column chromatography (CH_2_Cl_2_/MeOH, 8:2). R_f_ 0.4 (CH_2_Cl_2_/MeOH, 8:2); ^1^H NMR (400 MHz, DMSO-*d_6_*) δ 0.79 (t, *J* = 7.6 Hz, 3H), 1.20 (m, 2H), 1.43 (m, 2H), 3.73 (t, *J* = 6.2 Hz, 2H), 6.15 (m, 2H), 6.63 (m, 2H), 7.48 (s, 1H); ^13^C NMR (100 MHz, DMSO-*d_6_*) δ 13.6, 18.4, 30.8, 69.5, 90.9, 108.0, 115.9, 118.2, 121.9, 136.9, 145.9, 157.6, 169.9; HRMS (ESI+) m/z calcd for C_15_H_15_INNa_2_O_4_ [M + 2Na - H]^+^ 445.9836, found: 445.9823.

**2-hydroxy-3-iodo-4-(1H-pyrrol-1-yl)-5-(3,3,3-trifluoropropoxy)benzoic acid (53)**

**53** (49 mg, 71%) was prepared according to general procedure 7 and was obtained as a white solid after purification by flash column chromatography (CH_2_Cl_2_/MeOH, 85:15). R_f_ 0.3 (CH_2_Cl_2_/MeOH, 8:2); ^1^H NMR (400 MHz, DMSO-*d_6_*) δ 2.45 (m, 2H), 3.89 (t, *J* = 6.3 Hz, 2H), 6.16 (m, 2H), 6.66 (m, 2H), 7.51 (s, 1H); ^13^C NMR (100 MHz, DMSO-*d_6_*) δ 33.0 (q, *J* = 28 Hz), 63.7 (q, *J* = 3 Hz), 91.2, 108.2, 117.0, 118.5, 121.9, 126.3 (q, *J* = 277 Hz), 137.2, 144.8, 158.9, 169.6; HRMS (ESI+) m/z calcd for C_14_H_12_F_3_INO_4_ [M + H]^+^ 441.9758, found: 441.9740.

**2-hydroxy-5-(2-hydroxyethoxy)-3-iodo-4-(1H-pyrrol-1-yl)benzoic acid (54)**

**54** (71 mg, 78%) was prepared according to general procedure 7 and was obtained as a white solid after purification by flash column chromatography (CH_2_Cl_2_/MeOH, 75:25). R_f_ 0.25 (CH_2_Cl_2_/MeOH, 8:2); ^1^H NMR (400 MHz, DMSO-*d_6_*) δ 3.46 (t, J = 5.3 Hz, 2H), 3.76 (t, J = 5.3 Hz, 2H), 6.16 (m, 2H), 6.69 (m, 2H), 7.54 (s, 1H); ^13^C NMR (100 MHz, DMSO-*d_6_*) δ 59.5, 72.3, 90.9, 108.2, 116.8, 117.1, 121.9, 137.8, 146.1, 157.6, 170.1; HRMS (ESI+) m/z calcd for C_13_H_11_INNa_2_O_5_ [M + 2Na - H]^+^ 433.9472, found: 433.9463.

**2-hydroxy-3-iodo-5-phenethoxy-4-(1H-pyrrol-1-yl)benzoic acid (55)**

**55** (84 mg, 91%) was prepared according to general procedure 7 and was obtained as a white solid after purification by flash column chromatography (CH_2_Cl_2_/MeOH, 8:2). R_f_ 0.5 (CH_2_Cl_2_/MeOH, 8:2); ^1^H NMR (400 MHz, DMSO-*d_6_*) δ 2.77 (t, *J* = 6.5 Hz, 2H), 3.95 (t, *J* = 6.5 Hz, 2H), 6.20 (m, 2H), 6.63 (m, 2H), 7.07 (m, 2H), 7.13-7.25 (m, 3H), 7.47 (s, 1H); ^13^C NMR (100 MHz, DMSO-*d_6_*) δ 35.0, 70.3, 91.3, 108.2, 115.2, 116.8, 121.9, 126.0, 128.1, 129.0, 137.4, 138.2, 145.9, 157.3, 170.0; HRMS (ESI+) m/z calcd for C_19_H_15_INNa_2_O_4_ [M + 2Na - H]^+^ 493.9836, found: 493.9827.

**2-hydroxy-3-iodo-5-isobutoxy-4-(1H-pyrrol-1-yl)benzoic acid (56)**

**56** (89 mg, 98%) was prepared according to general procedure 7 and was obtained as a white solid after purification by flash column chromatography (CH_2_Cl_2_/MeOH, 8:2). R_f_ 0.45 (CH_2_Cl_2_/MeOH, 8:2); ^1^H NMR (400 MHz, DMSO-*d_6_*) δ 0.76 (d, *J* = 6.7 Hz, 6H), 1.75 (m, 1H), 3.56 (d, *J* = 6.2 Hz, 2H), 6.17 (m, 2H), 6.66 (d, 2H), 7.47 (s, 1H); ^13^C NMR (100 MHz, DMSO-*d_6_*) δ 18.7, 27.7, 75.7, 91.2, 108.2, 114.8, 115.8, 121.8, 137.9, 146.7, 156.6, 170.4; HRMS (ESI+) m/z calcd for C_15_H_15_INNa_2_O_4_ [M + 2Na - H]^+^ 445.9836, found: 445.9825.

**Thioether-linker**

**methyl 4-amino-2-methoxy-5-(propylthio)benzoate (58)**

To a solution of methyl 4-amino-5-mercapto-2-methoxybenzoate **57** (1.0 g, 4.69 mmol, 1 eq.) at 0°C in anhydrous DMF (15 mL) and under argon were successively added iodopropane (458 μL, 4.69 mmol, 1 eq.) and caesium carbonate (1.53 g, 4.69 mmol, 1 eq.). The resulting mixture was stirred overnight until the temperature reaches ca. 10°C. The mixture was partitioned between CH_2_Cl_2_ and brine and the aqueous phase was extracted twice. The combined organic layers were dried (MgSO_4_) and concentrated *in vacuo*. The product (494 mg, 41%) was obtained after purification by flash column chromatography (hexane/AcOEt 6:4). R_f_ 0.4 (hexane/AcOEt, 1:1); ^1^H NMR (400 MHz, CDCl_3_) δ 0.97 (t, *J* = 7.3 Hz, 3H), 1.55 (m, 2H), 2.61 (t, *J* = 7.3 Hz, 2H), 3.82 (s, 3H), 3.84 (s, 3H), 4.84 (br. s, 2H), 6.24 (s, 1H), 7.99 (s, 1H); ^13^C NMR (100 MHz, CDCl_3_) δ 13.3, 22.9, 37.7, 51.5, 55.9, 97.0, 108.6, 109.4, 141.6, 153.6, 161.9, 165.6; HRMS (ESI+) m/z calcd for C_12_H_18_NO_3_S [M + H]^+^ 256.1002, found: 256.1025.

**4-amino-2-hydroxy-5-(propylthio)benzoic acid (59)**

To a solution of **58** (440 mg, 1.72 mmol, 1 eq.) in anhydrous CH_2_Cl_2_ (10 mL) at 0°C and under argon was added BBr_3_ (1M/CH_2_Cl_2_, 1.9 mL, 1.9 mmol, 1.1 eq.) dropwise. The resulting mixture was stirred at 0°C for 2h. The mixture was partitioned between sat. aq. NaHCO_3_ and CHCl_3_, and the aqueous phase was extracted twice. The combined organic layers were dried (MgSO_4_) and concentrated *in vacuo*. The product (300 mg, 77%) was obtained after purification by flash column chromatography (CH_2_Cl_2_/MeOH 8:2). R_f_ 0.6 (CH_2_Cl_2_/MeOH 8:2); ^1^H NMR (400 MHz, DMSO-*d_6_*) δ 0.91 (t, *J* = 7.2 Hz, 3H), 1.44 (m, 2H), 2.56 (t, *J* = 7.2 Hz, 2H), 6.15 (s, 1H), 6.17 (br. s, 2H), 7.68 (s, 1H); ^13^C NMR (100 MHz, DMSO-*d_6_*) δ 13.0, 22.1, 36.2, 98.9, 102.2, 106.8, 139.0, 155.9, 163.3, 171.6; HRMS (ESI+) m/z calcd for C_10_H_14_NO_3_S [M + H]^+^ 228.0689, found: 228.0667.

**2-hydroxy-3-iodo-5-(propylthio)-4-(1H-pyrrol-1-yl)benzoic acid (60)**

To a solution of **59** (200 mg, 0.880 mmol, 1 eq.) in MeCN (10 mL) at 0°C was added *N*-iodosuccinimide (198 mg, 0.880 mmol, 1 eq.). The resulting mixture was stirred at 0°C for 1h and concentrated to dryness. The residue was dissolved in AcOH (6 mL) and dimethoxytetrahydrofuran (148 μL, 1.14 mmol, 1.3 eq.) was added. The resulting mixture was stirred at 80°C for 5h and concentrated *in vacuo*. The product (107 mg, 30% over 2 steps) was purified by reverse phase HPLC. ^1^H NMR (400 MHz, DMSO-*d_6_*) δ 0.87 (t, *J* = 7.5 Hz, 3H), 1.45 (m, 2H), 2.65 (t, *J* = 7.5 Hz, 2H), 6.23 (m, 2H), 6.68 (m, 2H), 7.82 (s, 1H); ^13^C NMR (100 MHz, DMSO-*d_6_*) δ 13.1, 21.5, 34.7, 92.8, 109.0, 113.5, 121.1, 126.1, 130.5, 148.3, 159.7, 170.7; HRMS (ESI+) m/z calcd for C_14_H_13_INNa_2_O_3_S [M + 2Na - H]^+^ 447.9451, found: 447.9439.

**Aminobenzothiazoles**

**Methyl 2-amino-5-methoxybenzo[d]thiazole-4-carboxylate (62)**

A solution of methyl 2-amino-6-methoxybenzoate **61** (4.30 g, 23.7 mmol, 1.0 eq.) and potassium thiocyanate (6.92 g, 71.2 mmol, 3.0 eq.) in AcOH (85 mL) was cooled to ca. 10°C using a water bath. A second solution of bromine (1.34 mL, 26.1 mmol, 1.1 eq.) in AcOH (85 mL) was added dropwise over 1 hour. The resulting mixture was stirred at rt and the progress of the reaction was followed by LCMS. After stirring for 48h, the reaction mixture was concentrated to dryness. The residue was partitioned between sat. aq. NaHCO_3_ and AcOEt, and the aqueous phase was extracted 3 times. The combined organic layers were dried (MgSO_4_) and concentrated to dryness. The residue was suspended in CH_2_Cl_2_. The product (2.43 g, 43%) was filtered off, washed with CH_2_Cl_2_, and used without further purification. R_f_ 0.5 (CH_2_Cl_2_/MeOH 9:1); ^1^H NMR (400 MHz, DMSO-*d_6_*) δ 3.77 (s, 3H), 3.78 (s, 3H), 6.78 (d, J = 8.8 Hz, 1H), 7.63 (d, J = 8.8 Hz, 1H), 7.74 (br.s, 2H); ^13^C NMR (100 MHz, DMSO-*d_6_*) δ 51.9, 56.1, 104.9, 112.9, 122.1, 123.1, 150.7, 154.5, 166.8, 168.7; HRMS (ESI+) m/z calcd for C_10_H_11_N_2_O_3_S [M + H]^+^ 239.0485, found: 239.0508.

**Methyl 2-amino-5-methoxy-6-nitrobenzo[d]thiazole-4-carboxylate (63)**

**62** (2.85 g, 12.0 mmol, 1.0 eq.) was added in several portions to fuming nitric acid (20 mL) at 0°C. After stirring for 1.5 h at 0°C, the solution was poured to a separating funnel containing ice cold AcOEt and water. Concentrated NaOH (4N) was added carefully (exothermic) until the pH of the aqueous phase reached ca. 10. The aqueous phase was then extracted 3 times with AcOEt. The combined organic layers were dried (MgSO_4_) and concentrated to dryness, affording the product (3.10 g, 91%) as an orange solid, which was used for the next step without further purification. R_f_ 0.5 (CH_2_Cl_2_/MeOH 9:1); ^1^H NMR (400 MHz, DMSO-*d_6_*) δ 3.83 (s, 3H), 3.87 (s, 3H), 8.50 (br. s, 2H), 8.57 (s, 1H); ^13^C NMR (100 MHz, DMSO-*d_6_*) δ 52.6, 63.5, 118.2, 120.1, 127.0, 135.4, 149.9, 155.2, 165.3, 173.0; HRMS (ESI+) m/z calcd for C_10_H_10_N_3_O_5_S [M + H]^+^ 284.0336, found: 284.0346.

**Methyl 2,6-diamino-5-methoxybenzo[d]thiazole-4-carboxylate (64)**

A suspension of nitro derivative **63** (32.0 g, 113 mmol, 1.0 eq.) in a 1:1 mixture of CH_2_Cl_2_ and AcOEt (1500 mL) was cooled to 0°C. SnCl_2_.2H_2_O (127 g, 565 mmol, 5 eq.) was added in several portions over 20 minutes. The resulting mixture was stirred at rt for 24h, then concentrated *in vacuo*. The residue was partitioned between sat. aq. NaHCO_3_ and AcOEt, and the aqueous phase was extracted 4 times. The combined organic layers were dried (MgSO_4_) and concentrated to dryness. The product (16.7 g, 58%) was obtained as a light yellow solid after purification by flash column chromatography (CH_2_Cl_2_/MeOH 95:5). R_f_ 0.2 (CH_2_Cl_2_/MeOH 95:5); ^1^H NMR (400 MHz, DMSO-*d_6_*) δ 3.66 (s, 3H), 3.80 (s, 3H), 4.79 (br. s, 2H), 7.02 (s, 1H), 7.26 (br. s, 2H); ^13^C NMR (100 MHz, DMSO-*d_6_*) δ 52.0, 60.3, 107.5, 118.1, 127.1, 136.1, 140.7, 141.9, 164.5, 167.0; HRMS (ESI+) m/z calcd for C_10_H_12_N_3_O_3_S [M + H]^+^ 254.0594, found: 254.0594.

**Methyl 2,6-diamino-5-methoxy-7-nitrobenzo[d]thiazole-4-carboxylate (65)**

To an ice cold solution of **64** (4.0 g, 15.8 mmol, 1.0 eq.) in TFA (70 mL) was added KNO_3_ (1.76 g, 17.4 mmol, 1.1 eq.) in several portions. After stirring for 5 hours at rt, the solution was poured to a separating funnel containing ice cold AcOEt and water. Concentrated NaOH (4N) was added carefully (exothermic) until the pH of the aqueous phase reached ca. 10. The aqueous phase was then extracted 3 times with AcOEt. The combined organic layers were dried (MgSO_4_) and concentrated to dryness. The product (3.77 g, 80%) was obtained as a dark red solid after purification by flash column chromatography (CH_2_Cl_2_/MeOH 94:6); R_f_ 0.6 (CH_2_Cl_2_/MeOH 92:8); ^1^H NMR (400 MHz, DMSO-*d_6_*) δ 3.76 (s, 3H), 3.89 (s, 3H), 7.45 (br. S, 2H), 7.49 (br. s, 2H); ^13^C NMR (100 MHz, DMSO-*d_6_*) δ 52.6, 61.6, 121.4, 124.9, 125.4, 137.5, 137.9, 143.1, 165.3, 167.3; HRMS (ESI+) m/z calcd for C_10_H_11_N_4_O_5_S [M + H]^+^ 299.0445, found: 299.0453.

**Methyl 2-amino-6-iodo-5-methoxy-7-nitrobenzo[d]thiazole-4-carboxylate (66)**

A suspension of **65** (3.77 g, 12.6 mmol, 1.0 eq.) in 5% HCl (130 mL) was cooled at 0°C. NaNO_2_ (959 mg, 13.9 mmol, 1.1 eq.) in water (35 mL) was added dropwise. After stirring for 1h at 0°C, a solution of KI (6.29 g, 37.9 mmol, 3.0 eq.) in water (50 mL) was added dropwise. The resulting mixture was stirred at 0°C for 2 hours, then at rt overnight. The reaction mixture was partition between sat. aq. NaHCO_3_ and AcOEt, and the aqueous phase was extracted 3 times. The combined organic layers were dried (MgSO_4_) and concentrated *in vacuo*. The product (4.44 g, 80%) was obtained as an orange solid after purification by flash column chromatography (CH_2_Cl_2_/MeOH 95:5); R_f_ 0.35 (CH_2_Cl_2_/MeOH 95:5); ^1^H NMR (400 MHz, DMSO-*d_6_*) δ 3.81 (s, 3H), 3.89 (s, 3H), 8.34 (br. s, 2H); ^13^C NMR (100 MHz, DMSO-*d_6_*) δ 52.8, 62.6, 78.3, 120.8, 125.2, 145.1, 152.0, 155.7, 165.0, 170.1; HRMS (ESI+) m/z calcd for C_10_H_9_IN_3_O_5_S [M + H]^+^ 409.9302, found: 409.9298.

**Methyl 2,7-diamino-6-iodo-5-methoxybenzo[d]thiazole-4-carboxylate (67)**

To a solution of **66** (4.32 g, 10.6 mmol, 1.0 eq.) in AcOH (80 mL) was added iron (4.12 g, 73.9 mmol, 7.0 eq.) at rt. The resulting mixture was stirred at 50°C for 5 hours, then concentrated to dryness. The residue was partitioned between sat. aq. NaHCO_3_ and AcOEt, and the aqueous phase was extracted 3 times. The combined organic layers were dried (MgSO_4_) and concentrated to dryness. The product (3.70 g, 92%) was used for the next step without further purification. R_f_ 0.25 (CH_2_Cl_2_/MeOH 94:6); ^1^H NMR (400 MHz, DMSO-*d_6_*) δ 3.70 (s, 3H), 3.76 (s, 3H), 5.63 (br. s, 2H), 7.70 (br. s, 2H); ^13^C NMR (100 MHz, DMSO-*d_6_*) δ 51.9, 61.7, 71.1, 107.7, 109.9, 143.0, 151.2, 155.6, 166.4, 167.9; HRMS (ESI+) m/z calcd for C_10_H_11_IN_3_O_3_S [M + H]^+^ 379.9560, found: 379.9566.

**Methyl 2-amino-6-iodo-5-methoxy-7-(1H-pyrrol-1-yl)benzo[d]thiazole-4-carboxylate (68)**

A solution of **67** (3.70 g, 9.76 mmol, 1.0 eq.) and 2,5-dimethoxytetrahydrofuran (1.26 mL, 9.76 mmol, 1 eq.) in AcOH (120 mL) was stirred at 70°C for 5 hours and concentrated to dryness. The residue was partitioned between sat. aq. NaHCO_3_ and AcOEt, and the aqueous phase was extracted 3 times. The combined organic layers were dried (MgSO_4_) and concentrated to dryness. The product (777 mg, 19%) was obtained as an off-white solid after purification by flash column chromatography (CH_2_Cl_2_/AcOEt 7:3). R_f_ 0.2 (CH_2_Cl_2_/AcOEt 7:3); ^1^H NMR (400 MHz, DMSO-*d_6_*) δ 3.80 (s, 3H), 3.87 (s, 3H), 6.28 (m, 2H), 6.91 (m, 2H), 8.03 (br. s, 2H); ^13^C NMR (100 MHz, DMSO-*d_6_*) δ 52.5, 62.2, 84.9, 109.5, 117.3, 121.1, 127.3, 137.4, 151.0, 155.1, 165.7, 168.7; HRMS (ESI+) m/z calcd for C_14_H_13_IN_3_O_3_S [M + H]^+^ 429.9717, found: 429.9724.

**Methyl 2-(diethylamino)-6-iodo-5-methoxy-7-(1H-pyrrol-1-yl)benzo[d]thiazole-4-carboxylate (69)**

A solution of **68** (900 mg, 3.87 mmol, 1.0 eq.), ethyl iodide (409 μL, 5.11 mmol, 2.2 eq.) and K_2_CO_3_ (802 mg, 5.80 mmol, 2.5 eq.) in anhydrous DMF was heated at 60°C for 3 hours, then partitioned between sat. aq. NaHCO_3_ and CHCl_3_. The aqueous phase was extracted 3 times with CHCl_3_. The combined organic layers were dried (MgSO_4_) and concentrated to dryness. The product (324 mg, 48%) was obtained as a white solid after purification by flash column chromatography (hexane/CH_2_Cl_2_ 1:9). R_f_ 0.5 (CH_2_Cl_2_); ^1^H NMR (400 MHz, DMSO-*d_6_*) δ 1.16 (t, *J* = 7.2 Hz, 6H), 3.48 (q, *J* = 7.2 Hz, 2H), 3.81 (s, 3H), 3.90 (s, 3H), 6.29 (m, 2H), 6.92 (m, 2H); ^13^C NMR (100 MHz, DMSO-*d_6_*) δ 12.4, 45.7, 52.4, 62.3, 84.7, 109.6, 117.4, 121.1, 126.7, 137.6, 151.2, 155.4, 165.6, 167.8; HRMS (ESI+) m/z calcd for C_18_H_21_IN_3_O_3_S [M + H]^+^ 486.0343, found: 486.0381.

**2-(diethylamino)-5-hydroxy-6-iodo-7-(1H-pyrrol-1-yl)benzo[d]thiazole-4-carboxylic acid (70)**

To an ice cold solution of **69** (100 mg, 0.206 mmol, 1.0 eq.) in anhydrous CH_2_Cl_2_ (3 mL) was added BBr_3_ (1M in CH_2_Cl_2_, 515 μL, 0.515 mmol, 2.5 eq.) dropwise. The resulting mixture was stirred overnight and the temperature gradually increased to ca. 10°C. Water was added dropwise and stirring was continued for 5 minutes. The reaction was partitioned between water and 4:1 CHCl_3_/i-PrOH, and the aqueous phase was extracted 3 times. The combined organic layers were dried (MgSO_4_) and concentrated to dryness. The product (74 mg, 79%) was obtained a light yellow solid after purification by flash column chromatography (CH_2_Cl_2_/MeOH 98:2). R_f_ 0.5 (CH_2_Cl_2_/MeOH 98:2); ^1^H NMR (400 MHz, DMSO-*d_6_*) δ 1.22 (t, *J* = 7.0 Hz, 6H), 3.57 (br. q, 4H), 6.33 (m, 2H), 6.98 (m, 2H), 12.6 (br. s, 1H), 14.5 (br. s, 1H); ^13^C NMR (100 MHz, DMSO-*d_6_*) δ 12.0, 46.3, 78.8, 100.0, 110.1, 118.5, 121.1, 141.0, 151.7, 160.8, 169.2, 170.6; HRMS (ESI+) m/z calcd for C_16_H_17_IN_3_O_3_S [M + H]^+^ 458.0030, found: 458.0017.

**Methyl 2-(diethylamino)-5-hydroxy-6-iodo-7-(1H-pyrrol-1-yl)benzo[d]thiazole-4-carboxylate (71)**

To a solution of **70** (90 mg, 0.197 mmol, 1.0 eq.) in MeOH (2 mL) were added DCC (49 mg, 0.236 mmol, 1.2 eq.) and DMAP (2.5 mg, 0.0197 mmol, 0.1 eq.). The resulting mixture was stirred at rt for 48 hours and concentrated to dryness. The product (95 mg, 92%) was obtained as a white solid after purification by flash column chromatography (hexane/CH_2_Cl_2_ 3:7). R_f_ 0.6 (CH_2_Cl_2_); ^1^H NMR (400 MHz, CDCl_3_) δ 1.27 (t, *J* = 7.2 Hz, 6H), 3.56 (br. q, 4H), 4.06 (s, 3H), 6.40 (m, 2H), 6.85 (m, 2H), 12.7 (br. s, 1H); ^13^C NMR (100 MHz, CDCl_3_) δ 12.6, 45.7, 52.8, 77.2, 102.7, 109.8, 121.0, 123.3, 141.0, 154.0, 161.4, 169.6, 171.3; HRMS (ESI+) m/z calcd for C_17_H_19_IN_3_O_3_S [M + H]^+^ 472.0186, found: 472.0228.

**2-(diethylamino)-N-ethyl-5-hydroxy-6-iodo-7-(1H-pyrrol-1-yl)benzo[d]thiazole-4-carboxamide (72)**

To a suspension of **71** (50 mg, 0.106 mmol, 1.0 eq.) in MeOH was added ethylamine (70% aq., 0.5 mL). The resulting mixture was stirred at rt for 1.5 hour and concentrated *in vacuo*. The product (49 mg, 95%) was obtained as a white solid after purification by flash column chromatography (hexane/CH_2_Cl_2_ 1:9). R_f_ 0.45 (hexane/CH_2_Cl_2_ 1:9); ^1^H NMR (400 MHz, CDCl_3_) δ 1.26-1.36 (m, 9H), 3.46-3.60 (m, 6H), 6.40 (m, 2H), 6.83 (m, 2H), 10.4 (br. s, 1H), 15.2 (br. s, 1H); ^13^C NMR (100 MHz, CDCl_3_) δ 12.6, 14.4, 34.3, 46.6, 79.9, 103.8, 110.0, 120.3, 121.2, 139.6, 151.7, 162.3, 169.2, 169.3; HRMS (ESI+) m/z calcd for C_18_H_22_IN_4_O_2_S [M + H]^+^ 485.0503, found: 485.0525.


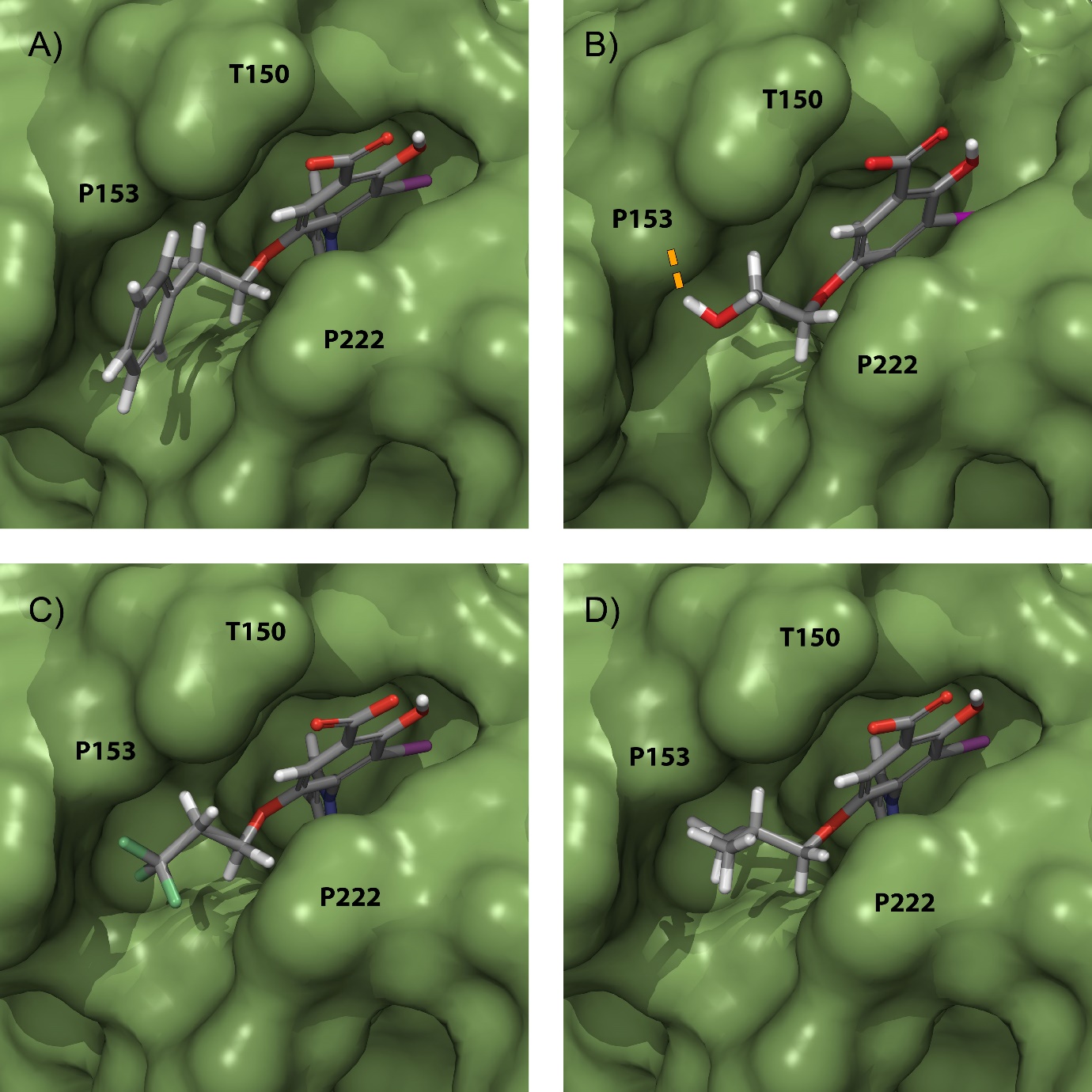


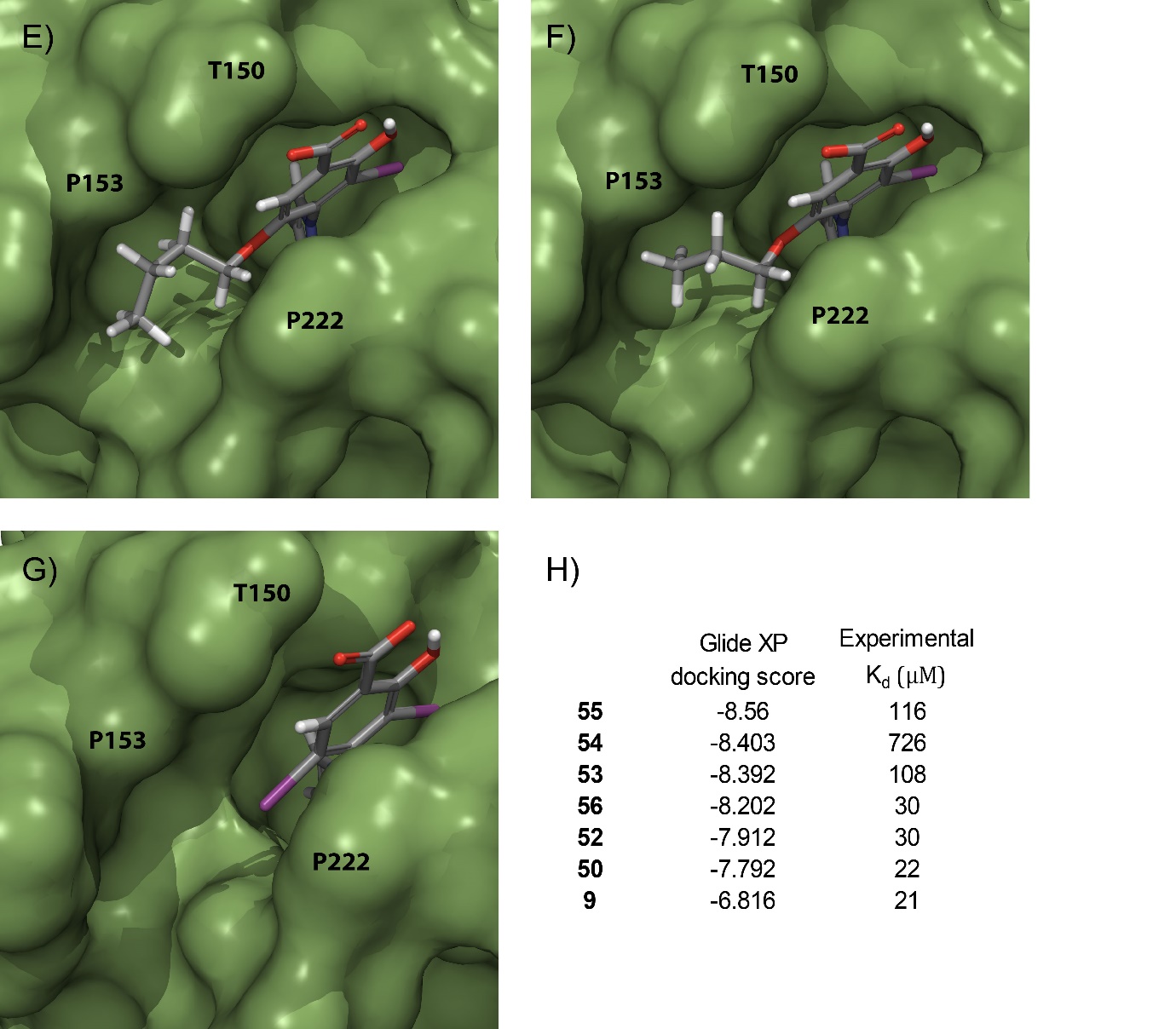


**Figure S1.** Glide docking studies. Predicted binding mode of representative ligands **55** (A), **54** (B), **53** (C), **56** (D), **52** (E), **50** (F) and reference ligand **9** (G), containing an oxyether linker to target subsite 2, and varying by their *O*-substitution. P153, P222 and T150 lining subsite 2 of the protein receptor (green, surface representation) are highlighted. A predicted hydrogen bond between the terminal hydroxyl group of **54** and the backbone of P153 is shown as orange dashed lines (B). Phenyl substituted compound **55** was the top hit among the >100 analogues docked. The docking scores and measured/experimental K_D_s are shown for each compound (H), and show poor correlation.

**
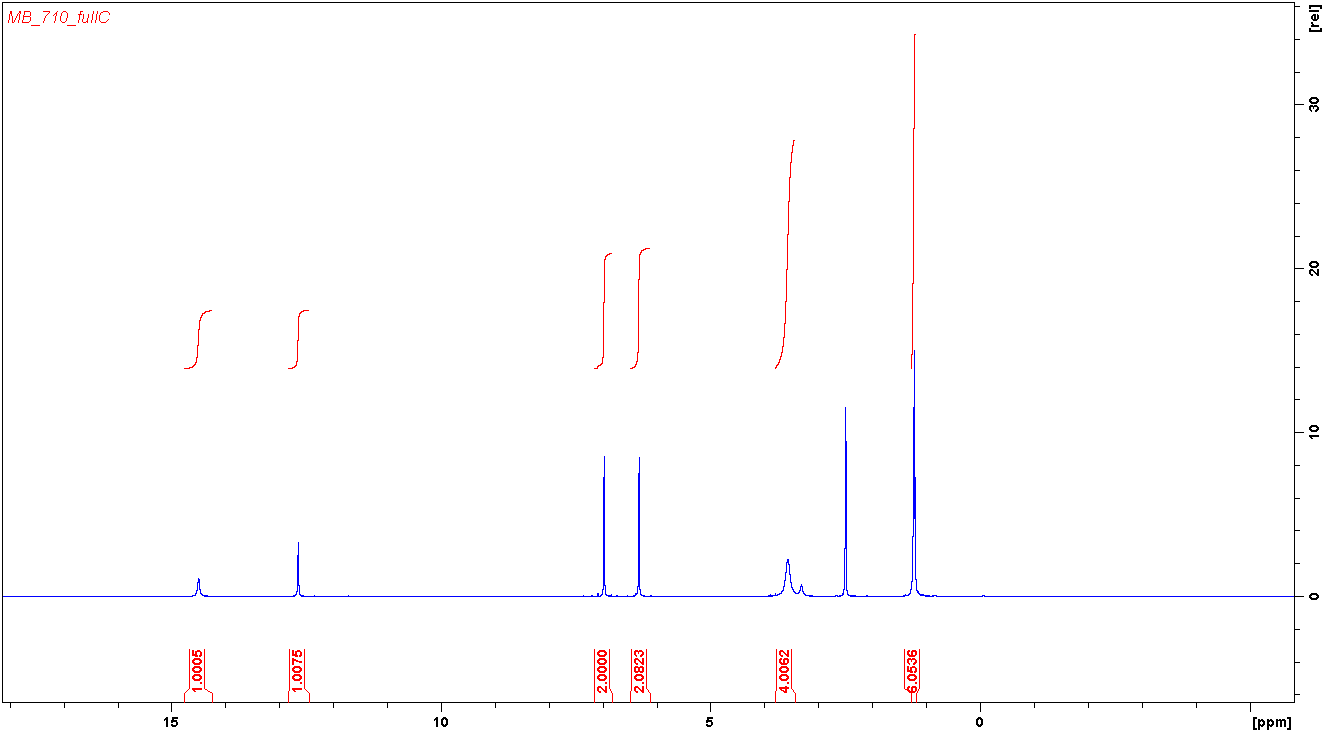
**

**
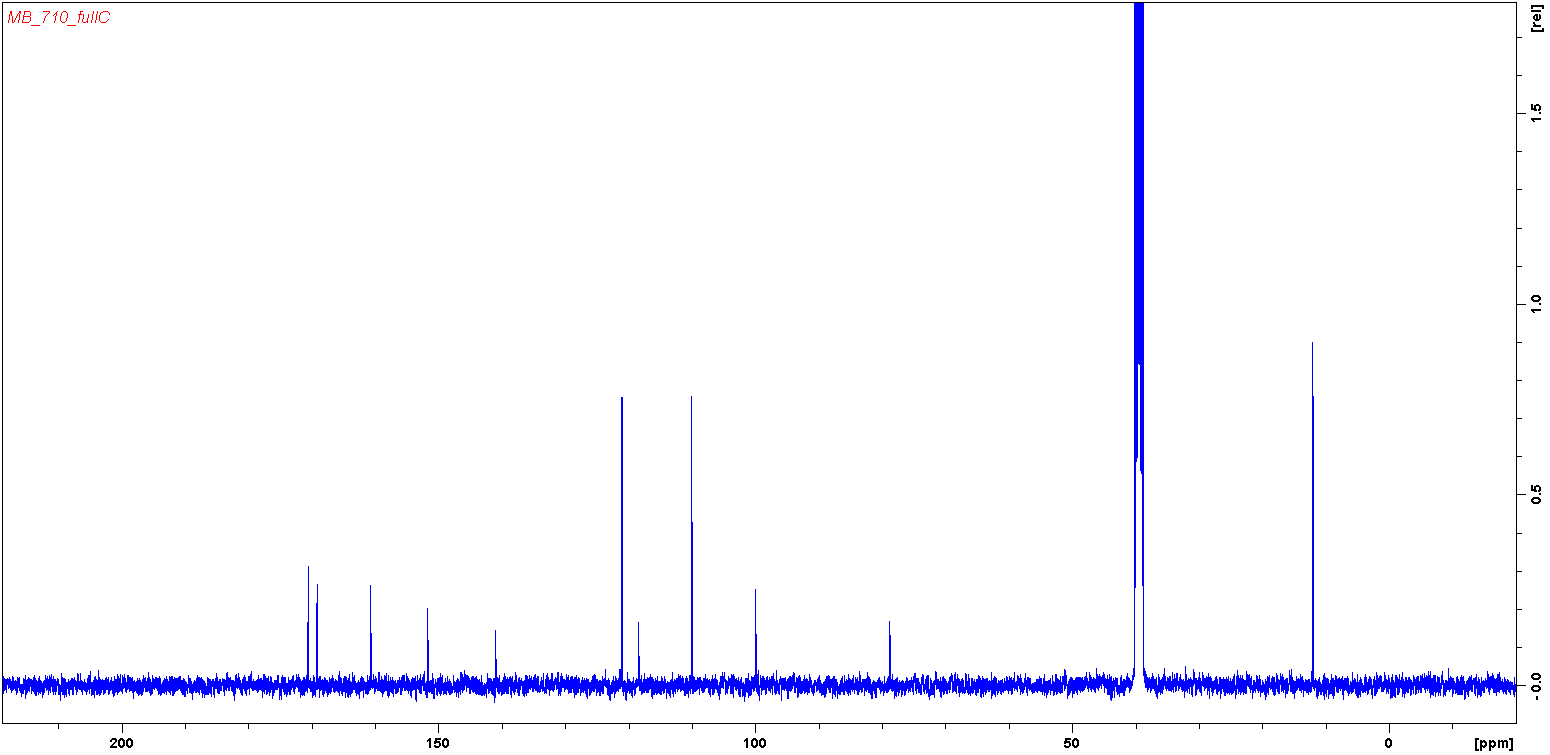
**

**Figure S2**. ^1^H and ^13^C NMR spectra (*d*^6^-dmso) of **MB_710 (70)**.

**
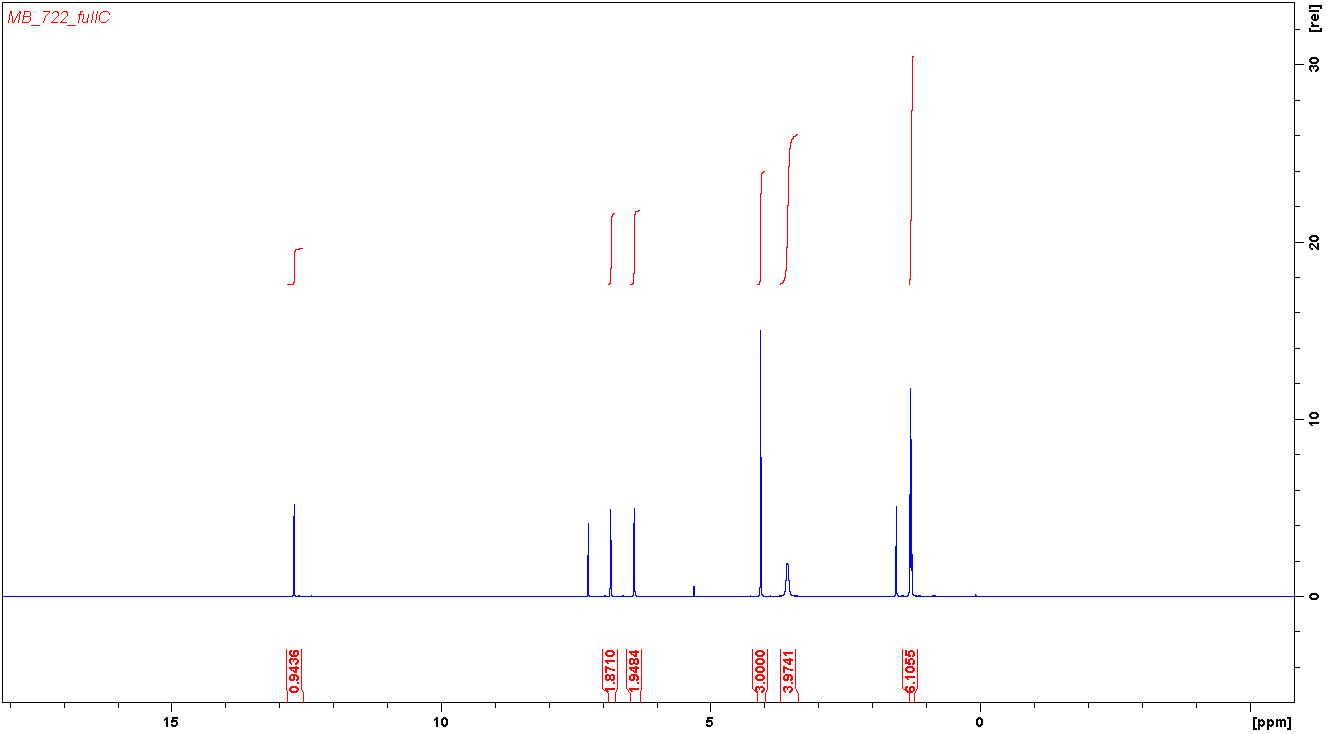
**

**
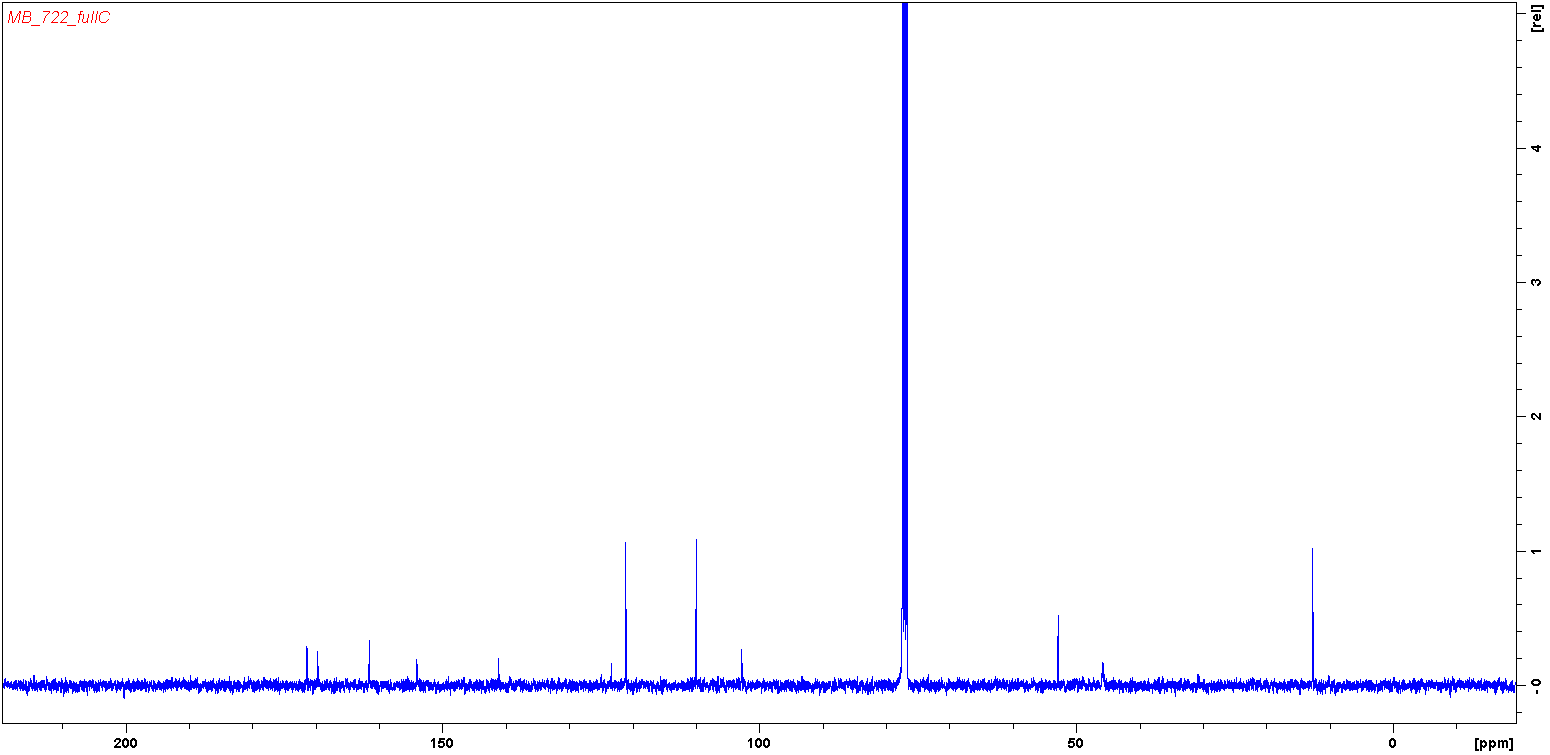
**

**Figure S3**. ^1^H and ^13^C NMR spectra (CDCl_3_) of **71**.

**
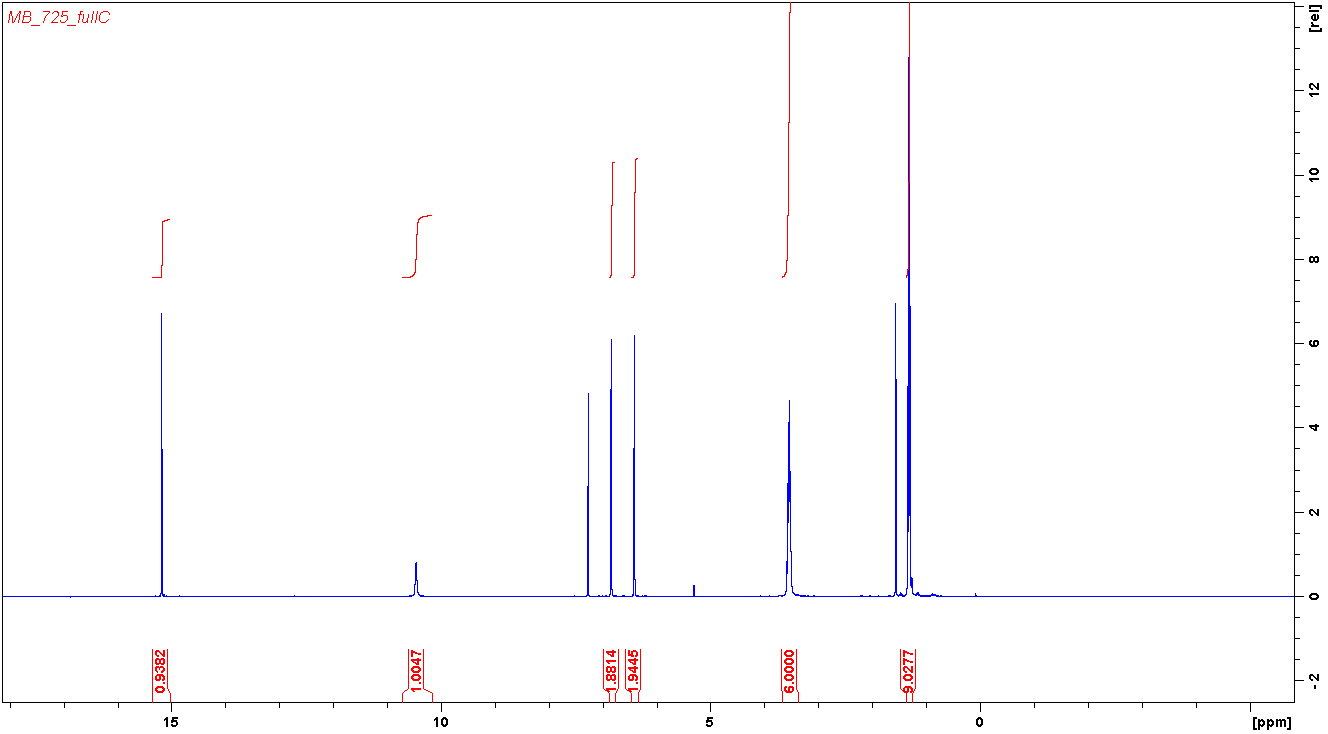
**

**
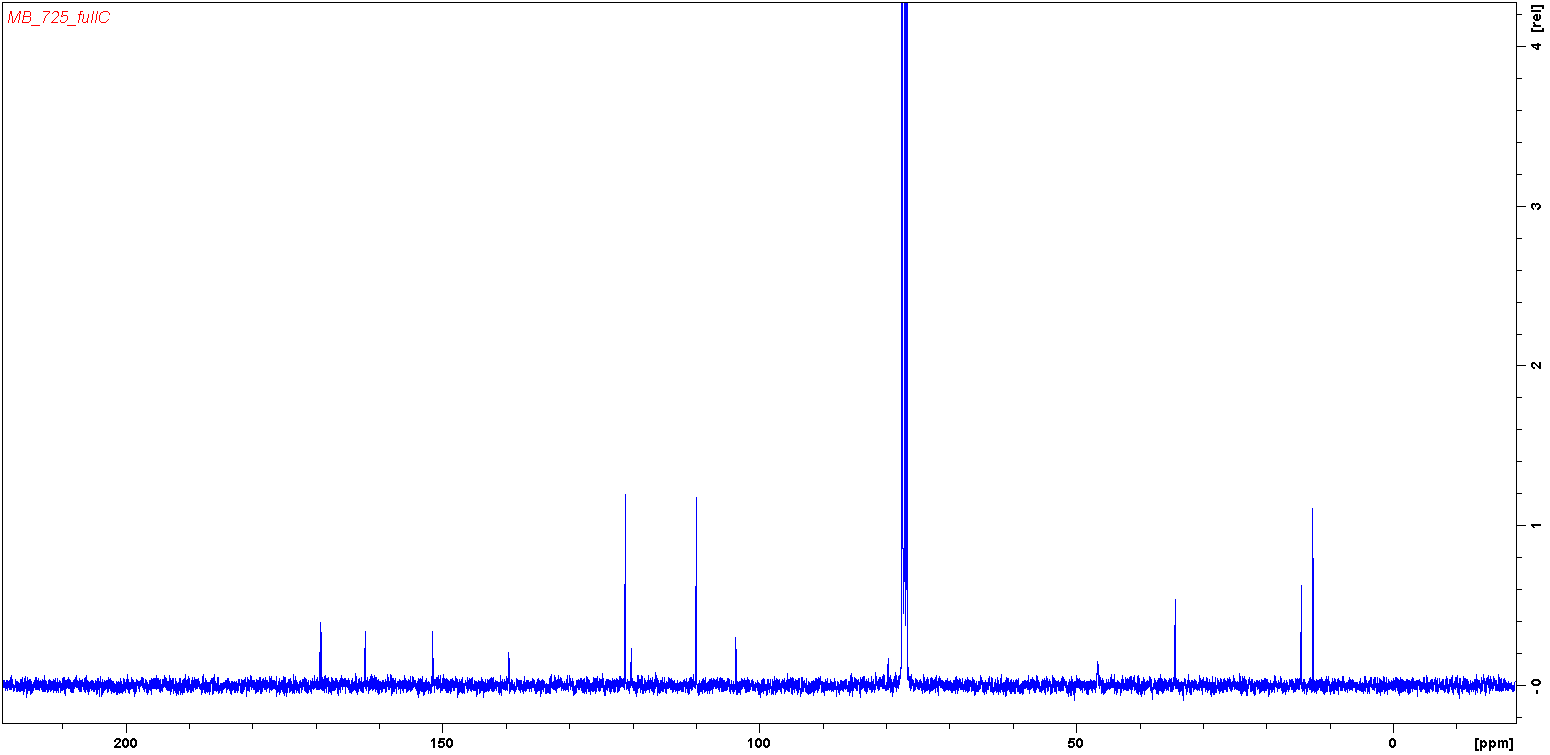
**

**Figure S4**. ^1^H and ^13^C NMR spectra (CDCl_3_) of **MB_725 (72)**

**
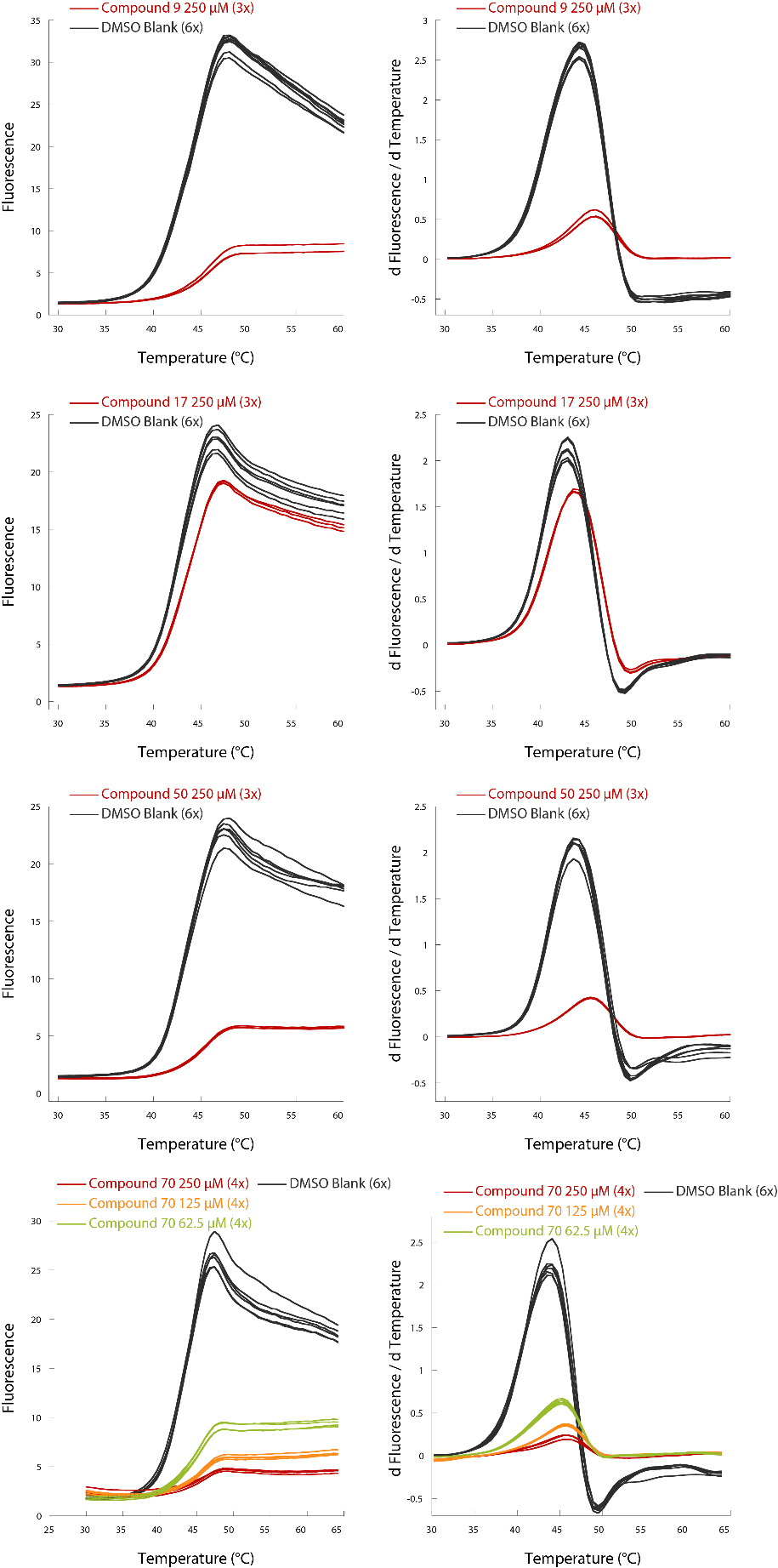
**

**Figure S5.** Differential scanning fluorimetry (DSF) graphs and depiction of their first derivative for p53-Y220C (8 μM) with compounds (250 μM) **9**, **17**, **52**, and **70** (**MB710**) or DMSO control. Standard deviation between measurements was low. Reduced fluorescence intensities were attributed to interaction (e.g. partial quenching) of the compound with the dye. This is well documented by Niesen.^1^ This is consistent with our ITC titrations, which could reliably and reproducibly be fitted to a 1 binding site model (see Figure S8 for a representative example).


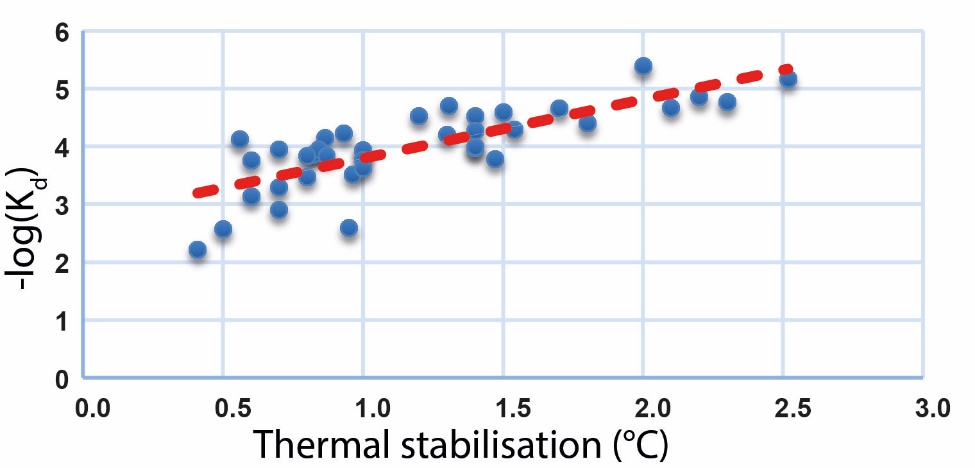


**Figure S6.** Biophysical studies. Correlation between pK_d_ and TS for demonstrates the robustness of our assays. Each blue spot represents one of 38 compounds assayed for both affinity (Y-axis) and TS (X-axis). Standard deviations were omitted for clarity, and were usually < 0.15°C (X-axis) and < 0.2 pK_D_ units for compounds displaying TS > 1.5 and pK_D_ > 4.

**
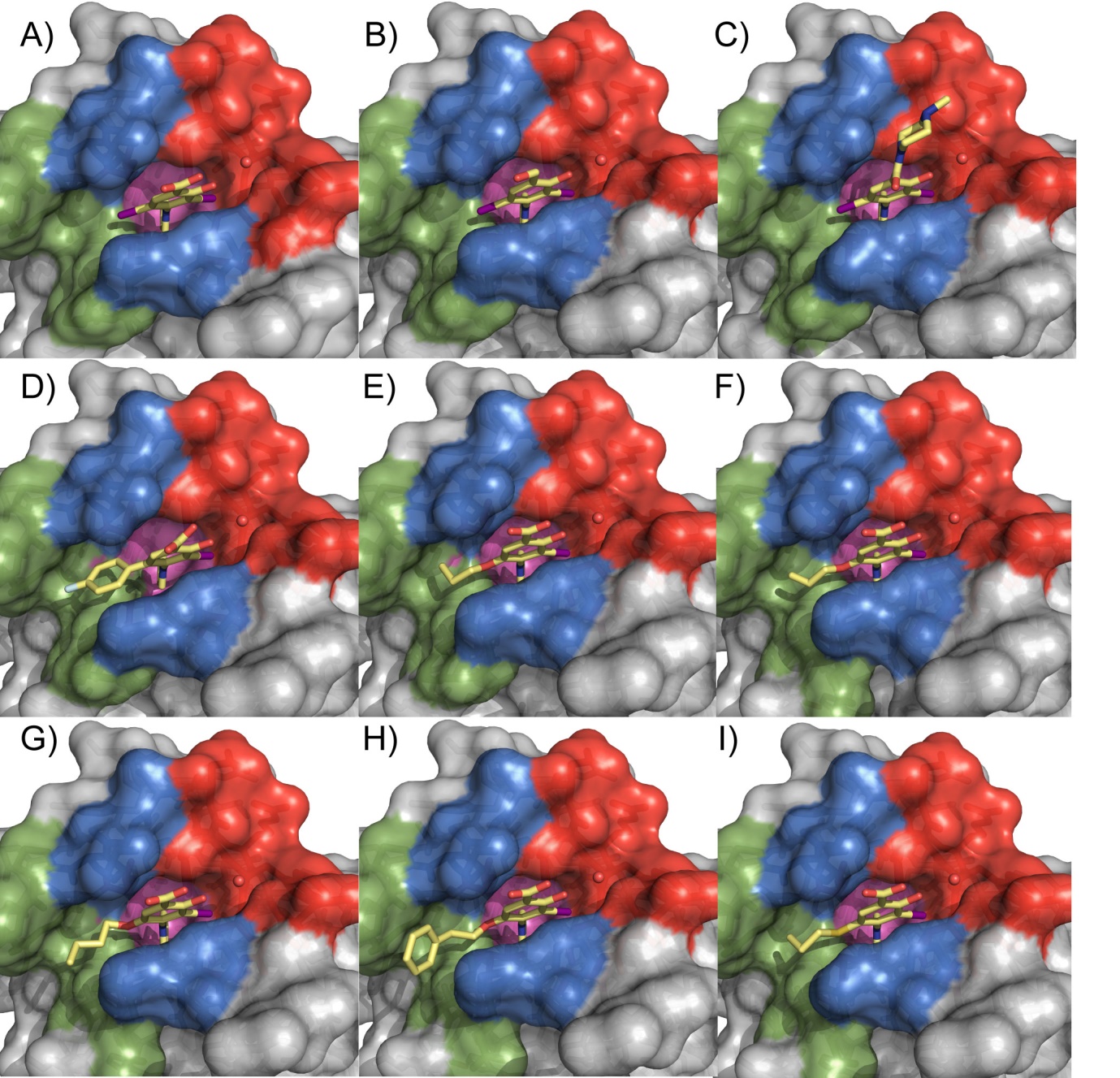
**

**Figure S7.** Co**-**crystal structure of QM-p53Y220C (surface representation) with A) **9**; B) **13**; C) **11**; D) **34**; E) **50**; F) **51**; G) **52** H) **55**; I) **60**. The colours attributed to the different subsites are consistent with those in Figure 2 of the manuscript.

**
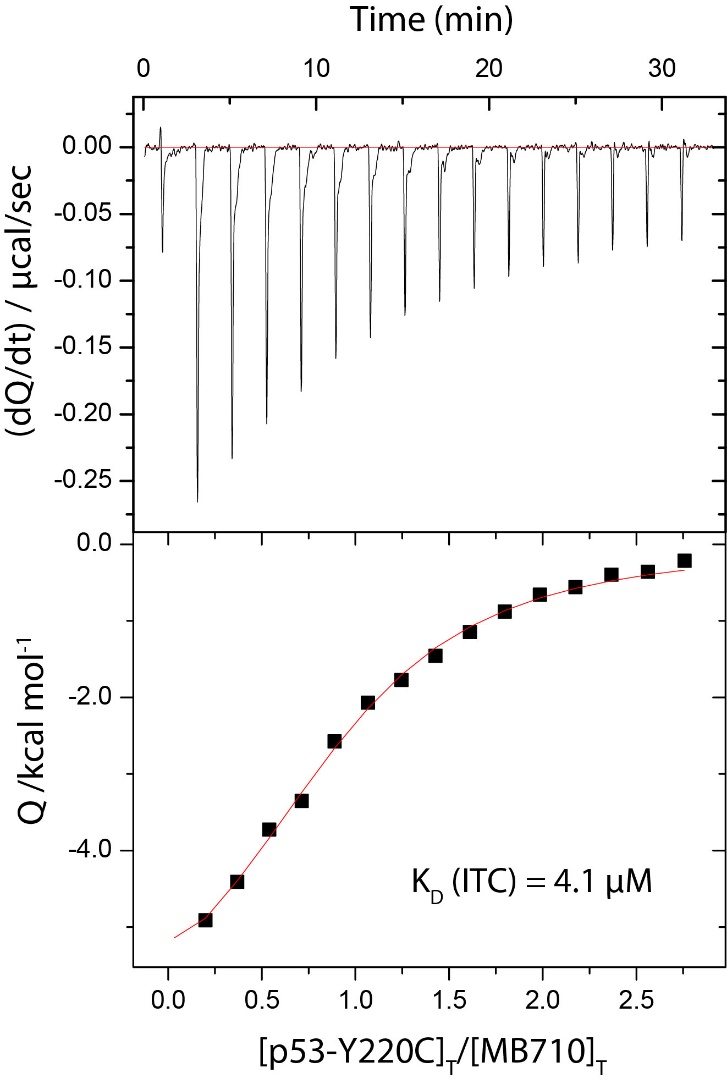
**

**Figure S8.** ITC titration curve for the binding of Stabilized p53-Y220C DBD (residues 94−312) to **MB_710**. Conditions: reverse titration of protein (190 μM) into compound (15 μM).

**
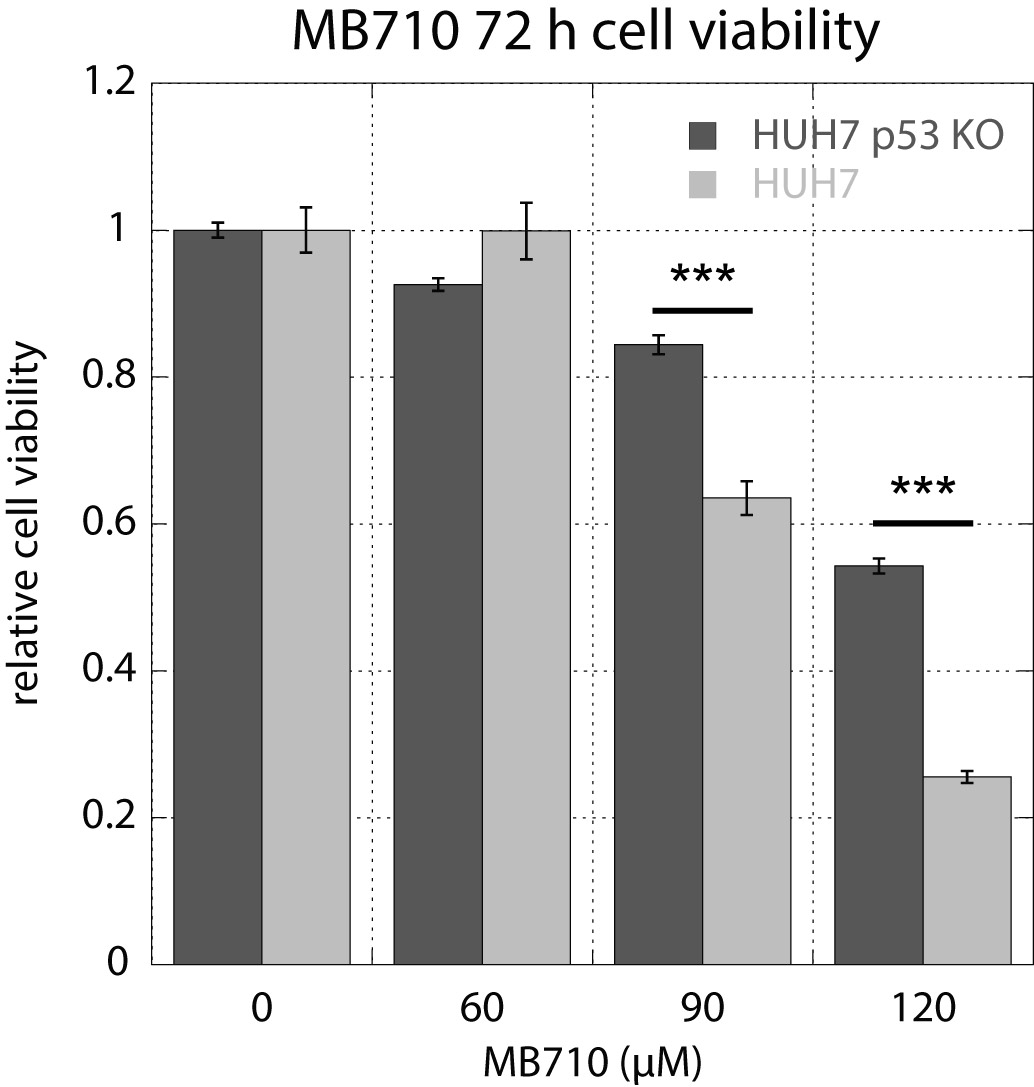
**

**Figure S9.** Assessment of p53-Y220C dependent effects of **MB710**. Treatment of HUH-7 (p53-Y220C) and HUH-7 p53-Y220C KO cell lines with **MB710** for 72h showed that the compound shows stronger cytotoxic effects in presence of p53-Y220C. Cell viability was measured in quadruplicate and normalized against the values of blank (viability = 1) and no cell (viability = 0) controls. Data are shown as mean ± SEM (Unpaired t-test; *p < 0.05; **p < 0.01; ***p < 0.001).

**Table S1.** Thermal stabilization and binding affinities of Y220C-mutant binding compounds as assessed by DSF and ITC/NMR.

| Cpd | R_1_ | R_2_ | **ΔT_m_ (°C)^a^**  **[ligand] = 250 μM** | ΔT_m_  STDEV  sample | ΔT_m_  STDEV  blank | T_m_  blank | ***K*_d_ (μM)** |
| --- | --- | --- | --- | --- | --- | --- | --- |
| **4** |  |  | **ND** | ND | ND | ND | **820^b^** |
| **8** | CO_2_Me | I | **-0.1^c^** | 0 | 0.092 | 44.2 | **ND** |
| **9** | CO_2_H | I | **1.8^d^** | 0.057 | 0.074 | 43.9 | **21^d^** |
| **10** |  | I | **1.3** | 0.06 | 0.11 | 43.7 | **ND^b^** |
| **11** |  | I | **1.2** | 0.10 | 0.11 | 43.7 | **30** |
| **12** | C(O)NHMe | I | **0.9** | 0.056 | 0.092 | 44.2 | **70** |
| **13** | CH_2_OH | I | **1.3** | 0 | 0.092 | 44.2 | **20^e^** |
| **15** | C(O)NHOH | I | **0.8** | 0.06 | 0.09 | 42.8 | **139** |
| **17** | CH_2_NMe_2_ | I | **0.9** | 0.05 | 0.09 | 42.8 | **60** |
| **21** | C(O)NHNH | I | **1.5** | 0.07 | 0.09 | 43.7 | **50** |
| **33** | COOH | Ph | **1.0** | 0.03 | 0.07 | 42.7 | **306** |
| **34** | COOH | 4-F-Ph | **0.8** | 0.06 | 0.07 | 42.7 | **113** |
| **35** | COOH | 4-OMe-Ph | **1.0** | 0.08 | 0.07 | 42.7 | **122** |
| **49** | COOH | OEt | **1.3** | 0.05 | 0.14 | 43.6 | **63** |
| **50** | COOH | OPr | **1.7** | 0 | 0.14 | 43.6 | **22** |
| **51** | COOH |  | **1.8** | 0.06 | 0.10 | 43.6 | **33** |
| **52** | COOH | O-*n*Bu | **1.4** | 0.06 | 0.10 | 43.6 | **30^e^** |
| **53** | COOH |  | **1.4** | 0.06 | 0.12 | 43.7 | **108** |
| **54** | COOH |  | **0.6** | 0.05 | 0.14 | 43.6 | **726** |
| **55** | COOH |  | **1.0** | 0 | 0.10 | 43.8 | **116** |
| **56** | COOH |  | **1.4** | 0 | 0.14 | 43.6 | **30** |
| **60** | COOH |  | **2.2** | 0.03 | 0.06 | 43.9 | **14** |

^a^measured by DSF using 8 μM protein and 10 x SYPRO Orange with ΔT_m_ values calculated as the average of quadruplicate measurements; ^b^data taken from ^2^; ^c^poor solubility; ^d^data taken from^3^; ^e^determined by HSQC-NMR with *K*_d_ values calculated as the average of at least three fits of peaks that are shifted by the compound as described previously.^2^

**Table S2.** X-ray data collection and refinement statistics of p53-Y220C ligand structures

| Compound | **11** | **13** | **34** | **50** | **51** | **52** | **55** | **60** | **MB710 (70)** |
| --- | --- | --- | --- | --- | --- | --- | --- | --- | --- |
| *Data Collection* |  |  |  |  |  |  |  |  |  |
| Space Group | *P*2_1_2_1_2_1_ | *P*2_1_2_1_2_1_ | *P*2_1_2_1_2_1_ | *P*2_1_2_1_2_1_ | *P*2_1_2_1_2_1_ | *P*2_1_2_1_2_1_ | *P*2_1_2_1_2_1_ | *P*2_1_2_1_2_1_ | *P*2_1_2_1_2_1_ |
| *a* (Å) | 65.16 | 65.15 | 65.19 | 65.18 | 65.08 | 65.00 | 65.13 | 65.13 | 65.00 |
| *b* (Å) | 71.09 | 71.17 | 71.12 | 71.19 | 71.18 | 71.06 | 71.06 | 71.40 | 71.09 |
| *c* (Å) | 105.26 | 105.34 | 105.31 | 105.32 | 105.18 | 105.10 | 105.06 | 105.23 | 105.23 |
| Molecules/AU | 2 | 2 | 2 | 2 | 2 | 2 | 2 | 2 | 2 |
| Resolution (Å)^a^ | 29.5-1.44  (1.52-1.44) | 29.5-1.43  (1.51-1,43) | 29.5-1.32  (1.39-1.32) | 29.6-1.36  (1.43-1.36) | 29.6-1.30  (1.37-1.30) | 29.4-1.38  (1.38-1.45) | 29.4-1.35  (1.42-1.35) | 29.5-1.32  (1.39-1.32) | 29.6-1.40  (1.48-1.40) |
| Unique reflections | 87,871 | 89,897 | 113,139 | 105,340 | 120,220 | 100,291 | 106,919 | 114,680 | 96,489 |
| Completeness (%)^a^ | 98.8 (99.0) | 98.8 (96.7) | 98.2 (97.0) | 99.7 (99.9) | 99.8 (99.9) | 99.7 (99.9) | 99.5 (99.7) | 99.3 (98.6) | 99.9 (99.7) |
| Multiplicity^a^ | 4.8 (4.8) | 6.4 (5.9) | 5.5 (5.4) | 5.1 (5.0) | 4.7 (4.7) | 4.5 (4.4) | 4.3 (4.2) | 5.0 (4.9) | 5.5 (5.3) |
| *R*_merge_ (%)^a^ | 5.2 (45,7) | 5.0 (38.8) | 5.6 (59.2) | 5.1 (52.4) | 5.0 (58.4) | 5.3 (54.3) | 4.8 (55.5) | 4.8 (49.0) | 5.6 (48.5) |
| Mean *I/σ(I)*^a^ | 14.3 (3.4) | 19.0 (4.9) | 16.2 (3.9) | 15.0 (3.0) | 13.7 (2.7) | 14.7 (3.5) | 14.9 (3.0) | 14.0 (3.1) | 15.7 (3.1) |
| Wilson *B* value (Å^2^) | 13.4 | 11.6 | 9.8 | 11.7 | 11.4 | 10.6 | 11.0 | 12.3 | 9.7 |
| *Refinement* |  |  |  |  |  |  |  |  |  |
| *R*_work_, (%)^b^ | 14.8 | 15.1 | 15.1 | 14.6 | 14.9 | 14.7 | 14.8 | 14.5 | 15.7 |
| *R*_free_, (%)^b^ | 17.8 | 17.4 | 17.2 | 16.4 | 16.5 | 16.7 | 16.6 | 16.0 | 18.7 |
| No. of atoms |  |  |  |  |  |  |  |  |  |
| Protein^c^ | 3114 | 3119 | 3103 | 3115 | 3125 | 3120 | 3099 | 3094 | 3123 |
| Zinc | 2 | 2 | 2 | 2 | 2 | 2 | 2 | 2 | 2 |
| Water | 420 | 462 | 471 | 405 | 404 | 402 | 404 | 405 | 425 |
| Ligands | 52 | 50 | 46 | 52 | 52 | 42 | 56 | 46 | 34 |
| RMSD bonds (Å) | 0.005 | 0.005 | 0.005 | 0.005 | 0.005 | 0.005 | 0.005 | 0.005 | 0.006 |
| RMSD angles (°) | 0.8 | 0.8 | 0.8 | 0.8 | 0.8 | 0.8 | 0.8 | 0.8 | 0.9 |
| Mean *B* (Å^2^) | 20.0 | 19.0 | 18.0 | 19.0 | 19.0 | 18.0 | 18.0 | 21.0 | 17.0 |
| PDB entry | 5O1A | 5O1B | 5O1C | 5O1D | 5O1E | 5O1F | 5O1G | 5O1H | 5O1I |

^a^Values in parentheses are for the highest-resolution shell.

^b^*R*_work_ and *R*_free_ = ∑||*F*_obs_| - |*F*_calc_||/∑|*F*_obs_|, where *R*_free_ was calculated with 5 % of the reflections chosen at random and not used in the refinement.

^c^Number includes alternative conformations.

**Table S3.** Description of cell lines

| cell line | p53 status | organism | tissue type | disease | ATCC/JCRB code |
| --- | --- | --- | --- | --- | --- |
| WI-38 | WT | human | lung | - | CCL-75 |
| HUH-6 | WT | human | liver | hepatoblastoma | JCRB0401 |
| NUGC-4 | WT | human | stomach | gastric adenocarcinoma | JCRB0834 |
| SW1088 | R273C | human | brain | astrocytoma | HTB-12 |
| HUH-7 | Y220C | human | liver | hepato cellular carcinoma | JCRB0403 |
| NUGC-3 | Y220C | human | stomach | gastric adenocarcinoma | JCRB0822 |
| BXPC3 | Y220C | human | pancreas | adenocarcinoma | CRL-1687 |

**References**

1. Niesen, F. H.; Berglund, H.; Vedadi, M., The Use of Differential Scanning Fluorimetry to Detect Ligand Interactions that Promote Protein Stability. *Nat Protoc.* **2007**, *2*, 2212-2221.

2. Wilcken, R.; Liu, X.; Zimmermann, M. O.; Rutherford, T. J.; Fersht, A. R.; Joerger, A. C.; Boeckler, F. M., Halogen-Enriched Fragment Libraries as Leads for Drug Rescue of Mutant p53. *J. Am. Chem. Soc.* **2012**, *134*, 6810-6818.

3. Joerger, A. C.; Bauer, M. R.; Wilcken, R.; Baud, M. G. J.; Harbrecht, H.; Exner, T. E.; Boeckler, F. M.; Spencer, J.; Fersht, A. R., Exploiting Transient Protein States for the Design of Small-Molecule Stabilizers of Mutant p53. *Structure* **2015**, *23*, 2246-2255.
